# Genomic modelling of the ESR1 Y537S mutation for evaluating function and new therapeutic approaches for metastatic breast cancer

**DOI:** 10.1038/onc.2016.382

**Published:** 2016-10-17

**Authors:** A Harrod, J Fulton, V T M Nguyen, M Periyasamy, L Ramos-Garcia, C-F Lai, G Metodieva, A de Giorgio, R L Williams, D B Santos, P J Gomez, M-L Lin, M V Metodiev, J Stebbing, L Castellano, L Magnani, R C Coombes, L Buluwela, S Ali

**Affiliations:** 1 Department of Surgery & Cancer, Imperial College London, Hammersmith Hospital Campus, London, UK; 2 School of Biological Sciences, University of Essex, Wivenhoe Park, Colchester, UK

## Abstract

Drugs that inhibit estrogen receptor-α (ER) activity have been highly successful in treating and reducing breast cancer progression in ER-positive disease. However, resistance to these therapies presents a major clinical problem. Recent genetic studies have shown that mutations in the ER gene are found in >20% of tumours that progress on endocrine therapies. Remarkably, the great majority of these mutations localize to just a few amino acids within or near the critical helix 12 region of the ER hormone binding domain, where they are likely to be single allele mutations. Understanding how these mutations impact on ER function is a prerequisite for identifying methods to treat breast cancer patients featuring such mutations. Towards this end, we used CRISPR-Cas9 genome editing to make a single allele knock-in of the most commonly mutated amino acid residue, tyrosine 537, in the estrogen-responsive MCF7 breast cancer cell line. Genomic analyses using RNA-seq and ER ChIP-seq demonstrated that the Y537S mutation promotes constitutive ER activity globally, resulting in estrogen-independent growth. MCF7-Y537S cells were resistant to the anti-estrogen tamoxifen and fulvestrant. Further, we show that the basal transcription factor TFIIH is constitutively recruited by ER-Y537S, resulting in ligand-independent phosphorylation of Serine 118 (Ser118) by the TFIIH kinase, cyclin-dependent kinase (CDK)7. The CDK7 inhibitor, THZ1 prevented Ser118 phosphorylation and inhibited growth of MCF7-Y537S cells. These studies confirm the functional importance of ER mutations in endocrine resistance, demonstrate the utility of knock-in mutational models for investigating alternative therapeutic approaches and highlight CDK7 inhibition as a potential therapy for endocrine-resistant breast cancer mediated by ER mutations.

## Introduction

As the major driver of breast cancer development and progression, estrogen receptor-α (ER) is the pre-eminent target in 80% of breast cancers. Inhibition of ER activity with anti-estrogens or aromatase inhibitors (AI) for preventing estrogen biosynthesis, reduces relapse and improves patient survival.^1, 2^ However, in many patients tumours progress on these therapies, where resistant tumours are mostly ER-positive and frequently responsive to changes in endocrine agent,^3, 4, 5, 6^ although typically with shorter periods of response to second and third line endocrine treatments. Efforts to identify changes in ER that functionally act in resistance have shown that mutations in the ER gene are rare in primary breast cancer.^7, 8, 9^ However, new findings conclusively demonstrate that the ER gene (ESR1) is frequently mutated in advanced breast cancer; combining data from different reports indicates that ESR1 coding region mutations feature in about 20% of AI-resistant breast cancer.^10, 11, 12, 13, 14^ The rarity of ESR1 mutations in primary breast cancer, as well as the lack of ESR1 mutations in matched primary samples from patients in which ESR1 mutations are evident after progression on endocrine therapies, indicates that treatment-selective pressures are likely to drive the acquisition of ESR1 mutations. Recent analyses of circulating tumour DNA further supports treatment-selective acquisition of ESR1 mutations,^15, 16^ and droplet digital PCR has identified ESR1 mutations in a proportion of primary tumours at very low mutant allele frequencies,^17^ suggesting that endocrine treatments may lead to selection of cancer cells with pre-existing ESR1 mutations.

The great majority of the mutations identified in advanced breast cancer occur in the ER ligand binding domain (LBD), with a ‘hotspot' at the consecutive amino acids L536, Y537 and D538, which map to the loop connecting α-helices 11 and 12. Structural analyses indicate that these residues control the agonist state of the LBD and that their mutation stabilizes the receptor in the agonist state, to promote co-activator recruitment^18, 19^ and thus aid transcription of ER target genes. Functional studies following ectopic expression of ER in which L536 or Y537 were substituted by other amino acids showed ligand-independent activation of estrogen-responsive reporter genes and interaction with co-activator proteins.^13, 20, 21, 22, 23, 24, 25^ Although ER mutated at these residues is inhibited by anti-estrogens, there is evidence for an attenuated response to anti-estrogens, at least for some substitutions.^10, 14, 26^ However, it is possible that the observed resistance is reflective of the fact that studies to date have employed ectopic over-expression of the mutant proteins.

CRISPR-Cas9-mediated genome editing provides a highly specific method for gene deletion and knock-in mutagenesis in mammalian cells,^27^ potentially allowing more faithful evaluation of the functional importance of gene mutations in model systems. Thus, CRISPR-Cas9-mediated introduction of mutations in the genomically encoded ESR1 gene would facilitate direct comparison of isogenic wild-type and mutant breast cancer cells towards developing a better understanding of the consequences of these mutations on response to endocrine therapies, and importantly, would enable evaluation of therapeutic approaches to target breast cancers featuring ESR1 mutations. Towards this end, we have targeted Y537, the most frequently mutated ER residue, in the estrogen-responsive and anti-estrogen-sensitive MCF7 cells. In MCF7 cells with a genomically encoded ER-Y537S mutation, we show ligand-independent recruitment of ER and regulation of gene expression in the absence of estrogen. Moreover, these cells grow in the absence of estrogen and show evidence of resistance to anti-estrogens.

Transcription regulation by ER requires cyclical association and dissociation from regulatory regions of target genes, in a process that is intimately linked with proteasomal degradation.^28, 29^ Phosphorylation of ER at Ser118 in the N-terminal transactivation function-1 (AF-1) plays a key role in ER recycling, by promoting interaction with the E3 ubiquitin ligase E6AP.^30^ The importance of Ser118 phosphorylation is underscored by an association between Ser118 phosphorylation and response to endocrine therapy.^31^ Moreover, P-Ser118 levels are elevated in tamoxifen-resistant breast cancer,^32^ implicating Ser118 phosphorylation in endocrine resistance. Estrogen binding results in recruitment of the basal transcription factor TFIIH through a direct interaction with the ER LBD to promote Ser118 phosphorylation by the cyclin-dependent kinase (CDK)7 kinase of TFIIH.^33, 34^ We show that CDK7 inhibition is effective in blocking growth of MCF7 ER-Y537S cells, demonstrating the utility of CRISPR-Cas9 generated breast cancer models of ER mutations for evaluating new therapeutic approaches for endocrine resistant breast cancer.

## Results

### CRISPR-Cas9-mediated generation of MCF7 cells with a genomically encoded Y537S mutation in the ESR1 gene

Searching a human exon CRISPR database^35^ identified four potential CRISPR sequences that target exon 8 of the ESR1 gene proximal to the Y537 codon. To identify the most appropriate CRISPR sequence, we assessed the activities of each CRISPR in the HCT116 cell line, which has been widely used for creating gene knockouts. Sanger sequencing of genomic DNA prepared 96 h after co-transfection of HCT116 cells showed that all four CRISPRs promoted indels, with CRISPR058819 being apparently the most efficient (Supplementary Figure 1). This screening approach provides a rapid method for evaluating CRISPR activity and on this basis CRISPR058819 was used for generating the Y537S mutation in MCF7 cells.

Donor DNA for gene targeting consisted of a cloned 1.8 kb fragment of the ESR1 gene, flanking the Exon 8 coding region. This was mutated to generate the Y537S mutation (TAT>TCT), together with a silent, single base change in the L536 codon (CTG>CTC) and several silent changes to destroy the CRISPR058819 PAM recognition sequence (Supplementary Figures 2A and B). The latter substitutions prevent CRISPR targeted cleavage of the donor template DNA by Cas9, provide a ‘tag' that distinguishes targeted from any endogenously generated ESR1 exon 8 mutations and assist with specific PCR screening for the MCF7-Y537S mutation.

**Figure 1:**
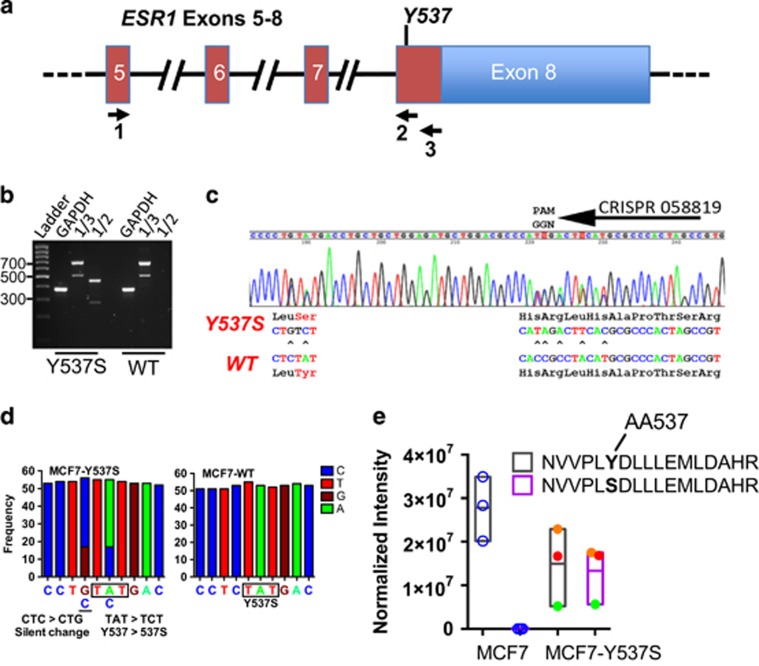
CRISPR-Cas9-directed generation of the Y537S mutation in the ESR1 gene in MCF7 breast cancer cells. (**a**) Schematic representation of the ESR1 gene, exons 5-8, annotated for the positions of PCR primers used for RT-PCR analysis. (**b**) RT-PCR of MCF7 (WT) and MCF7-Y537S cell lines using primers in **a**. Expression of wild-type and mutant ER alleles was confirmed by RT-PCR, using primers in ESR1 Exon 5 (Primer 1, 5′-CCAGGGAAGCTACTGTTTGC-3′) and Exon 8 (Primer 3, 5′-GATGCATGCCGGAGTGTATG-3′), which generate a 700 bp product. The ESR1 transcript arising from the Y537S mutant allele was amplified as a 466 bp product using the exon 5 primer and the knock-in-specific primer. (Primer 2, 5′-TAGTGGGCGCGTGAAGTCTA-3′). (**c**) Sequencing chromatogram of the RT-PCR products for the exon 8 coding region for MCF7-Y537 S cells, showing expression of both mutant and wild-type alleles. (**d**) Frequency of RNA-seq reads for the Y537 and 537S codons in MCF7 and MCF7-Y537S cells. (**e**) Normalized intensity of ER tryptic peptide containing amino acid 537 from liquid chromatography mass spectrometry. Results are shown for three independent immunoprecipitated ER samples for MCF7 and MCF7-Y537S lines. No signal was obtained for the mutant peptide in MCF7 cells. For the MCF7-Y537S samples, the normalized intensity for each replicate sample is shown by circles of the same colour, demonstrating similar amounts of the two peptides in MCF7-Y537S cells.

Following co-transfection of MCF7 cells with the donor template, CRISPR058819 and hCas9 plasmids, single colony cloning and PCR screening were used to isolate MCF7 cells with the ER-Y537S mutation. PCR of genomic DNA prepared from expanded clones with CRISPR058819 target site mutant-specific PCR primers identified positive clones, which were confirmed with DNA sequencing. Interestingly, of six clones with successful knock-in mutations, all but one clone (MCF7-Y537S [NF2-A4]) featured indels that caused frameshift mutations in the second allele. Clones with indels were excluded from further analysis because of the potential for truncated ER proteins. DNA sequencing of the MCF7-Y537S [NF2-A4] (hereafter referred to as MCF7-Y537S) genomic DNA was consistent with a heterozygous line with one mutant allele (Supplementary Figure 2C). Reverse transcriptase (RT)-PCR of exons 5-8 with a primer that specifically amplifies the mutant allele (primer 2) gave a product only for MCF7-Y537S cells (Figure 1b). The RT-PCR products for primers 1/3 were similar for both wild-type and MCF7-Y537S RNA. Sanger sequencing of the latter PCR product showed expression only of wild-type and Y537S mutant ER in MCF7-Y537S cells (Figure 1c). Note that the smaller PCR product, which is consistent in size with an alternatively spliced ER mRNA lacking exon 7 sequences, has been described previously.^7^ The exon-7 deleted mRNA is present at similar levels in both lines, suggesting that introduction of the Y537S mutation does not alter ER gene expression patterns. Quantification of RNA-seq reads confirmed that the MCF7-Y537S line expresses only wild-type and Y537S mutant ER mRNA (Figure 1d). To demonstrate that MCF7-Y537S cells express the mutant protein, ER was immunoprecipitated and immunoprecipitates were analysed by liquid chromatography mass spectrometry following tryptic digestion. A tryptic peptide corresponding to wild-type, but not mutant ER, was detected in MCF7 cells (Figure 1e, Supplementary Figure 2D). In MCF7-Y537S cells, both peptides were detected, with levels of wild-type and mutant peptides being similar in each replicate. This further confirms similar levels of expression for both ER proteins in MCF7-Y537S cells.

### MCF7-Y537S cells grow in the absence of estrogen and are partially resistant to anti-estrogens

**Figure 2:**
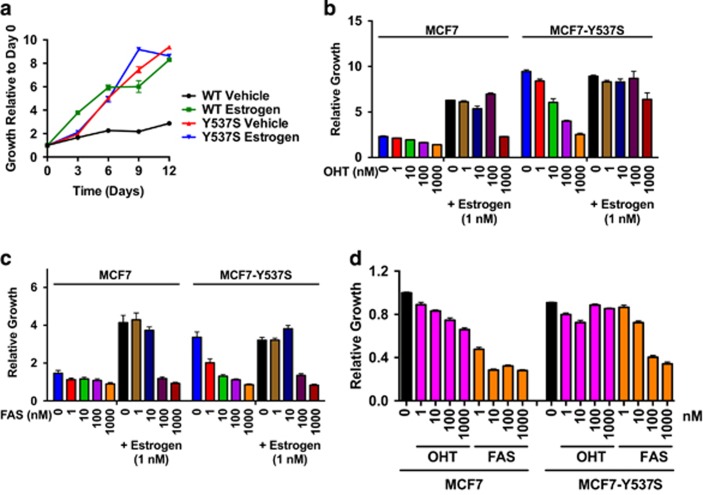
MCF7-Y537S cells grow in an estrogen-independent manner. (**a**) MCF7 (WT) and Y537S cells were grown in the absence or presence of 10 nM estrogen over 12 days. (**b**, **c**) The cells were grown in the absence of estrogen (vehicle) or in the presence of 10 nM estrogen, together with 0, 1, 10, 100 or 1000 nM 4-hydroxytamoxifen (OHT; **b**) or faslodex (FAS; **c**). Mean growth at day 12 ±s.e.m. is shown relative to day 0 (*n*=6). (**d**) MCF7 and MCF7-Y537S cells, cultured in DMEM+10%FCS, were treated with OHT or FAS at the concentrations shown (nM). The graph shows mean growth at day 12±s.e.m. (*n*=6).

Whereas MCF7-WT growth was estrogen-dependent, growth of MCF7-Y537S cells was similar in the presence or absence of estrogen and comparable to that of estrogen-treated MCF7-WT cells (Figure 2a). In the absence of estrogen, 4-hydroxytamoxifen (OHT) inhibited growth of MCF7-Y537S cells in a dose-dependent manner (Figure 2b), but complete growth inhibition was only achieved for 1 μM OHT (3.7-fold). To investigate the effects of OHT in the presence of estrogen, the cells were treated with increasing doses of OHT in the presence of 10 nM estrogen. MCF7 cell growth was inhibited 2.8-fold at 1 μM OHT, this being comparable to the minimal growth seen in estrogen-depleted medium. By contrast, even with 1 μM OHT, growth of MCF7-Y537S growth was only modestly repressed (1.4-fold inhibition).

Faslodex (FAS) was a more effective inhibitor of MCF7-Y537S growth (Figure 2c). As can be seen, only 1 μM FAS was effective at inhibiting MCF7-WT cell growth in the presence of 10 nM estrogen, where it led to a 4.3-fold inhibition of estrogen stimulated growth, this being comparable to growth for this line in estrogen deprived medium. MCF7-Y537S cells were similarly growth inhibited by 1 μM FAS in the presence of 10 nM estrogen, this causing a 4.1-fold inhibition of the growth seen in 10 nM estrogen alone. For MCF7-Y537S cells, all concentrations of FAS tested inhibited the potent growth of this line in the absence of estrogen, with 10 nM-1 μM FAS leading to a 5.5-6.5-fold inhibition.

In full medium conditions (Figure 2d), MCF7-WT cells showed a titratable response to OHT, with growth in 1 μM OHT being inhibited by 1.5-fold over the untreated cells. In contrast, growth of the MCF7-Y537S line was unaffected by OHT. Under full medium conditions, MCF7-WT cells also showed potent growth inhibition by FAS over the range 1 nM-1 μM, with 10 nM–1 μM FAS resulting in >3-fold inhibition over untreated cells. The response to FAS by the MCF7-Y537S line under the same conditions was less pronounced, with 10 nM FAS showing only a 1.2-fold inhibition, but growth was inhibited fully with 1 μM FAS (2.6-fold over vehicle-treated cells; Figure 2d).

Taken together, these findings provide evidence that breast cancer cells harbouring single allele ER-Y537S mutations acquire both a resistance to anti-estrogens and a capacity to grow efficiently under estrogen-depleted conditions.

### ER-Y537S is recruited to ER binding regions in an estrogen-independent manner to promote co-activator recruitment and histone modification

**Figure 3:**
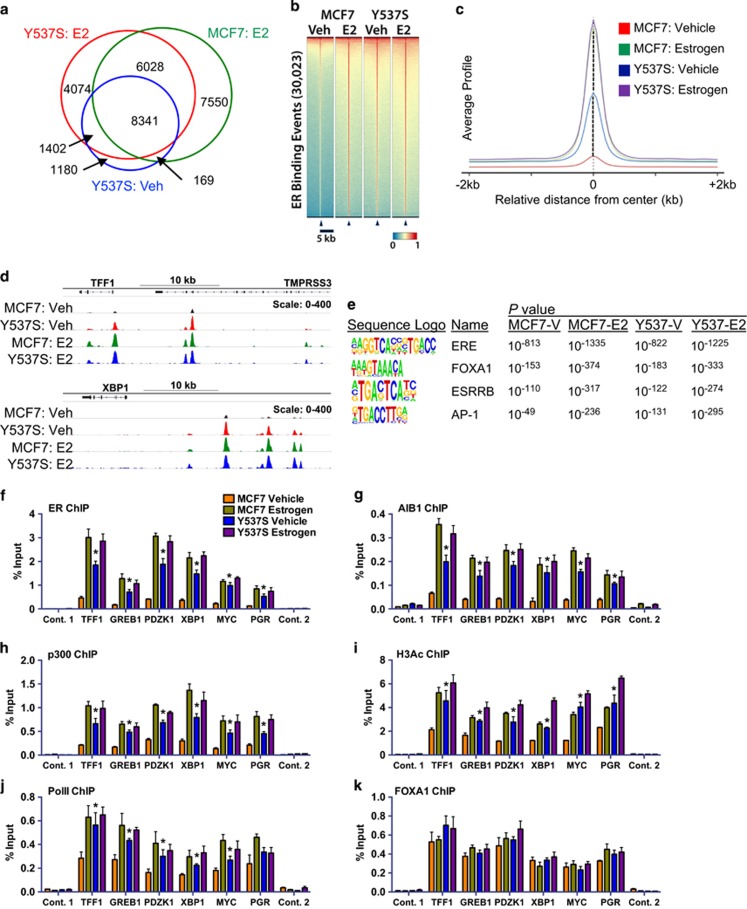
ER-Y537S mutant is globally recruited to ER binding regions in the absence of estrogen. (**a**) Overlap of significant ER binding sites as called by MACS analysis (*P<*1 × 10^−5^). (**b**) Heat map for ER binding events in MCF7 and MCF7-Y537S cells under vehicle (Veh) and estrogen (E2) treatment conditions. (**c**) Genome-wide profile of ER binding. (**d**) ChIP-seq tracks showing ER binding at two ER target genes. (**e**) Shown are the top enriched transcription factor binding motifs obtained using HOMER.^64^ See Supplementary Information for a complete list of motifs enriched in MCF7-WT and mutant cells. (**f**–**k**) ChIP was performed following treatment of MCF7 and Y537S cells cultured in hormone-depleted medium and treated with 10 nM estrogen for 45 min. Real-time PCR was used to determine enrichment at ER binding regions. PCR for two control regions near the TFF1 (control 1) and the PGR gene (control 2) is also shown. Bar charts represent the mean, ±s.e.m. (*n*=3). Asterisks represent statistically significant differences (unpaired *t*-test, *P<*0.05) for vehicle-treated MCF7-Y537S cells compared with MCF7 cells.

The above findings suggest that the ER-Y537S mutation promotes estrogen-independent expression of ER target genes. Towards addressing this directly, we performed ER ChIP-seq (Supplementary Figure 3). Peak calling identified 5930 and 22 088 ER binding sites in MCF7 cells in the absence and presence of estrogen, respectively. By contrast, there were 11 092 ER binding regions in vehicle-treated MCF7-Y537S cells, most of which were shared with estrogen-treated MCF7-Y537S (88% (9743/11 092) and MCF7-WT (77% (8510/11 092) cells (Figure 3a). Additional peaks were called for estrogen-treated MCF7-Y537S cells, which were also present in estrogen-treated MCF7 cells. A heat map comparison of ER binding events between the lines showed that the binding profiles in unstimulated Y537S cells are similar to that seen in estrogen-induced MCF7 cells (Figure 3b), as demonstrated in plots of average peak profiles, which showed a considerably greater magnitude of ER binding in the absence of estrogen in MCF7-Y537S, than in WT cells (Figure 3c), as exemplified for the TFF1 and XBP1 genes (Figure 3c). Motif enrichment analysis did not suggest that the Y537S mutation causes ER binding to new sites (Figure 3e; Supplementary Table 2). In all conditions, the most enriched binding motifs were the ER binding site (ERE), followed by FOXA1, AP-1 and GATA3 binding sites.

ChIP-qPCR confirmed ER recruitment in an estrogen-independent manner in MCF7-Y537S cells (Figure 3f). Recruitment of the transcriptional co-activators AIB1 and p300 was also estrogen-independent (Figures 3g and h). Histone H3 acetylation and RNA polymerase II (PolII) were also elevated at ER binding regions in MCF7-Y537S cells in the absence of estrogen (Figures 3i and j), suggestive of estrogen-independent transcription in this line. FOXA1 recruitment was unaffected by the Y537S mutation (Figure 3k), which is in keeping with its described role as a pioneer factor that pre-exists at ER binding regions and which acts to direct ER recruitment to chromatin.^36^

### ER target gene expression is ligand-independent and is frequently enhanced in MCF7-Y537S cells

**Figure 4:**
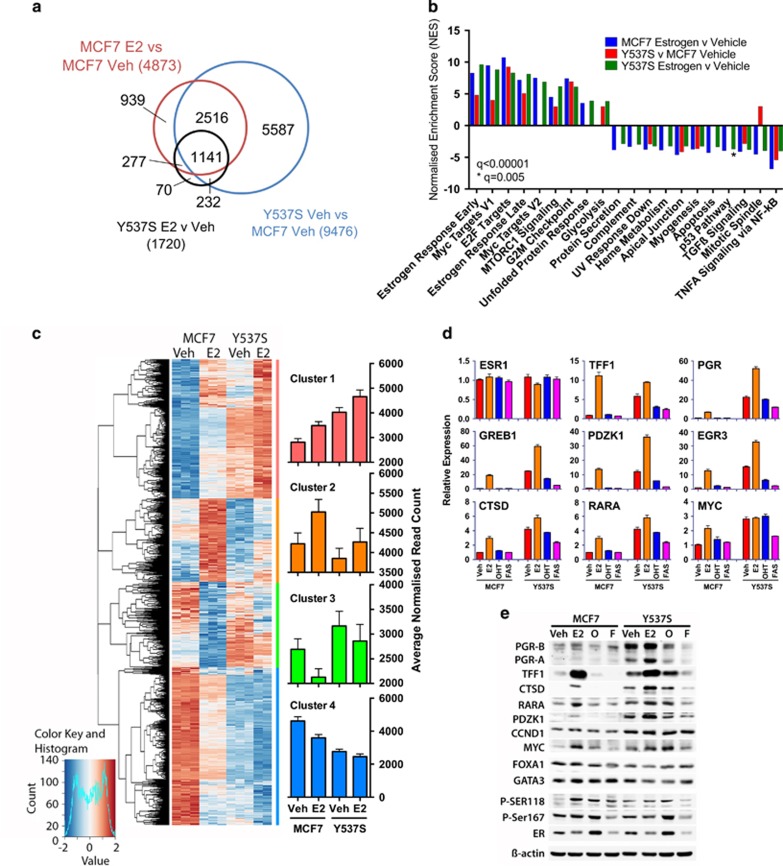
The Y537S mutation promotes estrogen-independent expression of ER target genes in breast cancer cells. (**a**) RNA-seq was performed using three replicate samples of hormone-depleted MCF7 and MCF7-Y537S cells, following addition of vehicle or estrogen for 8 h. Shown is a Venn diagram comparing differentially regulated genes (padj<0.05) identified from RNA-seq data. (**b**) The bar chart shows the normalized enrichment scores (NES) from Gene set enrichment analysis (GSEA) for ‘Hallmark' signalling pathways that are significantly up- or downregulated (*q*<0.00001) in pair-wise comparisons of the differentially regulated gene sets. (**c**) Hierarchical cluster analysis of genes that are differentially regulated in MCF7 cells ±E2 (padj<0.05) and for which an ER binding site is observed within 100 kb of the transcription start site. Bar charts show average normalized counts for all genes in the clusters. (**d**) E2 (1 nM), OHT (10 nM) or FAS (10 nM) were added to hormone-depleted MCF7 and MCF7-Y537S cells. Shown is RT-qPCR analysis of gene expression using RNA prepared 16 h following addition of ligands, relative to the vehicle-treated MCF7 cells (*n*=3). (**e**) Immunoblotting was performed using cell lysates prepared 24 h following addition of ligands.

To determine the consequence of the Y537S mutation on ER target gene expression, we performed RNA-seq for MCF7 and MCF7-Y537S cells. Treatment of cells with estrogen for 8 h was chosen following evaluation of the expression of well-known ER target genes in a time course of estrogen treatment (Supplementary Figure 4A). This time point showed robust stimulation of ER target gene expression in MCF7 cells, but would be expected to show limited estrogen stimulation of indirect targets. For each condition, three independent replicate samples were analysed by RNA-seq. Analysis with the RNASeqPower package in R for our data showed that three replicates gave a power >0.8 for a fold change of 1.5. As the sequencing data for one of the three replicates for MCF7-Y537S cells treated with estrogen failed quality control, two data sets were available for analysis. In MCF7 cells, 4873 genes were differentially regulated by treatment with estrogen (padj<0.05; Figure 4a). Comparison of vehicle-treated MCF7 and MCF7-Y537S cells showed that the majority (75% 3657/4873) of genes that are estrogen-regulated in MCF7 cells are differentially regulated in MCF7-Y537S cells. Of these genes, only 31% (1141/3657) are estrogen regulated in MCF7-Y537S cells (see also supplementary Figure 4B), suggesting that most estrogen-responsive genes are ligand-independent in MCF7-Y537S cells. Gene set enrichment analysis (GSEA) identified pathways including early and late estrogen responsive targets (Figure 4b; Supplementary Table 3). Also enriched were pathways associated with cell proliferation, including E2F and Myc targets and the G2M checkpoint. Interestingly, several GSEA pathways enriched with estrogen treatment of MCF7 cells remained estrogen-regulated in MCF7-Y537S cells. For the most part, these were pathways that were downregulated in estrogen-treated MCF7 cells and included protein secretion, heme metabolism and apoptosis. There was also the suggestion of further augmentation of ER regulated gene expression in the presence, compared with the absence of estrogen in MCF7-Y537S cells, which is in agreement with the estrogen-stimulated ER recruitment observed for the MCF7-Y537S ER ChIP-seq.

Analysis of the differentially regulated genes using RNASeqPower indicated a fold change cut-off >1.5 was required for power >0.8. In all, 5407 genes were differentially regulated with FC>1.5 and padj<0.05. Hierarchical cluster analysis of the differentially regulated genes showed segregation in two large clusters; genes that showed estrogen stimulation in MCF7 cells and the expression of which was elevated in MCF7-Y537S cells and a second group comprising genes whose expression was reduced in estrogen-treated MCF7 cells and which were further repressed in MCF7-Y537S cells (Supplementary Figure 4C).

To examine regulation of direct ER target genes, we integrated the ER ChIP-seq data with the estrogen responsive genes in MCF7 cells (padj<0.05 from RNA-seq, above), using the Binding and Expression Target Analysis package.^37^ ChIP-seq and RNA-seq integration identified 1559 genes with an ER peak within 100 kb of transcription start sites. Hierarchical cluster analysis of the RNA-seq data for these genes identified four main groups (Figure 4c; Supplementary Table 4). Cluster 1 comprised genes whose expression is stimulated by estrogen in MCF7 cells. Expression of genes in this cluster was elevated in vehicle-treated MCF7-Y537S cells and was further increased with estrogen treatment. Genes in this cluster include well-characterized ER target genes, such as PGR, TFF1, CTSD, GREB1, NRIP1, XBP1, EGR3, CA12, PDZK1, IGFR, AREG and H19. As many genes are repressed by estrogen in breast cancer cells, as are stimulated^38^ and cluster 4 incorporates genes that are repressed by estrogen in MCF7 cells. Expression of cluster 4 genes was generally lower in MCF7-Y537S cells than in vehicle-treated MCF7 cells and was further reduced in estrogen-treated MCF7-Y537S cells. This is exemplified by ERBB2,^39^ the expression of which was 0.8-fold in estrogen treated, compared with vehicle-treated MCF7 cells and was reduced to 0.7- and 0.6-fold in vehicle- and estrogen-treated MCF7-Y537S (Supplementary Table 4). The other two clusters include genes whose expression is estrogen-stimulated and -repressed in MCF7 cells. Genes in these two clusters exhibited reduced estrogen responsiveness in MCF7-Y537S cells; expression in the absence of estrogen was similar to expression levels in vehicle-treated MCF7 cells.

Forty per cent of the genes that were differentially regulated between MCF7 and MCF7-Y537S cells were estrogen regulated in MCF7 cells and, as described above, are likely to mainly constitute direct ER target genes. The remaining differential genes may be involved in signalling pathways downstream of direct ER targets. To assess this, we analysed RNA-seq data for MCF7 that were cultured long-term in full medium.^40^ Most of the genes whose expression was altered following 8 h estrogen treatment in MCF7 cells were identified in vehicle-treated MCF7-Y537S and in MCF7 cells cultured in full medium (2884 genes; Supplementary Figure 5A). Indeed, ‘estrogen response early' was the most significantly enriched GSEA pathway in this gene set (Supplementary Figure 5B). Of the remaining genes identified in MCF7-Y537S cells, the majority (~70%), are also observed for the full medium conditions. This set of genes were enriched in signalling pathways associated with the G2M checkpoint and mitotic spindle, as well as metabolic pathways including fatty acid metabolism, oxidative phosphorylation and glycolysis (Supplementary Figure 5C). Analysis of an independent RNA-seq study where MCF7 cells were cultured in estrogen-depleted medium and treated with estrogen for 24 h^41^ gave very similar results (Supplementary Figures 5D-F). Together, these analyses show that estrogen regulated gene expression is a dominant feature that defines the MCF7-Y537S transcriptome.

To validate the RNA-seq findings, we focused on genes in cluster 1, as these include many of the best characterized ER target genes in breast cancer. In hormone-depleted MCF7-Y537S cells, mRNA levels of these genes were high in the absence of estrogen, at levels similar to those observed for estrogen-treated WT cells (Figure 4d). As observed for RNA-seq, expression of most genes was further stimulated by estrogen in MCF7-Y537S cells, to levels considerably higher than those achieved in estrogen-treated WT cells. Particularly striking is PGR, mRNA levels of which were 3.3- and 7.6-fold higher in the absence and presence of estrogen, respectively, compared with PGR expression in estrogen-treated MCF7 cells. This was reflected in the remarkably elevated levels of both PGR isoforms, PGR-A and PGR-B, in MCF7-Y537S cells, compared with WT cells (Figure 4e). In full medium conditions, expression of these ER targets was also elevated in MCF7-Y537S mutant, compared with WT MCF7 cells (Supplementary Figure 6A).

Treatment with 10 nM OHT or FAS inhibited expression of these genes; nevertheless, expression of most genes was higher than that in anti-estrogen-treated WT cells. Indeed, whereas expression of PGR, TFF1 and CTSD was inhibited by as little as 1 nM OHT, levels of these proteins remained high even in the presence of 1 μM OHT (Supplementary Figure 6B). ER target gene expression was more responsive to FAS, but as for OHT, levels of all proteins were higher in the FAS-treated MCF7-Y537S cells than in parental MCF7 cells (Supplementary Figure 6C).

Finally, our results show that ER mRNA levels were similar in MCF7-WT and MCF7-Y537S cells (Figure 4d). Previous studies have shown that ER protein levels are reduced upon treatment of MCF7 cells with estrogen, OHT stabilizes ER and FAS promotes ER degradation (for example, see Wittmann *et al.*^42^). In agreement, ER protein levels were elevated with OHT treatment and were reduced by FAS treatment in MCF7 cells (Figure 4e, Supplementary Figures 6B and C). ER levels showed similar ligand regulation in MCF7-Y537S cells, which suggests that ER binding to ligands and its stability are not substantially affected by the Y537S mutation.

### CDK7 directed Ser-118 phosphorylation is ligand-independent in MCF7-Y537S cells

**Figure 5:**
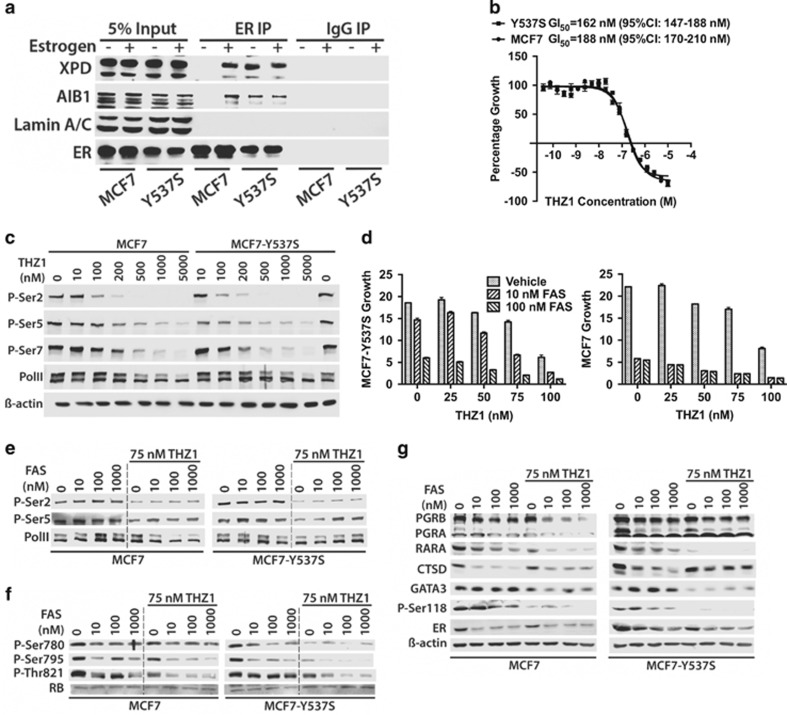
Inhibition of MCF7-Y537S growth by the CDK7 inhibitor THZ1 in combination with anti-estrogens. (**a**) TFIIH interacts in an estrogen-independent manner with ER in MCF7-Y537S cells. Protein lysates from hormone-depleted MCF7 and MCF7-Y537S cells treated with estrogen (10 nM) for 3 h were immunoprecipitated with an antibody for ER, or with mouse immunoglobulins (IgG; control). Immunoprecipitates were immunoblotted with antibodies for the XPD subunit of TFIIH, the ER co-activator AIB1 and a control protein (Lamin A/C). (**b**) Cells were treated with increasing concentrations of THZ1 for 48 h. Mean growth is shown relative to that for vehicle (dimethylsulphoxide-treated cells) (*n*=6). GI_50_=concentration of THZ1 at which cell growth is inhibited by 50%. (**c**) Protein lysates prepared from cells treated with increasing concentrations of THZ1 for 4 h were immunoblotted for PolII and for phosphorylation of Ser2, Ser5 and Ser7 in the PolII C-terminal domain heptapeptide repeat. (**d**) Cells were grown in DMEM containing 10% FCS over a 12-day period in the presence of 10 or 100 nM FAS, together with 20, 50, 75 or 100 nM THZ1. Growth was assessed using the SRB assay and is shown relative to SRB values at day 0. The bars represent mean values ±s.e.m. for six replicates. (**e**–**g**) Immunoblotting was performed with protein lysates prepared 24 h after addition of FAS±75 nM THZ1.

Estrogen binding promotes an interaction between the TFIIH complex that is mediated by the ER LBD and α-helical LXXLL motifs in the XPD and p62 subunits of TFIIH, resulting in Ser118 phosphorylation by the TFIIH kinase CDK7.^33^ TFIIH co-immunoprecipitated with ER in the presence, but not the absence of estrogen, as shown by immunoblotting for the XPD subunit of TFIIH (Figure 5a). In MCF7-Y537S cells, ER interacted with XPD in an estrogen-independent manner. Moreover, Ser118 phosphorylation was ligand-dependent in MCF7 cells, but was constitutive in MCF7-Y537S cells (Figure 4e).

THZ1 is a selective inhibitor of CDK7 that has demonstrable anti-tumour activities for a wide range of cancer types,^43^ acting to inhibit transcription by preventing phosphorylation of the RNA polymerase II (PolII) C-terminal domain heptapeptide repeats, and affecting cell cycle progression by inhibiting CDK7-mediated phosphorylation of CDK1, CDK2, CDK4 and CDK6.^44^ THZ1 inhibited MCF7 cell growth (GI_50_=188 nM) (Figure 5b). MCF7-Y537S cells were slightly more sensitive to THZ1 (GI_50_=162 nM), albeit with a difference that was not statistically significant. Inhibition of growth was accompanied by dose-dependent inhibition of PolII CTD phosphorylation (Figure 5c).

Reasoning that since (a) ER is a transcriptional driver in breast cancer cells, (b) CDK7 activity is required for transcription and (c) CDK7 phosphorylates ER at Ser118, we determined if co-treatment with THZ1 and anti-estrogens would provide additional growth inhibition. THZ1 augmented MCF7-Y537S inhibition by 10 nM FAS (Figure 5d). For 100 nM FAS, MCF7-Y537S growth was completely suppressed when combined with 75 nM THZ1. PolII phosphorylation was unaffected by FAS, being inhibited entirely by THZ1 (Figure 5e). RB phosphorylation was inhibited in a dose-dependent manner by FAS and was further inhibited by THZ1 co-treatment (Figure 5f). There was also a dose-dependent inhibition of Ser118 phosphorylation by FAS and strong inhibition by THZ1 (Figure 5g). Moreover, repression of ER target genes, PGR and RARA was greatest for the FAS/THZ1 combination. An important part of the response to THZ1 appears to derive from the special sensitivity of cancer cells to transcriptional inhibition of key driver genes, as exemplified in T-cell acute lymphoblastic leukaemia cells.^43^ RUNX1, TAL1 and GATA3, which together constitute a critical transcriptional circuit in T-cell acute lymphoblastic leukaemia cells, were among the genes that were most sensitive to THZ1. Interestingly, GATA3 was strongly inhibited by THZ1 in MCF7 and MCF7-Y537S cells. As a pioneer factor for ER in breast cancer cells,^45^ the potent inhibition of GATA3 expression by CDK7 inhibitors provides further evidence for its potential utility in the treatment of ER-positive breast cancer.

## Discussion

Mutations in the ER gene have recently been found to be common in advanced breast cancer and are likely to represent an important mechanism of endocrine resistance. Understanding the impact of these mutations on ER signalling in breast cancer cells is a crucial step towards identifying therapeutic interventions following emergence of ER mutations. Although tumour explants and patient-derived xenografts will be important for testing alternative therapies, modelling them in established, well-characterized breast cancer cell lines should facilitate rapid and intensive evaluation. By facilitating the generation of isogenic lines with mutations of interest encoded within endogenous genes, the application of CRISPR-Cas9 methodologies for gene replacement promises a powerful new tool for bypassing problems inherent in over-expression studies. With this in mind, we replaced tyrosine 537 in the endogenous ER gene of MCF7 estrogen responsive breast cancer cells, to make serine 537, one of the most common ER mutations in advanced, metastatic breast cancer.

Sequencing of genomic DNA, RNA-seq and quantitative mass-spectrometry demonstrated that MCF7-Y537S cells encode one copy of the mutant allele and express wild-type and Y537S ER proteins at equivalent levels, as is likely in metastatic breast cancer. This was sufficient to drive estrogen-independent growth, indicating that the Y537S mutation is dominant. Recently, Fanning *et al.*^19^ have reported detailed ligand binding properties of the Y537S LBD. Using a radioligand binding assay, they found that the affinity of E2 for the wild-type LBD is five-fold greater than that seen for the Y537S LBD. Further, while X-ray crystallography analysis of the apo- and agonist-bound states of the Y537S LBD show near identical helix 12 conformations, equating to a stable agonist state in the absence of E2, time-resolved Forster Resonance Energy Transfer (tr-FRET) measurements of co-activator binding show that E2 results in an increase in co-activator affinity in the Y537S LBD. Indeed, this behaviour is likely to be an important factor in both the E2 response of MCF7-Y537S cells, when comparing the expression of estrogen regulated genes between mutant and wild-type cells, and the mechanisms underlying the resistance to antiestrogens exhibited by MCF7-Y537S cells.

MCF7-Y537S cells were found to be resistant to both tamoxifen and the ER downregulator faslodex, although resistance to faslodex was less pronounced. In estrogen-free conditions, MCF7-Y537S cells were strongly inhibited by 1 and 10 nM FAS. However, if estrogen was present, potent growth inhibition required 100 nM FAS. It should be noted that peak (*C*_max_) plasma concentrations in patients following high dose administration of faslodex (500 mg), average at about 20 ng/ml (approximately 30 nM),^46, 47^ suggesting that only partial inhibition of ER signalling may be achievable with faslodex in patients with the Y537S mutation.

Using a competitive radioligand-binding assay to determine the relative binding affinities of OHT for WT and Y537S mutant LBDs, Fanning *et al*^19^ found that binding to the mutant LBD was 8-10-fold weaker than to the WT. Further, expression of Y537S mutated ER in the ER-positive T47D breast cancer cell line has shown that FAS can downregulate endogenous WT and transfected mutant ER protein, and could partially suppress growth of the mutant-expressing cells. Both the greatly reduced binding affinity of ER-Y537S to OHT, and the continued sensitivity of the mutant receptor to FAS, are consistent with the response that our MCF7-Y537S cells exhibit to anti-estrogens. It is possible that new selective ER modulators, such as the orally bioavailable AZD9496, GDC-0810 and RAD1901, could improve on patient responses, since they may not be subject to the dose limitations due to the poor pharmaceutical properties of faslodex.^48, 49, 50^ Additionally, we have now shown that it may be possible to achieve CDK7 inhibitor promoted growth inhibition using clinically achievable faslodex doses.

Of the group of ER LBD coding mutations featured in breast cancer, mutations of tyrosine 537 are among the most prevalent, with the Y537S mutation being the most frequent of these. It is well established that tyrosine 537 is the major tyrosine phosphorylation site in ER, where it is involved in receptor dimerization and DNA binding.^51, 52^ Furthermore, it has been shown that the Y537S mutated receptor also dimerizes effectively.^53^ Intriguingly, recent studies employing a chemical semi-synthesis strategy to prepare site specific *in vitro* phosphorylated ER LBDs have shown that tyrosine 537 phosphorylation results in a ligand-independent increase in coactivator binding.^54^ The activity of the Y537S ER mutation in this context similarly needs to be studied in detail, so that a better understanding of this mutation in metastatic breast cancer can be achieved. Clearly, the genome engineered MCF7-Y537S line described in our study provides an appropriate cellular model in which the findings from such studies can be evaluated and, perhaps more importantly, could be further used to develop new therapeutic strategies for metastatic breast cancer featuring Y537S ER mutations.

Mapping global ER binding showed that the Y537S mutation promotes estrogen-independent ER recruitment. Furthermore, recruitment of co-activators including AIB1 and p300, as well as PolII, was increased in the absence of estrogen. ER, co-activator and PolII recruitment were stimulated by estrogen, presumably due to co-expression with wild-type ER and/or estrogen stimulation of ER-Y537S activity. The majority (63%) of genes whose expression was stimulated by estrogen in MCF7 cells were upregulated in MCF7-Y537S cells in the absence of estrogen. Expression of these genes was stimulated further by estrogen. Estrogen treatment of MCF7 cells represses expression of as many, or more, genes than are stimulated.^38^ Integration of ER ChIP-seq and RNA-seq data identified 1559 estrogen-regulated genes in MCF7 cells, of which 52% were repressed by estrogen. Interestingly, the majority (65%) of these genes were repressed in MCF7-Y537S cells in the absence of estrogen and were further repressed by estrogen. These analyses indicate that the Y537S mutation does not reprogramme ER by causing genomic redistribution of ER or expression of new target genes, confirmed by the fact that GSEA analysis did not provide evidence for new signalling pathways.

The considerable over-expression, or ‘super-induction' of the majority of estrogen-induced genes, is extremely interesting and deserves further investigation. As expression of many transcription factors that co-operate with ER, such as RARA, PGR, LRH-1 and c-myc, as well as many transcriptional co-regulators, including AIB1, NRIP1, is stimulated by ER activation, estrogen-independent expression of these factors might be important for promoting chromatin remodelling and enhanced transcription at ER target genes. Gene expression at ER target genes requires the co-ordinated and cyclical recruitment and dissociation of ER and co-regulators at ER target genes.^28^ Ligand-independent recruitment of ER, and upregulation of ER-regulated transcription factors and co-regulators might alter the kinetics of chromatin remodelling, towards greater gene transcription. Also unexpected was the finding that the expression of many ER target genes was not ligand-independent, and indeed expression of many ER target genes, as evidenced by genes in clusters 2 and 3, was blunted in MCF7-Y537S cells. At present we can only speculate that these classes of genes involve co-operativity of ER with additional/alternative factors that are themselves estrogen regulated. Determination of ER and co-factor recruitment and chromatin modification/remodelling in time course studies of genes representative of the different categories, as well as proteomic approaches for identifying factors recruited to ER binding regions in MCF7 and MCF7-Y537S cells would help to more clearly define the mechanisms at work.

Notwithstanding, our results raise the possibility that tumours with ER mutations might be especially sensitive to drugs that target the activities of super-induced genes. Examples of strongly over-expressed genes identified by our analyses include RARA and PGR, for which agonists/antagonist drugs are available and both of which are key regulators of ER action in breast cancer.^55, 56, 57^ MCF7-Y537S cells provide an important vehicle for testing these and other therapeutic strategies, for example with genome-wide or targeted siRNA screening, or screening of drug libraries. Our results for the CDK7 inhibitor THZ1 exemplify such approaches for evaluation of new therapies that could be used singly, or in combination with endocrine therapies.

## Materials and methods

### Cell culture and growth assays

MCF7-luc and MCF7-Y537S cells were authenticated by LGC Standards (Bury, UK) as being MCF7 derived and were routinely checked, and found to be negative, for mycoplasma infection. Cell lines were routinely cultured in Dulbecco's modified Eagle's medium (DMEM) containing 10% fetal calf serum (FCS). For estrogen depletion experiments, the cells were transferred to DMEM lacking phenol red and containing 5% dextran-coated charcoal-stripped FCS (DSS) for 72 h. 17ß-estradiol (E2), 4-hydroxytamoxifen (OHT) and Faslodex (FAS) (Sigma-Aldrich, Dorset, UK) were prepared in ethanol. THZ1 (ApexBio, Houston, TX, USA) was dissolved in dimethylsulphoxide. Cell growth was assessed using the sulphorhodamine B (SRB) assay.^58^ Briefly, 4000 cells per well were seeded as six well technical replicates in 96-well plates in DMEM supplemented with 10% DSS. Sixteen hours later, the medium was replaced with fresh medium supplemented with E2, anti-estrogens, or an equivalent volume of the vehicle (ethanol). Medium was changed every 3 days and growth statistically analysed, so as to show average growth, with error bars for the standard error of the mean (s.e.m.). Each growth experiment was independently confirmed using three biological replicates.

### CRISPR-Cas9-mediated generation of the MCF7-Y537S cell line

The ESR1 Y537S mutation was generated in MCF7-luc cells (Cell Biolabs Inc, San Diego, CA, USA) using CRISPR058819 (5′-GGCTAGTGGGCGCATGTAGG-3′), identified from the Human exon specific CRISPR database (http://arep.med.harvard.edu/human_crispr) and cloned into a guide-RNA expression plasmid (a gift from George Church; Addgene #41824), as described.^35^ For homologous recombination, a 1803 bp fragment of the ESR1 gene flanking the Exon 8 coding region was amplified using the primers 5′-GGAAGAGCTTGGAGACATGG-3′ and 5′-AGGGCTAAATGCAACACCAG-3′, from MCF7-luc genomic DNA and cloned into pJET1.2/blunt (Thermo Scientific, Loughborough, UK). This ESR1 gene targeting template was modified by site-directed mutagenesis to incorporate a coding change for the Y537S mutation (TAT>TCT) and a silent ‘tagging' change at the codon for residue L536 (CTG>CTC). Site-directed mutagenesis generated additional silent mutations spanning the codons for residues 547-550, to prevent CRISPR058819-Cas9-mediated cleavage of the donor DNA. These changes also enable specific detection of Exon 8 targeted DNA by PCR with the mutation specific primer 5′-TAGTGGGCGCGTGAAGTCTA-3′ and a second primer, 5′-AAAATCAGTGTGGCTCCGGA-3′, so as to generate a 712 bp knock-in-specific PCR product, originating in genomic DNA 42nt upstream of the 5′ end of the donor DNA.

ESR1 Exon 8 gene targeting plasmid, CRISPR058819 expression plasmid and the hCas9 expression plasmid (a gift from George Church; Addgene plasmid #41815) were co-transfected into MCF7-luc cells using an Amaxa Type II nucleofector (Lonza, Cologne, Germany). Following nucleofection, cells were allowed to grow in hormone-depleted culture medium, and established colonies subsequently expanded in DMEM supplemented with 10% FCS. Colonies were screened with the knock-in-specific PCR and subsequently further characterized by DNA sequencing of a 462 bp PCR product generated using primers with the sequences 5′-CCAGCTCCCATCCTAAAGTG-3′ and 5′-TTGGCTAAAGTGGTGCATGA-3′, which amplify the coding region of Exon 8.

### Immunoblotting and immunoprecipitations

Whole cell lysates were prepared in RIPA buffer (Sigma-Aldrich), supplemented with protease and phosphatase inhibitor cocktails (Roche, West Sussex, UK), as described.^59^ Immunoblotting was performed for two biological replicates, using 20 μg protein lysate. Immunoprecipitations for two biological replicates were carried out as described.^60^ Antibodies are detailed in Supplementary Table 1.

### Liquid chromatography Mass Spectrometry

Following immunoprecipitation of ER from MCF7 and MCF7-Y537S cells, ER on the beads was incubated overnight at 37 °C with trypsin in 1 M urea, in a total volume of 20 μl. After addition of 5 μl of 20% formic acid, spectra were generated by collision-induced dissociation of tryptic digests in the linear ion trap of the LTQ Orbitrap Velos instrument.

### RNA preparation, quantitative RT-PCR and RNA-seq

Total RNA from three biological replicate cultures for each condition was extracted as previously described,^61^ with DNase treatment to remove genomic DNA. Quantitative RT-PCR (RT-qPCR) was carried out using three technical replicates for each sample for Taqman Gene Expression Assays (Applied Biosystems, Waltham, MA, USA), as listed in Supplementary Table 1 and presented as mean fold difference to the control, with error bars for s.e.m. RNA-seq was performed as described.^40^

### Chromatin immunoprecipitations and Solexa sequencing (ChIP-seq)

MCF7 and Y537S cells were cultured in hormone-depleted medium for 72 h. Chromatin was prepared from two biological replicates 45 min after addition of 10 nM estrogen and ChIP assays were performed as described^60^ using three technical replicates and the results compared using the unpaired *t*-test method. ChIP-seq was carried out on a single replicate for each treatment, using methods that have been described.^60^

### Bioinformatic analyses

ChIP-seq analysis was as described.^60^ RNA-seq data were analysed as described.^40^ In brief, paired-end 100 bp reads, generated on an Illumina Hiseq 2500, were aligned using TopHat 2.0.14 and aligned reads were counted by HTSeq 0.6.1. The R package ‘DESeq2' was used to normalize counts using a regularized log transformation (rlog).^62^ Shrunken log2 fold changes were also calculated to determine differentially expressed genes between conditions, while minimizing the fold change variation of low expression genes. Heat maps were generated in R using ‘gplots' with rlog transformed read counts of differentially expressed genes. GSEA analysis was performed with the Molecular Signatures Database ‘Hallmarks' gene set collection.^63^

The Binding and Expression Target Analysis package^37^ was used for integrating Chip-seq and RNA-seq data. To identify direct ER target genes, the Binding and Expression Target Analysis basic was run with a modified setting (look for peak within 100 kb from gene transcription start site).

RNA-seq and ChIP-seq data have been deposited with the NCBI Gene Expression Omnibus (GEO) (http://ncbi.nlm.nih.gov/geo/) under accession number GSE78286.
