# Supplementary material for: Genomic modelling of the ESR1 Y537S mutation for evaluating function and new therapeutic approaches for metastatic breast cancer
Source: Oncogene. 2016 Oct 17;36(16):2286–96. doi: 10.1038/onc.2016.382 (PMC5245767; doi:10.1038/onc.2016.382)
Supplement: Supplementary Legends [file onc2016382x1.docx]

**Supplementary Tables**

**Supplementary Table 1.**

Details of antibodies and PCR primers used in this study.

**Supplementary Table 2.**

Output from motif enrichment analysis of ER ChIP-seq called peaks using HOMER.

**Supplementary Table 3.**

Gene Set Enrichment Analysis (GSEA) output for differentially regulated genes (padj<0.05).

**Supplementary Table 4.**

Genes in clusters 1-4 in Figure 4C are listed, together with normalized read counts for each replicate sample and the mean expression difference, relative to vehicle-treated MCF7 cells.

**Supplementary Figure Legends**

**Supplementary Figure 1. Testing CRISPR sequences for indel activities in HCT116 and MCF7 cells.** (A) CRISPR guide RNA sequences and genomic locations are shown, with the CRISPR number identifiers from the CRISPR resource ((<http://arep.med.harvard.edu/human_crispr>). (B, C) HCT116 (B) or MCF7 (C) cells were transfected with the CRISPR plasmids alone (control), or together with a Cas9 expression plasmid that also encodes GFP, using Lipofectamine LTX. Transfection efficiencies were similar in a given cell line, ranging between 37-42%, as judged by scoring for GFP positivity. The Sanger sequencing chromatograms for each transfection are shown. Indels are indicated by the presence of mixed sequences and indicate that CRISPR058819 and CRISPR058822 were most efficient at generating indels in both cell lines. As CRISPR058819 maps closer to the Y537 codon than CRISPR058822, it was chosen for generating the MCF7-Y537 line.

**Supplementary Figure 2.** (A) Sequence of the ESR1 gene exon 8 coding region, showing the position of the Y537S codon (red) and the guide RNA (green). (B) Sequence of the ESR1 donor template encoding the Y537S (TAT>TcT) mutation. A silent change was introduced in the L536 codon (CTC>CTg). Silent changes that prevent CRISPR-Cas9 genome editing, were introduced in codons 547-550. (C) DNA sequencing chromatogram of genomic DNA for the MCF7-Y537S [NF2 A4] line. (D) Representative MS/MS spectra of wild-type (left) and Y537S mutant (right). The MAXQuant PEP values are indicated and for the wild-type peptide a Mascot search engine Expect value is also shown. The spectra were generated by collision-induced dissociation of tryptic digests in the linear ion trap of the LTQ/Orbitrap Velos instrument.

**Supplementary Figure 3.** Details of ER ChIP-sequencing runs.

**Supplementary Figure 4. Expression profiles of ER target genes in MCF7 and Y537S cells.** (A) Cells were cultured in hormone depleted medium for 72 hours. Estrogen (1 nM), or an equal volume of ethanol (vehicle) were added. RNA was prepared 4, 8, 16 and 24 hours following estrogen addition. Gene expression is shown relative to MCF7 cells treated with vehicle for 4 hours (n=3). (B) Comparison of RNA-seq shows that 4,873 genes are differentially expressed with estrogen treatment in MCF7 cells (padj<0.05). In MCF7-Y537S cells, 1,720 genes are estrogen-regulated in MCF7-Y537S, most of which (82%; 1418/1720) are also estrogen-regulated in MCF7 cells. (C) Heat map of cluster analysis for differentially regulated genes.

**Supplementary Figure 5. Gene expression profiles of MCF7-Y537S cells with gene expression profiles for MCF7 cells following prolonged estrogen exposure.** (A) Comparison of differentially regulated genes identified from RNA-seq (padj<0.05) for MCF7 and MCF7-Y537S cells treated with estrogen (8 hours), with expression profiles for MCF7 cells cultured long-term in DMEM supplemented with 10% FCS and 10 nM estrogen (<http://www.ncbi.nlm.nih.gov/geo/query/acc.cgi?acc=GSE60517>). (B) GSEA analysis for enriched signaling pathways in the gene set common to all three pair-wise comparisons in A. (C) GSEA analysis for genes that are common to vehicle-treated Y537S and MCF7 cells cultured in full medium. (D) Comparison of differentially regulated genes as in (A), using GEO RNA-seq data set GSE51403 (<http://www.ncbi.nlm.nih.gov/geo/query/acc.cgi?acc=GSE51403>), for MCF7 cells cultured in estrogen-depleted medium and treated with estrogen for 24 hours. (E, F) GSEA analysis of the analysis summarised in (D) for genes common to all three data sets, or for the genes that are differentially regulated in Y537S and MCF7 cells treated with estrogen for 24 hours.

**Supplementary Figure 6. Expression profiles of ER target genes in MCF7 and Y537S cells.** (A) RNA prepared from cells cultured in DMEM containing 10%FCS was used for RT-qPCR (n=3) Error bars show SEM. (B, C) immunoblotting of protein lysates prepared from cells cultured in DMEM+10%FCS following addition of OHT or FAS for 24 hours.
