# Supplementary material for: Genomic modelling of the ESR1 Y537S mutation for evaluating function and new therapeutic approaches for metastatic breast cancer
Source: Oncogene. 2016 Oct 17;36(16):2286–96. doi: 10.1038/onc.2016.382 (PMC5245767; doi:10.1038/onc.2016.382)
Supplement: Supplementary Figures [file onc2016382x2.pdf]

A

CRISPR Number	Chromosomal Location	Strand	Sequence
human_exon_crispr_v1_058819	chr6:152419954-152419976	-	GGCTAGTGGGCGCATGTAGGCGG
human_exon_crispr_v1_058820	chr6:152419967-152419989	-	GATGCCCCTCCACGGCTAGTGGG
human_exon_crispr_v1_058821	chr6:152419968-152419990	-	GGATGCCCCTCCACGGCTAGTGG
human_exon_crispr_v1_058822	chr6:152420050-152420072	+	GCAAAAGTATTACATCACGGGGG

B

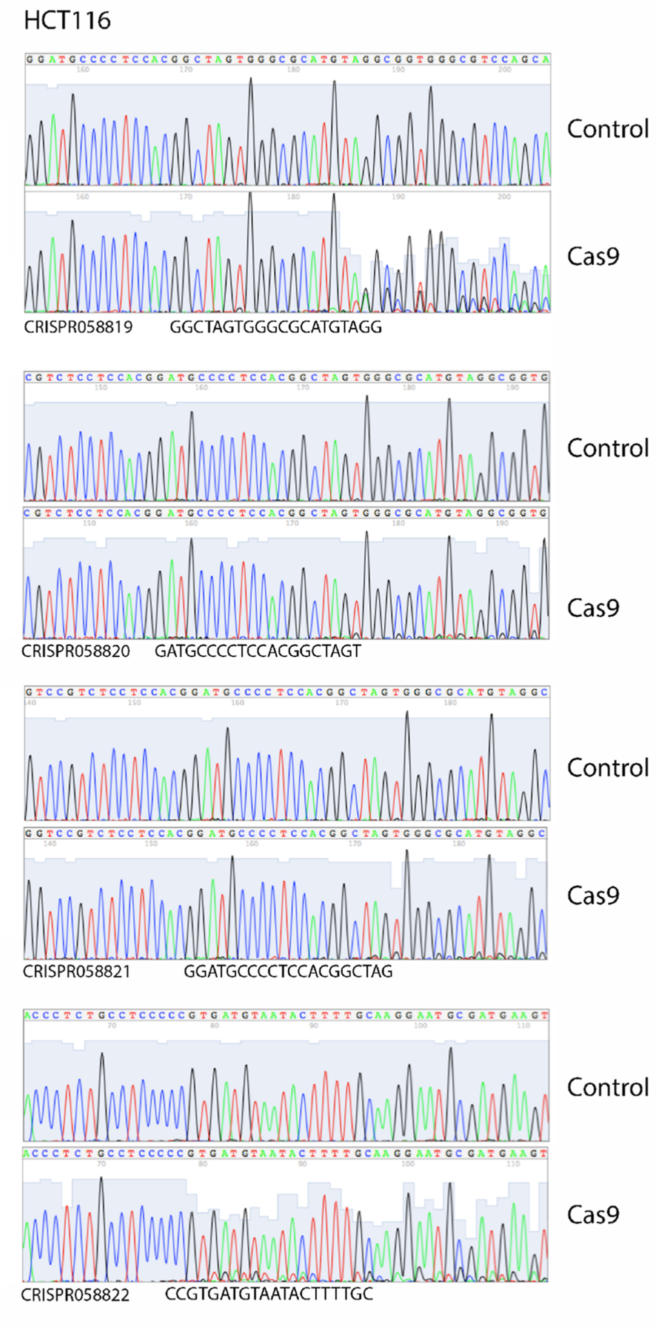

C

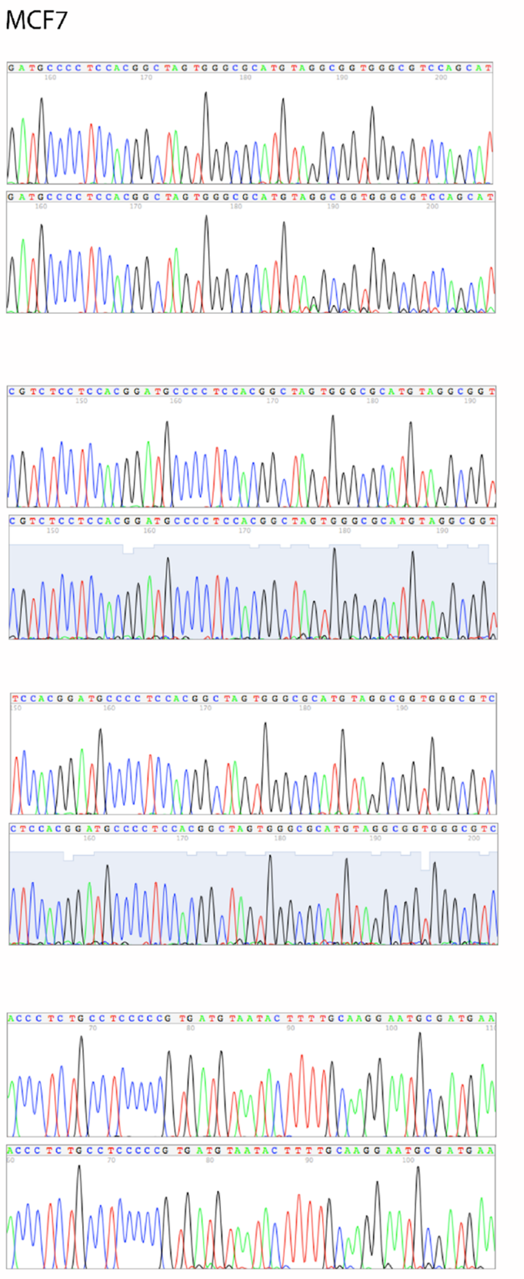

Supplementary Figure 1.

A ESR1 Exon 8 WT

```
AAC AAA GGC ATG GAG CAT CTG TAC AGC ATG AAG TGC AAG AAC GTG GTG CCC CTC TAT GAC CTG CTG CTG GAG ATG CTG GAC
N   K   G   M   E   H   L   Y   S   M   K   C   K   N   V   V   P   L   Y   D   L   L   L   E   M   L   D

GCC CAC CGC CTA CAT GCG CCC ACT AGC CGT GGA GGG GCA TCC GTG GAG GAG ACG GAC CAA AGC CAC TTG GCC ACT GCG GGC
A   H   R   L   H   A   P   T   S   R   G   G   A   S   V   E   E   T   D   Q   S   H   L   A   T   A   G

TCT ACT TCA TCG CAT TCC TTG CAA AAG TAT TAC ATC ACG GGG GAG GCA GAG GGT TTC CCT GCC ACG GTC TGA
S   T   S   S   H   S   L   Q   K   Y   Y   I   T   G   E   A   E   G   F   P   A   T   V   *
```

B ESR1 Exon 8 - Y537S

```
AAC AAA GGC ATG GAG CAT CTG TAC AGC ATG AAG TGC AAG AAC GTG GTG CCC CTg TcT GAC CTG CTG CTG GAG ATG CTG GAC
N   K   G   M   E   H   L   Y   S   M   K   C   K   N   V   V   P   L   S   D   L   L   L   E   M   L   D

GCC CAt aGa CTt CAc GCG CCC ACT AGC CGT GGA GGG GCA TCC GTG GAG GAG ACG GAC CAA AGC CAC TTG GCC ACT GCG GGC
A   H   R   L   H   A   P   T   S   R   G   G   A   S   V   E   E   T   D   Q   S   H   L   A   T   A   G

TCT ACT TCA TCG CAT TCC TTG CAA AAG TAT TAC ATC ACG GGG GAG GCA GAG GGT TTC CCT GCC ACG GTC TGA
S   T   S   S   H   S   L   Q   K   Y   Y   I   T   G   E   A   E   G   F   P   A   T   V   *
```

C

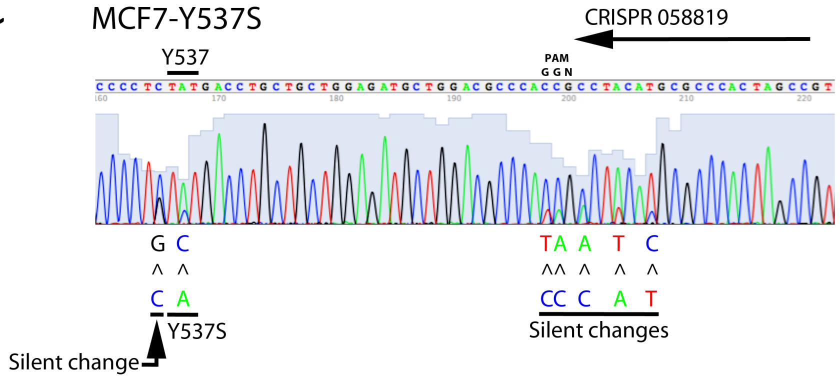

D

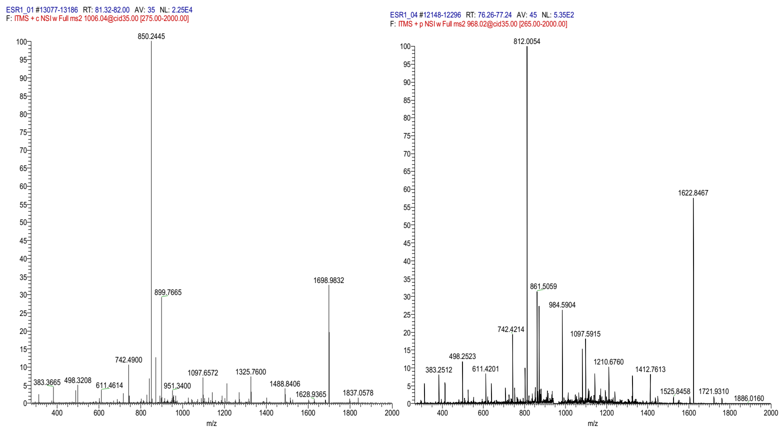

Supplementary Figure 2.

	Cell Line	Aligned Reads	% Aligned
**Input**	MCF7	39,546,235	97.1
	Y537S	29,153,768	97.3
**ER ChIP-seq**	MCF7 Vehicle	20,088,536	96.5
	MCF7 Estrogen	22,891,010	96.5
	Y537S Vehicle	22,794,741	96.6
	Y537S Estrogen	30,810,824	97.3

Supplementary Figure 3.

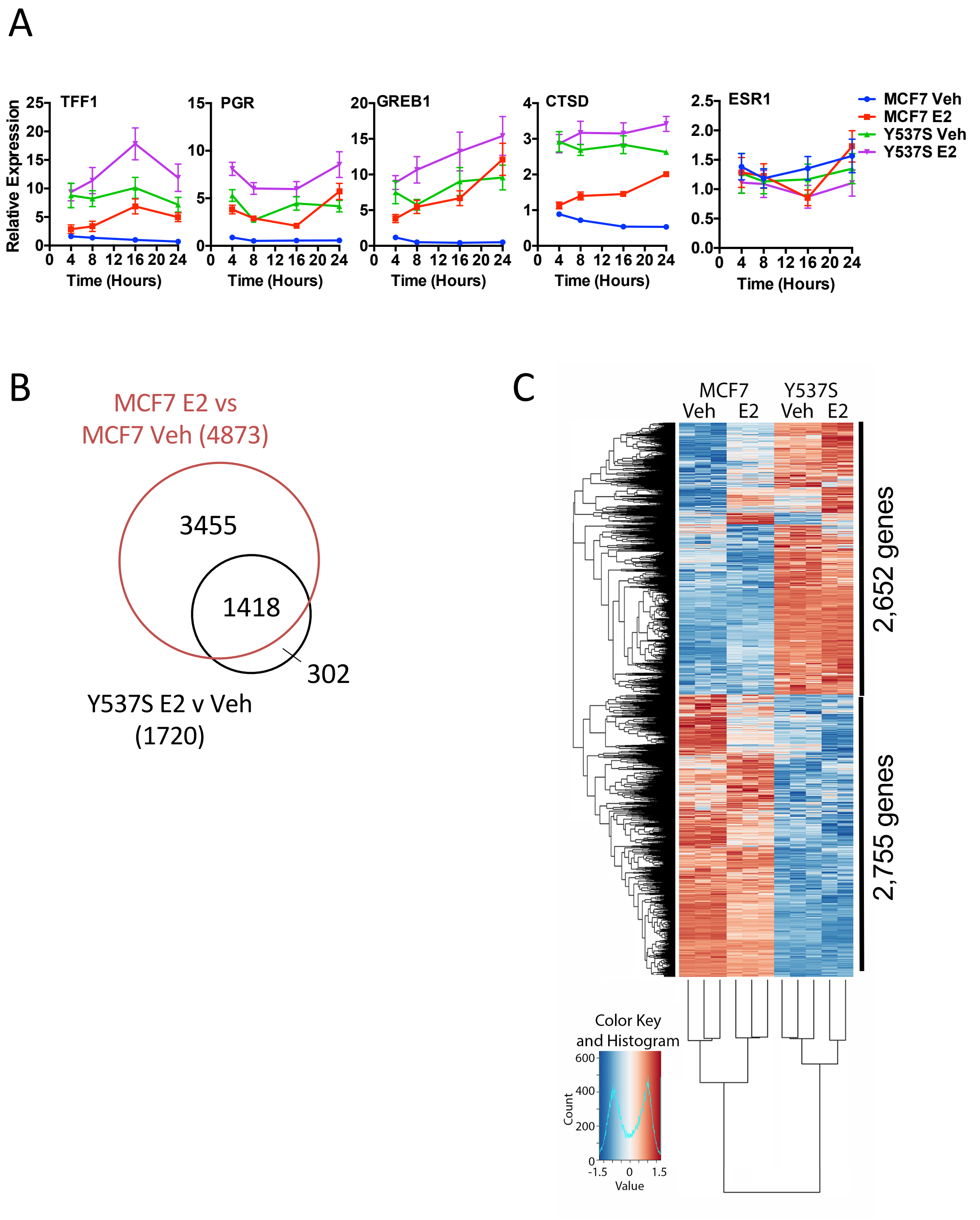

Supplementary Figure 4.

A

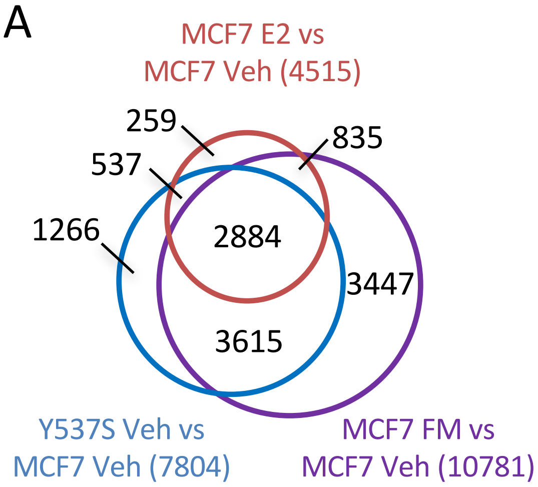

D

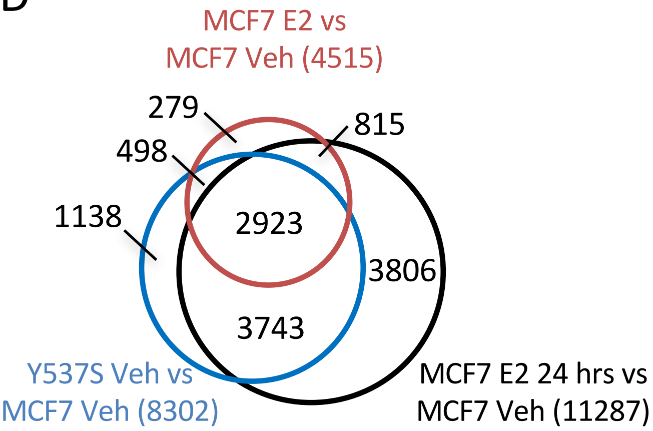

B

GSEA Analysis for Genes common to all MCF7 E2vsVeh, MCF7 Full medium v MCF7 Veh and Y537S veh vs MCF7 Veh

Pathway	# Genes in Gene Set (K)	# Genes in Overlap (k)	k/K	p-value	FDR q-value
HALLMARK_ESTROGEN_RESPONSE_EARLY	200	110	0.55	1.39E-79	6.96E-78
HALLMARK_E2F_TARGETS	200	109	0.545	2.73E-78	6.82E-77
HALLMARK_ESTROGEN_RESPONSE_LATE	200	98	0.49	1.16E-64	1.93E-63
HALLMARK_G2M_CHECKPOINT	200	89	0.445	2.64E-54	2.20E-53
HALLMARK_MITOTIC_SPINDLE	200	89	0.445	2.64E-54	2.20E-53
HALLMARK_TNFA_SIGNALING_VIA_NFKB	200	89	0.445	2.64E-54	2.20E-53
HALLMARK_MYC_TARGETS_V1	200	83	0.415	8.45E-48	6.04E-47
HALLMARK_MTORC1_SIGNALING	200	76	0.38	1.27E-40	7.05E-40
HALLMARK_P53_PATHWAY	200	76	0.38	1.27E-40	7.05E-40
HALLMARK_EPITHELIAL_MESENCHYMAL_TRANSITION	200	71	0.355	8.94E-36	4.47E-35

E

GSEA Analysis for Genes common to all MCF7 E2vsVeh, MCF7 E2 24 hrs v MCF7 Veh and Y537S veh vs MCF7 V

Pathway	# Genes in Gene Set (K)	# Genes in Overlap (k)	k/K	p-value
HALLMARK_E2F_TARGETS	200	75	0.375	3.77E-62
HALLMARK_ESTROGEN_RESPONSE_EARLY	200	66	0.33	1.35E-50
HALLMARK_ESTROGEN_RESPONSE_LATE	200	66	0.33	1.35E-50
HALLMARK_G2M_CHECKPOINT	200	59	0.295	3.58E-42
HALLMARK_TNFA_SIGNALING_VIA_NFKB	200	55	0.275	1.36E-37
HALLMARK_MYC_TARGETS_V1	200	48	0.24	5.20E-30
HALLMARK_EPITHELIAL_MESENCHYMAL_TRANSITION	200	47	0.235	5.64E-29
HALLMARK_HYPOXIA	200	47	0.235	5.64E-29
HALLMARK_MITOTIC_SPINDLE	200	43	0.215	5.90E-25
HALLMARK_P53_PATHWAY	200	43	0.215	5.90E-25

C

GSEA Analysis for Genes for Y537S vehicle vs MCF7 vehicle compared with MCF7 Full medium vs MCF7 vehicle

Pathway	# Genes in Gene Set (K)	# Genes in Overlap (k)	k/K	p-value	FDR q-value
HALLMARK_FATTY_ACID_METABOLISM	158	49	0.3101	3.30E-22	1.65E-20
HALLMARK_UV_RESPONSE_DN	144	43	0.2986	5.71E-19	1.12E-17
HALLMARK_OXIDATIVE_PHOSPHORYLATION	200	51	0.255	6.69E-19	1.12E-17
HALLMARK_INTERFERON_GAMMA_RESPONSE	200	49	0.245	1.91E-17	2.39E-16
HALLMARK_ADIPOGENESIS	200	47	0.235	4.92E-16	4.92E-15
HALLMARK_G2M_CHECKPOINT	200	46	0.23	2.39E-15	1.49E-14
HALLMARK_MITOTIC_SPINDLE	200	46	0.23	2.39E-15	1.49E-14
HALLMARK_P53_PATHWAY	200	46	0.23	2.39E-15	1.49E-14
HALLMARK_GLYCOLYSIS	200	45	0.225	1.13E-14	6.28E-14
HALLMARK_PEROXISOME	104	31	0.2981	4.60E-14	2.30E-13

F

GSEA Analysis for Genes common to all MCF7 E2 24 hrs v MCF7 Veh and Y537S veh vs MCF7 Veh

Pathway	# Genes in Gene Set (K)	# Genes in Overlap (k)	k/K	p-value
HALLMARK_MITOTIC_SPINDLE	200	74	0.37	2.40E-37
HALLMARK_E2F_TARGETS	200	73	0.365	2.13E-36
HALLMARK_G2M_CHECKPOINT	200	70	0.35	1.31E-33
HALLMARK_GLYCOLYSIS	200	57	0.285	1.49E-22
HALLMARK_FATTY_ACID_METABOLISM	158	48	0.3038	1.63E-20
HALLMARK_EPITHELIAL_MESENCHYMAL_TRANSITION	200	52	0.26	9.05E-19
HALLMARK_MYOGENESIS	200	52	0.26	9.05E-19
HALLMARK_MTORC1_SIGNALING	200	51	0.255	4.79E-18
HALLMARK_APOPTOSIS	161	44	0.2733	5.43E-17
HALLMARK_PI3K_AKT_MTOR_SIGNALING	105	35	0.3333	9.01E-17

Supplementary Figure 5.

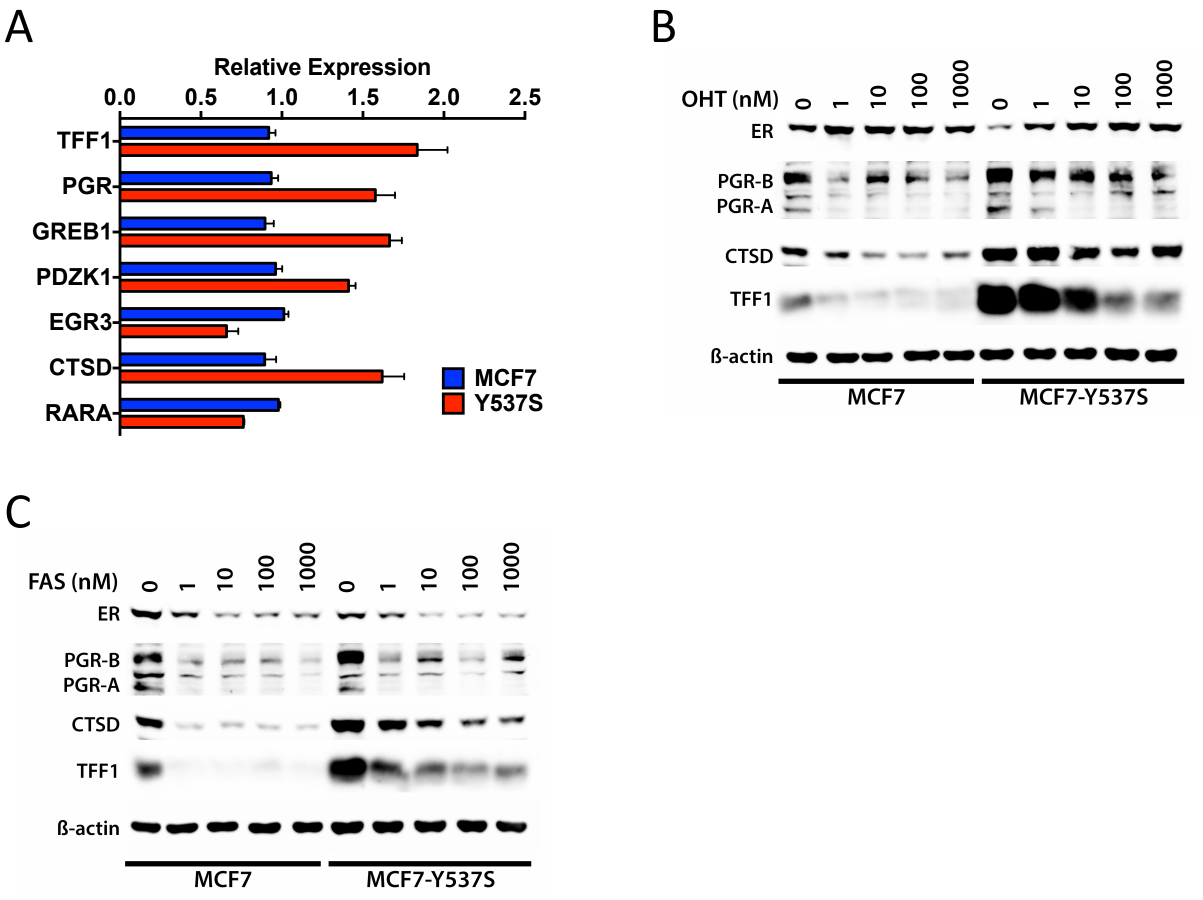

Supplementary Figure 6.
