# Supplementary material for: Genomic modelling of the ESR1 Y537S mutation for evaluating function and new therapeutic approaches for metastatic breast cancer
Source: Oncogene. 2016 Oct 17;36(16):2286–96. doi: 10.1038/onc.2016.382 (PMC5245767; doi:10.1038/onc.2016.382)
Supplement: Supplementary Table 1 [file onc2016382x3.pdf]

Antibodies for ChIP

Protein	Catalogue Number	Supplier
ESR1	sc-543	Santa Cruz
GATA3	sc-268	Santa Cruz
p300	sc-585	Santa Cruz
RNA polymerase II (PolII)	05-623	Millipore
Ac-H3	06-599	Millipore
AIB1	611105	BD Biosciences
FOXA1	ab5089, ab23738	Abcam
Normal Rabbit IgG	#2729	Cell Signalling

Antibodies for Immunoblotting

Protein	Dilution	Cat. No.	Supplier
ER	1 : 1,000	NCL-ER-6F11	Novacastra
P-Ser118	1:500	2511	Cell Signalling
P-Ser167	1:500	5587	Cell Signaling
FOXA1	1:1000	ab23738	Abcam
GATA3	1:500	sc-268x	Santa Cruz
PGR	1:200	sc-538	Santa Cruz
CTSD	1: 2,000	ab6313	Abcam
MYC	1 : 1,000	ab32	Abcam
TFF1	1:200	sc-28925	Santa Cruz
PDZK1	1:300	10507-2-AP	Proteintech
RARA	1 : 1,000	Ab39971	Abcam
CCND1	1:200	Ab16663	Abcam
ß-actin	1 : 100,000	Ab6276	Abcam
PolII	1:500	Ab5408	Abcam

PolII P-Ser2	1:500	Ab5095	Abcam
PolII P-Ser5	1:500	Ab5131	Abcam
PolII P-Ser7	1:500	Ab126538	Abcam
RB	1:500	sc-50	Santa Cruz
RB P-Ser780	1:500	9307	Cell Signaling
RB P-Ser795	1:500	9301	Cell Signaling
RB P-Ser807/811	1:500	9308	Cell Signaling
RB P-Thr821	1:500	Ab4787	Abcam
XPD	1:500	Ab111596	Abcam
CDK7	1 : 1,000	Ab9516-500	Abcam
LAMIN A/C	1:500	sc-7292	Santa Cruz

RT-qPCR Assays

Gene Name	Taqman Assay Cat. No.
ESR1	Hs00174860_m1
TFF1	Hs00170216_m1
GREB1	Hs00536409_m1
PGR	Hs00172183_m1
Cathepsin D (CTSD)	Hs00157201_m1
EGR3	Hs00231780_m1
PDZK1	Hs00275727_m1
MYC	Hs00153408_m1
RARA	Hs00940446_m1
GAPDH	Hs99999905_m1

ChIP-qPCR Primers

**TFF1 Promoter Proximal ERE:**	5’-TATGAATCACTTCTGCAGTGAG-3’
	5’-GAGCGTTAGATAACATTTGCC-3’
**TFF1 Control [Control 1]:**	5'-GTGATTCTCCTGACTTAACC-3’
	5’-TGGCGCAGTGGCTCACGCTG-3’
**GREB1**	5’-GGGTGAAATGAAGTGGCATGTG-3’
	5’-GAACAAAACAGAGCAAGGCCAAA-3’
**PGR 3':**	5’-TGCCTCTCCCATGTCTCCAT-3’
	5’-TCCCAGAATGTCAGCCTCAT-3’
**PGR Control [Control 2]:**	5’-CCACCACATTTTGTCCTCTGC-3’
	5’-GGGAGGGAGGAATGGAACTC-3’
**PDZK1:**	5’-AGGCCCAGCAAAGACAAATG-3’
	5’-AAACCACAGGCTGAGGACTG-3’
**MYC:**	5’-AGGGTGAGGTCAAGCATTTG-3’
	5’-TGGCCTTGAACCCATACTTC-3’
**XBP1:**	5’-ATCAGGGCAGCCTTCAAGAC-3’
	5’-CAGGGGAACAAAAGCAGCAC-3’
