# Supplementary material for: Genomic modelling of the ESR1 Y537S mutation for evaluating function and new therapeutic approaches for metastatic breast cancer
Source: Oncogene. 2016 Oct 17;36(16):2286–96. doi: 10.1038/onc.2016.382 (PMC5245767; doi:10.1038/onc.2016.382)
Supplement: Supplementary Table 2 [file onc2016382x4.pdf]

Motif Name	Consensus	P-value	Log P-value	q-value (Benjamini)	# of Target Sequences with Motif(of 11002)	% of Target Sequences with Motif	# of Background Sequences with Motif(of 38320)	% of Background Sequences with Motif
ERE(NR),IR3/MCF7-ERa-ChIP-Seq(Unpublished)/Homer	VAGGTCACNSTGACC	1e-813	-1.87E+03	0	864	34.56%	847	1.85%
FOXA1(Forkhead)/LNCAP-FOXA1-ChIP-Seq(GSE27824)/Homer	WAAGTAAACA	1.00E-167	-3.85E+02	0	756	30.24%	4681.6	10.21%
FOXA1(Forkhead)/MCF7-FOXA1-ChIP-Seq(GSE26831)/Homer	WAAGTAAACA	1.00E-153	-3.54E+02	0	653	26.12%	3803.2	8.29%
Foxa2(Forkhead)/Liver-Foxa2-ChIP-Seq(GSE25694)/Homer	CYTGTTTACWYW	1.00E-146	-3.36E+02	0	545	21.80%	2824.9	6.16%
Fox:Ebox(Forkhead,bHLH)/Panc1-Foxa2-ChIP-Seq(GSE47459)/Homer	NNNVCTGWGYAAACASN	1.00E-137	-3.18E+02	0	569	22.76%	3199.1	6.98%
Esrrb(NR)/mES-Esrrb-ChIP-Seq(GSE11431)/Homer	KTGACCTTGA	1.00E-110	-2.54E+02	0	443	17.72%	2394.2	5.22%
GRHL2(CP2)/HBE-GRHL2-ChIP-Seq(GSE46194)/Homer	AAACYKGTTWDACMRGTTTB	1.00E-71	-1.64E+02	0	258	10.32%	1253.6	2.73%
Fosl2(bZIP)/3T3L1-Fosl2-ChIP-Seq(GSE56872)/Homer	NATGASTCABNN	1.00E-66	-1.53E+02	0	221	8.84%	993.7	2.17%
Jun-AP1(bZIP)/K562-cJun-ChIP-Seq(GSE31477)/Homer	GATGASTCATCN	1.00E-56	-1.31E+02	0	171	6.84%	697.4	1.52%
Fra1(bZIP)/BT549-Fra1-ChIP-Seq(GSE46166)/Homer	NNATGASTCATH	1.00E-54	-1.25E+02	0	288	11.52%	1855.1	4.04%
Erra(NR)/HepG2-Erra-ChIP-Seq(GSE31477)/Homer	CAAAGGTCAG	1.00E-52	-1.21E+02	0	779	31.16%	8439.4	18.40%
BATF(bZIP)/Th17-BATF-ChIP-Seq(GSE39756)/Homer	DATGASTCAT	1.00E-51	-1.19E+02	0	323	12.92%	2310.2	5.04%
Atf3(bZIP)/GBM-ATF3-ChIP-Seq(GSE33912)/Homer	DATGASTCATHN	1.00E-50	-1.15E+02	0	318	12.72%	2294.7	5.00%
AP-1(bZIP)/ThioMac-PU.1-ChIP-Seq(GSE21512)/Homer	VTGACTCATC	1.00E-49	-1.13E+02	0	350	14.00%	2693.6	5.87%
Reverb(NR),DR2/RAW-Reverba.biotin-ChIP-Seq(GSE45914)/Homer	GTRGGTCASTGGGTCA	1.00E-48	-1.12E+02	0	131	5.24%	480.4	1.05%
TEAD4(TEA)/Tropoblast-Tead4-ChIP-Seq(GSE37350)/Homer	CCWGGAATGY	1.00E-40	-9.43E+01	0	327	13.08%	2661.3	5.80%
GATA(Zf),IR4/iTreg-Gata3-ChIP-Seq(GSE20898)/Homer	NAGATWNBNATCTNN	1.00E-40	-9.25E+01	0	91	3.64%	274.7	0.60%
FOXP1(Forkhead)/H9-FOXP1-ChIP-Seq(GSE31006)/Homer	NYYTGTTTACHN	1.00E-37	-8.57E+01	0	217	8.68%	1477.2	3.22%
Gata4(Zf)/Heart-Gata4-ChIP-Seq(GSE35151)/Homer	NBWGATAAGR	1.00E-37	-8.55E+01	0	407	16.28%	3830.2	8.35%
Foxo1(Forkhead)/RAW-Foxo1-ChIP-Seq(Fan et al.)/Homer	CTGTTTAC	1.00E-37	-8.53E+01	0	619	24.76%	6850.6	14.94%
GATA3(Zf)/iTreg-Gata3-ChIP-Seq(GSE20898)/Homer	AGATAASR	1.00E-33	-7.76E+01	0	555	22.20%	6083.6	13.26%
Nur77(NR)/K562-NR4A1-ChIP-Seq(GSE31363)/Homer	TGACCTTTNCNT	1.00E-33	-7.67E+01	0	122	4.88%	595.3	1.30%
TEAD(TEA)/Fibroblast-PU.1-ChIP-Seq(Unpublished)/Homer	YCWGGAATGY	1.00E-32	-7.38E+01	0	270	10.80%	2246.4	4.90%
AP-2alpha(AP2)/Hela-AP2alpha-ChIP-Seq(GSE31477)/Homer	ATGCCCTGAGGC	1.00E-31	-7.32E+01	0	255	10.20%	2070.4	4.51%
Nr5a2(NR)/Pancreas-LRH1-ChIP-Seq(GSE34295)/Homer	BTCAAGGTCA	1.00E-30	-6.98E+01	0	277	11.08%	2392.9	5.22%
GATA(Zf),IR3/iTreg-Gata3-ChIP-Seq(GSE20898)/Homer	NNNNNBAGATAWYATCTVHN	1.00E-29	-6.73E+01	0	127	5.08%	709.2	1.55%
AP-2gamma(AP2)/MCF7-TFAP2C-ChIP-Seq(GSE21234)/Homer	SCCTSAGGSCAW	1.00E-27	-6.44E+01	0	293	11.72%	2687	5.86%
Bach2(bZIP)/OCILy7-Bach2-ChIP-Seq(GSE44420)/Homer	TGCTGAGTCA	1.00E-25	-5.93E+01	0	103	4.12%	541.4	1.18%
RORgt(NR)/EL4-RORgt.Flag-ChIP-Seq(GSE56019)/Homer	AAYTAGGTCA	1.00E-24	-5.57E+01	0	93	3.72%	474.8	1.04%
GATA3(Zf),DR8/iTreg-Gata3-ChIP-Seq(GSE20898)/Homer	AGATSTNDNNDSAGATAASN	1.00E-22	-5.27E+01	0	65	2.60%	254.5	0.56%
TEAD2(TEA)/Py2T-Tead2-ChIP-Seq(GSE55709)/Homer	CCWGGAATGY	1.00E-22	-5.17E+01	0	201	8.04%	1716.2	3.74%
Gata2(Zf)/K562-GATA2-ChIP-Seq(GSE18829)/Homer	BBCTTATCTS	1.00E-21	-4.99E+01	0	256	10.24%	2457.7	5.36%
NF1(CTF)/LNCAP-NF1-ChIP-Seq(Unpublished)/Homer	CYTGGCABNSTGCCAR	1.00E-17	-3.98E+01	0	135	5.40%	1082.1	2.36%
Gata1(Zf)/K562-GATA1-ChIP-Seq(GSE18829)/Homer	SAGATAAGRV	1.00E-16	-3.90E+01	0	216	8.64%	2133.7	4.65%
Atf2(bZIP)/3T3L1-Atf2-ChIP-Seq(GSE56872)/Homer	NRRTGACGTCAT	1.00E-14	-3.26E+01	0	140	5.60%	1253.8	2.73%
GATA3(Zf),DR4/iTreg-Gata3-ChIP-Seq(GSE20898)/Homer	AGATGKDGAGATAAG	1.00E-13	-3.21E+01	0	58	2.32%	320	0.70%

Nr5a2(NR)/mES-Nr5a2-ChIP-Seq(GSE19019)/Homer	BTCAAGGTCA	1.00E-13	-3.04E+01	0	173	6.92%	1727.8	3.77%
RXR(NR),DR1/3T3L1-RXR-ChIP-Seq(GSE13511)/Homer	TAGGGCAAAGGTCA	1.00E-13	-3.04E+01	0	289	11.56%	3383.4	7.38%
Bach1(bZIP)/K562-Bach1-ChIP-Seq(GSE31477)/Homer	AWWNTGCTGAGTCAT	1.00E-11	-2.61E+01	0	35	1.40%	153.1	0.33%
Smad3(MAD)/NPC-Smad3-ChIP-Seq(GSE36673)/Homer	TWGTCTGV	1.00E-10	-2.51E+01	0	727	29.08%	10675.7	23.28%
Tlx?(NR)/NPC-H3K4me1-ChIP-Seq(GSE16256)/Homer	CTGGCAGSCTGCCA	1.00E-10	-2.43E+01	0	121	4.84%	1156.9	2.52%
PR(NR)/T47D-PR-ChIP-Seq(GSE31130)/Homer	VAGRACAKNCTGTBC	1.00E-10	-2.36E+01	0	533	21.32%	7502.5	16.36%
NF-E2(bZIP)/K562-NFE2-ChIP-Seq(GSE31477)/Homer	GATGACTCAGCA	1.00E-10	-2.34E+01	0	33	1.32%	152.8	0.33%
EBF1(EBF)/Near-E2A-ChIP-Seq(GSE21512)/Homer	GTCCCCWGGGGA	1.00E-10	-2.33E+01	0	220	8.80%	2573.4	5.61%
ARE(NR)/LNCAP-AR-ChIP-Seq(GSE27824)/Homer	RGRACASNSTGTYCYB	1.00E-09	-2.28E+01	0	83	3.32%	697.4	1.52%
Atf1(bZIP)/K562-ATF1-ChIP-Seq(GSE31477)/Homer	GATGACGTCA	1.00E-09	-2.14E+01	0	260	10.40%	3237	7.06%
c-Jun-CRE(bZIP)/K562-cJun-ChIP-Seq(GSE31477)/Homer	ATGACGTCATCY	1.00E-09	-2.12E+01	0	126	5.04%	1286.3	2.80%
NF1:FOXA1(CTF,Forkhead)/LNCAP-FOXA1-ChIP-Seq(GSE27824)/Homer	WNTGTTTRYTTTGGCA	1.00E-09	-2.10E+01	0	29	1.16%	132.4	0.29%
Nrf2(bZIP)/Lymphoblast-Nrf2-ChIP-Seq(GSE37589)/Homer	HTGCTGAGTCAT	1.00E-08	-1.99E+01	0	29	1.16%	139.2	0.30%
PPARE(NR),DR1/3T3L1-Pparg-ChIP-Seq(GSE13511)/Homer	TGACCTTTGCCCCA	1.00E-08	-1.98E+01	0	237	9.48%	2942.8	6.42%
MafA(bZIP)/Islet-MafA-ChIP-Seq(GSE30298)/Homer	TGCTGACTCA	1.00E-08	-1.97E+01	0	213	8.52%	2581.2	5.63%
Atf7(bZIP)/3T3L1-Atf7-ChIP-Seq(GSE56872)/Homer	NGRTGACGTCAY	1.00E-07	-1.73E+01	0	188	7.52%	2285.2	4.98%
Smad2(MAD)/ES-SMAD2-ChIP-Seq(GSE29422)/Homer	CTGTCTGG	1.00E-06	-1.50E+01	0	380	15.20%	5430.2	11.84%
Cdx2(Homeobox)/mES-Cdx2-ChIP-Seq(GSE14586)/Homer	GYMATAAAAH	1.00E-06	-1.45E+01	0	207	8.28%	2679.9	5.84%
STAT5(Stat)/mCD4+-Stat5-ChIP-Seq(GSE12346)/Homer	RTTTCTNAGAAA	1.00E-06	-1.43E+01	0	84	3.36%	865.9	1.89%
MafK(bZIP)/C2C12-MafK-ChIP-Seq(GSE36030)/Homer	GCTGASTCAGCA	1.00E-06	-1.39E+01	0	63	2.52%	593.1	1.29%
Hoxc9(Homeobox)/Ainv15-Hoxc9-ChIP-Seq(GSE21812)/Homer	GGCCATAAATCA	1.00E-05	-1.38E+01	0	150	6.00%	1833.9	4.00%
SCL(bHLH)/HPC7-Scl-ChIP-Seq(GSE13511)/Homer	AVCAGCTG	1.00E-05	-1.37E+01	0	1019	40.76%	16588.1	36.17%
NF1-halfsite(CTF)/LNCaP-NF1-ChIP-Seq(Unpublished)/Homer	YTGCCAAG	1.00E-05	-1.34E+01	0	394	15.76%	5754.7	12.55%
HOXA9(Homeobox)/HSC-Hoxa9-ChIP-Seq(GSE33509)/Homer	GGCCATAAATCA	1.00E-05	-1.26E+01	0	188	7.52%	2458.3	5.36%
Sox10(HMG)/SciaticNerve-Sox3-ChIP-Seq(GSE35132)/Homer	CCWTTGTYYB	1.00E-05	-1.21E+01	0	400	16.00%	5937.2	12.95%
Bcl6(Zf)/Liver-Bcl6-ChIP-Seq(GSE31578)/Homer	NNNCTTTCCAGGAAA	1.00E-05	-1.20E+01	0	274	10.96%	3860.3	8.42%
Six1(Homeobox)/Myoblast-Six1-ChIP-Chip(GSE20150)/Homer	GKVTCADRTTWC	1.00E-04	-1.12E+01	0.0001	78	3.12%	855.2	1.86%
Unknown-ESC-element(?)/mES-Nanog-ChIP-Seq(GSE11724)/Homer	CACAGCAGGGGG	1.00E-04	-9.86E+00	0.0002	128	5.12%	1640.7	3.58%
RARg(NR)/ES-RARg-ChIP-Seq(GSE30538)/Homer	AGGTCAAGGTCA	1.00E-04	-9.37E+00	0.0003	18	0.72%	114	0.25%
STAT6(Stat)/Macrophage-Stat6-ChIP-Seq(GSE38377)/Homer	TTCCKNAGAA	1.00E-04	-9.28E+00	0.0004	121	4.84%	1555.2	3.39%
JunD(bZIP)/K562-JunD-ChIP-Seq/Homer	ATGACGTCATCN	1.00E-03	-9.12E+00	0.0004	32	1.28%	279.8	0.61%
Oct4:Sox17(POU,Homeobox,HMG)/F9-Sox17-ChIP-Seq(GSE44553)/Homer	CCATTGTATGCAAAT	1.00E-03	-8.96E+00	0.0005	45	1.80%	451.9	0.99%
Egr2(Zf)/Thymocytes-Egr2-ChIP-Seq(GSE34254)/Homer	NGCGTGGGCGGR	1.00E-03	-8.55E+00	0.0007	33	1.32%	301.8	0.66%
Egr1(Zf)/K562-Egr1-ChIP-Seq(GSE32465)/Homer	TGCGTGGGYG	1.00E-03	-8.45E+00	0.0008	110	4.40%	1418.7	3.09%
FXR(NR),IR1/Liver-FXR-ChIP-Seq(Chong et al.)/Homer	AGGTCANTGACCTB	1.00E-03	-8.39E+00	0.0008	100	4.00%	1267.9	2.76%
TR4(NR),DR1/Hela-TR4-ChIP-Seq(GSE24685)/Homer	GAGGTCAAAGGTCA	1.00E-03	-7.83E+00	0.0015	31	1.24%	288.3	0.63%
PRDM14(Zf)/H1-PRDM14-ChIP-Seq(GSE22767)/Homer	RGGTCTCTAACY	1.00E-03	-7.69E+00	0.0017	74	2.96%	899.2	1.96%
STAT1(Stat)/HelaS3-STAT1-ChIP-Seq(GSE12782)/Homer	NATTTCCNGGAAAT	1.00E-03	-7.50E+00	0.002	63	2.52%	742.3	1.62%
GFY-Staf(?,Zf)/Promoter/Homer	RACTACAATTCCCAGAAKGC	1.00E-03	-7.25E+00	0.0025	14	0.56%	92.9	0.20%
Pdx1(Homeobox)/Islet-Pdx1-ChIP-Seq(SRA008281)/Homer	YCATYAATCA	1.00E-03	-6.91E+00	0.0035	251	10.04%	3793.7	8.27%

Sox3(HMG)/NPC-Sox3-ChIP-Seq(GSE33059)/Homer	CCWTTGTY	1.00E-02	-6.77E+00	0.0039	403	16.12%	6397.2	13.95%
Rbpj1(?)/Panc1-Rbpj1-ChIP-Seq(GSE47459)/Homer	HTTTCCCASG	1.00E-02	-6.44E+00	0.0054	288	11.52%	4455.2	9.71%
Foxh1(Forkhead)/hESC-FOXH1-ChIP-Seq(GSE29422)/Homer	NNTGTGGATTSS	1.00E-02	-6.43E+00	0.0054	148	5.92%	2118.1	4.62%
PAX5(Paired,Homeobox),condensed/GM12878-PAX5-ChIP-Seq(GSE32465)/Homer	GTCACGCTCSCTGM	1.00E-02	-6.35E+00	0.0057	30	1.20%	304	0.66%
NFkB-p65-Rel(RHD)/ThioMac-LPS-Expression(GSE23622)/Homer	GGAAATTCCC	1.00E-02	-6.30E+00	0.006	17	0.68%	137.5	0.30%
STAT4(Stat)/CD4-Stat4-ChIP-Seq(GSE22104)/Homer	NYTTCCWGGAAR	1.00E-02	-5.93E+00	0.0086	189	7.56%	2826	6.16%
Olig2(bHLH)/Neuron-Olig2-ChIP-Seq(GSE30882)/Homer	RCCATMTGTT	1.00E-02	-5.87E+00	0.009	431	17.24%	6972.4	15.20%
E2F7(E2F)/Hela-E2F7-ChIP-Seq(GSE32673)/Homer	VDTTTCCCGCCA	1.00E-02	-5.79E+00	0.0096	19	0.76%	169.4	0.37%
Ap4(bHLH)/AML-Tfap4-ChIP-Seq(GSE45738)/Homer	NAHCAGCTGD	1.00E-02	-5.75E+00	0.0099	243	9.72%	3747.7	8.17%
Smad4(MAD)/ESC-SMAD4-ChIP-Seq(GSE29422)/Homer	VBSYGTCTGG	1.00E-02	-5.35E+00	0.0146	347	13.88%	5567.8	12.14%
GATA:SCL(Zf,bHLH)/Ter119-SCL-ChIP-Seq(GSE18720)/Homer	CRGCTGBNGNSNNSAGATAA	1.00E-02	-5.24E+00	0.0161	28	1.12%	300.8	0.66%
Unknown(Homeobox)/Limb-p300-ChIP-Seq/Homer	SSCMATWAAA	1.00E-02	-5.03E+00	0.0196	159	6.36%	2388.9	5.21%
ZFX(Zf)/mES-Zfx-ChIP-Seq(GSE11431)/Homer	AGGCCTRG	1.00E-02	-4.99E+00	0.0202	248	9.92%	3897.8	8.50%
EBF(EBF)/proBcell-EBF-ChIP-Seq(GSE21978)/Homer	DGTCCCYRGGGA	1.00E-02	-4.87E+00	0.0225	38	1.52%	455	0.99%
Atoh1(bHLH)/Cerebellum-Atoh1-ChIP-Seq(GSE22111)/Homer	VNRVCAGCTGGY	1.00E-02	-4.81E+00	0.0237	221	8.84%	3452.2	7.53%
Sox4(HMG)/proB-Sox4-ChIP-Seq(GSE50066)/Homer	YCTTTGTTCC	1.00E-02	-4.73E+00	0.0254	182	7.28%	2797.9	6.10%
HNF4a(NR),DR1/HepG2-HNF4a-ChIP-Seq(GSE25021)/Homer	CARRGKBCAAAGTYCA	1.00E-02	-4.63E+00	0.0276	99	3.96%	1423.9	3.10%
Tcfcp2l1(CP2)/mES-Tcfcp2l1-ChIP-Seq(GSE11431)/Homer	NRAACCRGTTYRAACCRGYT	1.00E-01	-4.59E+00	0.0284	28	1.12%	316.6	0.69%
BMYB(HTH)/Hela-BMYB-ChIP-Seq(GSE27030)/Homer	NHAACBGYYV	1.00E-01	-4.57E+00	0.0289	317	12.68%	5128.1	11.18%
HRE(HSF)/HepG2-HSF1-ChIP-Seq(GSE31477)/Homer	BSTTCTRGAABVTTCYAGAA	1.00E-01	-4.37E+00	0.0348	28	1.12%	322.6	0.70%
GRE(NR),IR3/RAW264.7-GRE-ChIP-Seq(Unpublished)/Homer	VAGRACAKWCTGTYC	1.00E-01	-4.25E+00	0.0388	51	2.04%	674.4	1.47%
KLF5(Zf)/LoVo-KLF5-ChIP-Seq(GSE49402)/Homer	DGGGYGKGGC	1.00E-01	-4.19E+00	0.0407	225	9.00%	3576.7	7.80%
SPDEF(ETS)/VCaP-SPDEF-ChIP-Seq(SRA014231)/Homer	ASWTCCTGBT	1.00E-01	-4.10E+00	0.0441	190	7.60%	2985.7	6.51%
Klf4(Zf)/mES-Klf4-ChIP-Seq(GSE11431)/Homer	GCCACACCCA	1.00E-01	-3.97E+00	0.0498	80	3.20%	1151.9	2.51%
Stat3+il21(Stat)/CD4-Stat3-ChIP-Seq(GSE19198)/Homer	SVYTTCCNGGAARB	1.00E-01	-3.80E+00	0.0587	122	4.88%	1858.3	4.05%
PAX3:FKHR-fusion(Paired,Homeobox)/Rh4-PAX3:FKHR-ChIP-Seq(GSE19063)/Homer	ACCRTGACTAATTNN	1.00E-01	-3.58E+00	0.0725	47	1.88%	640.8	1.40%
MITF(bHLH)/MastCells-MITF-ChIP-Seq(GSE48085)/Homer	RTCATGTGAC	1.00E-01	-3.49E+00	0.0785	207	8.28%	3336.8	7.28%
bZIP:IRF(bZIP,IRF)/Th17-BatF-ChIP-Seq(GSE39756)/Homer	NAGTTTCABTHTGACTNW	1.00E-01	-3.42E+00	0.0829	106	4.24%	1617.4	3.53%
MYB(HTH)/ERMYB-Myb-ChIPSeq(GSE22095)/Homer	GGCVGTTR	1.00E-01	-3.39E+00	0.0845	351	14.04%	5864.1	12.79%
CRE(bZIP)/Promoter/Homer	CSGTGACGTCAC	1.00E-01	-3.39E+00	0.0845	42	1.68%	569.6	1.24%
AMYB(HTH)/Testes-AMYB-ChIP-Seq(GSE44588)/Homer	TGGCAGTTGG	1.00E-01	-3.38E+00	0.0845	314	12.56%	5214.8	11.37%
EKLF(Zf)/Erythrocyte-Klf1-ChIP-Seq(GSE20478)/Homer	NWGGGTGTGGCY	1.00E-01	-3.19E+00	0.1007	48	1.92%	674.3	1.47%
NFkB-p65(RHD)/GM12787-p65-ChIP-Seq(GSE19485)/Homer	WGGGGATTTCCC	1.00E-01	-3.13E+00	0.1063	86	3.44%	1303.8	2.84%
RUNX(Runt)/HPC7-Runx1-ChIP-Seq(GSE22178)/Homer	SAAACCACAG	1.00E-01	-3.05E+00	0.1142	154	6.16%	2466.7	5.38%
Rfx1(HTH)/NPC-H3K4me1-ChIP-Seq(GSE16256)/Homer	KGTTGCCATGGCAA	1.00E-01	-2.87E+00	0.1354	35	1.40%	482	1.05%
p63(p53)/Keratinocyte-p63-ChIP-Seq(GSE17611)/Homer	NNDRCATGYCYNRRCATGYH	1.00E-01	-2.84E+00	0.1371	67	2.68%	1005.6	2.19%
Tcf12(bHLH)/GM12878-Tcf12-ChIP-Seq(GSE32465)/Homer	VCAGCTGYTG	1.00E-01	-2.77E+00	0.1458	174	6.96%	2840.9	6.19%
NeuroD1(bHLH)/Islet-NeuroD1-ChIP-Seq(GSE30298)/Homer	GCCATCTGTT	1.00E-01	-2.77E+00	0.1458	161	6.44%	2615.4	5.70%
Tcf4(HMG)/Hct116-Tcf4-ChIP-Seq(SRA012054)/Homer	ASATCAAAGGVA	1.00E-01	-2.64E+00	0.164	94	3.76%	1475.1	3.22%
GFY(?)/Promoter/Homer	ACTACAATTCCC	1.00E-01	-2.59E+00	0.1712	13	0.52%	151.1	0.33%

Meis1(Homeobox)/MastCells-Meis1-ChIP-Seq(GSE48085)/Homer	VGCTGWCAVB	1.00E-01	-2.58E+00	0.1712	405	16.20%	6946.3	15.15%
HOXD13(Homeobox)/Chicken-Hoxd13-ChIP-Seq(GSE38910)/Homer	NCYAATAAAA	1.00E-01	-2.58E+00	0.1712	249	9.96%	4178.7	9.11%
RFX(HTH)/K562-RFX3-ChIP-Seq(SRA012198)/Homer	CGGTTGCCATGGCAAC	1.00E-01	-2.56E+00	0.1712	12	0.48%	137.2	0.30%
MafF(bZIP)/HepG2-MafF-ChIP-Seq(GSE31477)/Homer	HWWGTCAGCAWWTTT	1.00E-01	-2.47E+00	0.1863	73	2.92%	1132.9	2.47%
X-box(HTH)/NPC-H3K4me1-ChIP-Seq(GSE16256)/Homer	GGTTGCCATGGCAA	1.00E-01	-2.43E+00	0.192	20	0.80%	262.2	0.57%
HIF-1a(bHLH)/MCF7-HIF1a-ChIP-Seq(GSE28352)/Homer	TACGTGCV	1.00E-01	-2.36E+00	0.205	45	1.80%	670.5	1.46%
Phox2a(Homeobox)/Neuron-Phox2a-ChIP-Seq(GSE31456)/Homer	YTAATYNRATTA	1.00E-01	-2.34E+00	0.2073	87	3.48%	1381.5	3.01%
Rfx2(HTH)/LoVo-RFX2-ChIP-Seq(GSE49402)/Homer	GTTGCCATGGCAACM	1.00E+00	-2.30E+00	0.214	14	0.56%	174.7	0.38%
E2F(E2F)/Hela-CellCycle-Expression/Homer	TTSGCGCGAAAA	1.00E+00	-2.28E+00	0.2172	7	0.28%	72.3	0.16%
NFAT(RHD)/Jurkat-NFATC1-ChIP-Seq(Jolma et al.)/Homer	ATTTTCCATT	1.00E+00	-2.26E+00	0.2198	166	6.64%	2761	6.02%
Stat3(Stat)/mES-Stat3-ChIP-Seq(GSE11431)/Homer	CTTCCGGGAA	1.00E+00	-2.05E+00	0.2676	79	3.16%	1269.8	2.77%
RUNX-AML(Runt)/CD4+-PolII-ChIP-Seq(Barski et al.)/Homer	GCTGTGGTTW	1.00E+00	-2.01E+00	0.2755	156	6.24%	2616.1	5.70%
Brachyury(T-box)/Mesoendoderm-Brachyury-ChIP-exo(GSE54963)/Homer	ANTTMRCASBNNNNGTGYKAAN	1.00E+00	-1.96E+00	0.2871	58	2.32%	916.3	2.00%
Ets1-distal(ETS)/CD4+-PolII-ChIP-Seq(Barski et al.)/Homer	MACAGGAAGT	1.00E+00	-1.96E+00	0.2871	53	2.12%	831.4	1.81%
FOXA1:AR(Forkhead,NR)/LNCAP-AR-ChIP-Seq(GSE27824)/Homer	AGTAAACAAAAAAGAACAND	1.00E+00	-1.94E+00	0.2886	18	0.72%	249.9	0.54%
Tcf3(HMG)/mES-Tcf3-ChIP-Seq(GSE11724)/Homer	ASWTCAAAGG	1.00E+00	-1.89E+00	0.3014	49	1.96%	768.1	1.67%
RUNX1(Runt)/Jurkat-RUNX1-ChIP-Seq(GSE29180)/Homer	AAACCACARM	1.00E+00	-1.85E+00	0.3113	214	8.56%	3666.7	7.99%
EBNA1(EBV virus)/Raji-EBNA1-ChIP-Seq(GSE30709)/Homer	GGYAGCAYDTGCTDCCCNNN	1.00E+00	-1.85E+00	0.3113	3	0.12%	25.8	0.06%
Ascl1(bHLH)/NeuralTubes-Ascl1-ChIP-Seq(GSE55840)/Homer	NNVVCAGCTGBN	1.00E+00	-1.78E+00	0.3307	309	12.36%	5377.7	11.73%
VDR(NR),DR3/GM10855-VDR+vitD-ChIP-Seq(GSE22484)/Homer	ARAGGTCANWGAGTTCANNN	1.00E+00	-1.77E+00	0.3314	38	1.52%	590.8	1.29%
Hoxb4(Homeobox)/ES-Hoxb4-ChIP-Seq(GSE34014)/Homer	TGATTRATGGCY	1.00E+00	-1.73E+00	0.3429	43	1.72%	678.4	1.48%
RUNX2(Runt)/PCa-RUNX2-ChIP-Seq(GSE33889)/Homer	NWAACCACADNN	1.00E+00	-1.72E+00	0.3436	179	7.16%	3065.6	6.68%
E2F1(E2F)/Hela-E2F1-ChIP-Seq(GSE22478)/Homer	CWGGCGGGAA	1.00E+00	-1.62E+00	0.377	25	1.00%	380.7	0.83%
E2F6(E2F)/Hela-E2F6-ChIP-Seq(GSE31477)/Homer	GGCGGGAARN	1.00E+00	-1.59E+00	0.3828	54	2.16%	878.6	1.92%
NFAT:AP1(RHD,bZIP)/Jurkat-NFATC1-ChIP-Seq(Jolma et al.)/Homer	SARTGGAAAAWRTGAGTCAB	1.00E+00	-1.59E+00	0.3828	31	1.24%	483.2	1.05%
E2F4(E2F)/K562-E2F4-ChIP-Seq(GSE31477)/Homer	GGCGGGAAAH	1.00E+00	-1.50E+00	0.4153	44	1.76%	713.2	1.56%
Sox2(HMG)/mES-Sox2-ChIP-Seq(GSE11431)/Homer	BCCATTGTTC	1.00E+00	-1.44E+00	0.4384	170	6.80%	2950.3	6.43%
Tbox:Smad(T-box,MAD)/ESCd5-Smad2_3-ChIP-Seq(GSE29422)/Homer	AGGTGHCAGACA	1.00E+00	-1.38E+00	0.4624	38	1.52%	619	1.35%
Hnf1(Homeobox)/Liver-Foxa2-Chip-Seq(GSE25694)/Homer	GGTTAAWCATTAA	1.00E+00	-1.38E+00	0.4624	27	1.08%	429.8	0.94%
CEBP(bZIP)/ThioMac-CEBPb-ChIP-Seq(GSE21512)/Homer	ATTGCGCAAC	1.00E+00	-1.37E+00	0.4624	161	6.44%	2801.1	6.11%
Atf4(bZIP)/MEF-Atf4-ChIP-Seq(GSE35681)/Homer	MTGATGCAAT	1.00E+00	-1.33E+00	0.4745	67	2.68%	1132.6	2.47%
ETS:RUNX(ETS,Runt)/Jurkat-RUNX1-ChIP-Seq(GSE17954)/Homer	RCAGGATGTGGT	1.00E+00	-1.30E+00	0.4857	14	0.56%	212.4	0.46%
Lhx3(Homeobox)/Neuron-Lhx3-ChIP-Seq(GSE31456)/Homer	ADBTAATTAR	1.00E+00	-1.29E+00	0.4886	368	14.72%	6552.9	14.29%
Pax7(Paired,Homeobox),longest/Myoblast-Pax7-ChIP-Seq(GSE25064)/Homer	NTAATTDGCYAATTANNWWD	1.00E+00	-1.21E+00	0.524	7	0.28%	99.3	0.22%
RBPJ:Ebox(?,bHLH)/Panc1-Rbpj1-ChIP-Seq(GSE47459)/Homer	GGGRAARRGRMCAGMTG	1.00E+00	-1.11E+00	0.5781	46	1.84%	785.5	1.71%
BORIS(Zf)/K562-CTCFL-ChIP-Seq(GSE32465)/Homer	CNNBRGCGCCCCCTGSTGGC	1.00E+00	-9.89E-01	0.6458	17	0.68%	282.4	0.62%
CEBP:AP1(bZIP)/ThioMac-CEBPb-ChIP-Seq(GSE21512)/Homer	DRTGTTGCAA	1.00E+00	-9.85E-01	0.6458	161	6.44%	2875.2	6.27%
ETS:E-box(ETS,bHLH)/HPC7-Scl-ChIP-Seq(GSE22178)/Homer	AGGAARCAGCTG	1.00E+00	-9.71E-01	0.649	15	0.60%	248.4	0.54%
HIF2a(bHLH)/785_O-HIF2a-ChIP-Seq(GSE34871)/Homer	GCACGTACCC	1.00E+00	-9.63E-01	0.6504	53	2.12%	927.3	2.02%
Myf5(bHLH)/GM-Myf5-ChIP-Seq(GSE24852)/Homer	BAACAGCTGT	1.00E+00	-9.55E-01	0.6512	119	4.76%	2120.6	4.62%

ZNF143|STAF(Zf)/CUTLL-ZNF143-ChIP-Seq(GSE29600)/Homer	ATTTCCCAGVAKSCY	1.00E+00	-9.40E-01	0.6572	51	2.04%	895	1.95%
ETV1(ETS)/GIST48-ETV1-ChIP-Seq(GSE22441)/Homer	AACCGGAAGT	1.00E+00	-9.32E-01	0.6579	212	8.48%	3814.1	8.32%
TCFL2(HMG)/K562-TCF7L2-ChIP-Seq(GSE29196)/Homer	ACWTCAAAGG	1.00E+00	-9.05E-01	0.6715	13	0.52%	217.4	0.47%
Maz(Zf)/HepG2-Maz-ChIP-Seq(GSE31477)/Homer	GGGGGGGG	1.00E+00	-8.50E-01	0.7051	152	6.08%	2742.9	5.98%
ELF5(ETS)/T47D-ELF5-ChIP-Seq(GSE30407)/Homer	ACVAGGAAGT	1.00E+00	-8.45E-01	0.7051	113	4.52%	2033.2	4.43%
Rfx5(HTH)/GM12878-Rfx5-ChIP-Seq(GSE31477)/Homer	SCCTAGCAACAG	1.00E+00	-8.43E-01	0.7051	52	2.08%	925.9	2.02%
CTCF(Zf)/CD4+-CTCF-ChIP-Seq(Barski et al.)/Homer	AYAGTGCCMYCTRGTGGCCA	1.00E+00	-8.34E-01	0.7051	11	0.44%	186.6	0.41%
ZNF711(Zf)/SHSY5Y-ZNF711-ChIP-Seq(GSE20673)/Homer	AGGCCTAG	1.00E+00	-8.19E-01	0.71	316	12.64%	5745.2	12.53%
Nanog(Homeobox)/mES-Nanog-ChIP-Seq(GSE11724)/Homer	RGCCATTAAC	1.00E+00	-8.18E-01	0.71	906	36.24%	16547.3	36.08%
Otx2(Homeobox)/EpiLC-Otx2-ChIP-Seq(GSE56098)/Homer	NYTAATCCYB	1.00E+00	-8.17E-01	0.71	180	7.20%	3261.4	7.11%
Elk4(ETS)/Hela-Elk4-ChIP-Seq(GSE31477)/Homer	NRYTTCCGGY	1.00E+00	-8.14E-01	0.71	63	2.52%	1129.1	2.46%
ZBTB33(Zf)/GM12878-ZBTB33-ChIP-Seq(GSE32465)/Homer	GGVTCTCGCGAGAAC	1.00E+00	-8.12E-01	0.71	3	0.12%	46	0.10%
TATA-Box(TBP)/Promoter/Homer	CCTTTTAWAGSC	1.00E+00	-8.09E-01	0.71	232	9.28%	4213.9	9.19%
STAT6(Stat)/CD4-Stat6-ChIP-Seq(GSE22104)/Homer	ABTTCYYRRGAA	1.00E+00	-8.02E-01	0.71	87	3.48%	1568.8	3.42%
HRE(HSF)/Striatum-HSF1-ChIP-Seq(GSE38000)/Homer	TTCTAGAABNTTCTA	1.00E+00	-7.99E-01	0.71	32	1.28%	568.5	1.24%
Sox6(HMG)/Myotubes-Sox6-ChIP-Seq(GSE32627)/Homer	CCATTGTTNY	1.00E+00	-7.74E-01	0.71	325	13.00%	5925.1	12.92%
HIF-1b(HLH)/T47D-HIF1b-ChIP-Seq(GSE59937)/Homer	RTACGTGC	1.00E+00	-7.62E-01	0.7126	170	6.80%	3093.5	6.75%
OCT4-SOX2-TCF-NANOG(POU,Homeobox,HMG)/mES-Oct4-ChIP-Seq(GSE11431)/Hon	ATTTGCATAACAATG	1.00E+00	-7.52E-01	0.7153	34	1.36%	610.4	1.33%
Sp1(Zf)/Promoter/Homer	GGCCCCGCCCCC	1.00E+00	-7.50E-01	0.7153	24	0.96%	428.2	0.93%
YY1(Zf)/Promoter/Homer	CAAGATGGCGGC	1.00E+00	-7.39E-01	0.7163	9	0.36%	156.9	0.34%
MyoG(bHLH)/C2C12-MyoG-ChIP-Seq(GSE36024)/Homer	AACAGCTG	1.00E+00	-6.45E-01	0.7824	177	7.08%	3255.4	7.10%
MyoD(bHLH)/Myotube-MyoD-ChIP-Seq(GSE21614)/Homer	RRCAGCTGYTSY	1.00E+00	-6.41E-01	0.7824	117	4.68%	2153.1	4.69%
CEBP:CEBP(bZIP)/MEF-Chop-ChIP-Seq(GSE35681)/Homer	NTNATGCAAYMNNHTGMAAY	1.00E+00	-6.41E-01	0.7824	28	1.12%	514.7	1.12%
Pax7(Paired,Homeobox)/Myoblast-Pax7-ChIP-Seq(GSE25064)/Homer	TAATCAATTA	1.00E+00	-6.34E-01	0.7824	20	0.80%	367.8	0.80%
Pit1(Homeobox)/GCrat-Pit1-ChIP-Seq(GSE58009)/Homer	ATGMATATDC	1.00E+00	-6.29E-01	0.7824	215	8.60%	3959.5	8.63%
Pax8(Paired,Homeobox)/Thyroid-Pax8-ChIP-Seq(GSE26938)/Homer	GTCATGCHTGRCTGS	1.00E+00	-6.25E-01	0.7824	58	2.32%	1070.3	2.33%
CArG(MADS)/PUER-Srf-ChIP-Seq(Sullivan et al.)/Homer	CCATATATGGNM	1.00E+00	-6.25E-01	0.7824	49	1.96%	904.9	1.97%
SpiB(ETS)/OCILY3-SPIB-ChIP-Seq(GSE56857)/Homer	AAAGRGGAAGTG	1.00E+00	-5.91E-01	0.7949	34	1.36%	632.5	1.38%
THRa(NR)/C17.2-THRa-ChIP-Seq(GSE38347)/Homer	GGTCANYTGAGGWCA	1.00E+00	-5.55E-01	0.8189	127	5.08%	2361.9	5.15%
Fli1(ETS)/CD8-FLI-ChIP-Seq(GSE20898)/Homer	NRYTTCCGGH	1.00E+00	-5.55E-01	0.8189	156	6.24%	2897.8	6.32%
ERG(ETS)/VCaP-ERG-ChIP-Seq(GSE14097)/Homer	ACAGGAAGTG	1.00E+00	-5.51E-01	0.8189	251	10.04%	4651.5	10.14%
GRE(NR),IR3/A549-GR-ChIP-Seq(GSE32465)/Homer	NRGVACABNVTGTYCY	1.00E+00	-4.86E-01	0.8635	18	0.72%	348	0.76%
ETS(ETS)/Promoter/Homer	AACCGGAAGT	1.00E+00	-4.62E-01	0.8803	36	1.44%	691.5	1.51%
Nkx6.1(Homeobox)/Islet-Nkx6.1-ChIP-Seq(GSE40975)/Homer	GKTAATGR	1.00E+00	-4.32E-01	0.9021	577	23.08%	10726.4	23.39%
PAX5(Paired,Homeobox)/GM12878-PAX5-ChIP-Seq(GSE32465)/Homer	GCAGCCAAGCRTGACH	1.00E+00	-4.12E-01	0.9153	60	2.40%	1154.1	2.52%
Znf263(Zf)/K562-Znf263-ChIP-Seq(GSE31477)/Homer	CVGTSCTCCC	1.00E+00	-3.99E-01	0.9228	275	11.00%	5166.2	11.26%
NFY(CCAAT)/Promoter/Homer	RGCCAATSRG	1.00E+00	-3.95E-01	0.9228	133	5.32%	2528	5.51%
Pax7(Paired,Homeobox),long/Myoblast-Pax7-ChIP-Seq(GSE25064)/Homer	TAATCHGATTAC	1.00E+00	-3.86E-01	0.9253	4	0.16%	85.4	0.19%
CHR(?)/Hela-CellCycle-Expression/Homer	SRGTTTCAAA	1.00E+00	-3.77E-01	0.9286	113	4.52%	2161	4.71%
EWS:ERG-fusion(ETS)/CADO_ES1-EWS:ERG-ChIP-Seq(SRA014231)/Homer	ATTTCCTGTN	1.00E+00	-3.68E-01	0.9325	119	4.76%	2276.8	4.96%

Isl1(Homeobox)/Neuron-Isl1-ChIP-Seq(GSE31456)/Homer	CTAATKGV	1.00E+00	-3.65E-01	0.9325	370	14.80%	6947.3	15.15%
NRF1(NRF)/MCF7-NRF1-ChIP-Seq(Unpublished)/Homer	CTGCGCATGCGC	1.00E+00	-3.61E-01	0.9325	6	0.24%	128.6	0.28%
T1ISRE(IRF)/ThioMac-Ifnb-Expression/Homer	ACTTTCGTTTCT	1.00E+00	-3.59E-01	0.9325	1	0.04%	22	0.05%
Arnt:Ahr(bHLH)/MCF7-Arnt-ChIP-Seq(Lo et al.)/Homer	TBGCACGCAA	1.00E+00	-3.54E-01	0.9325	82	3.28%	1586.3	3.46%
Chop(bZIP)/MEF-Chop-ChIP-Seq(GSE35681)/Homer	ATTGCATCAT	1.00E+00	-3.44E-01	0.9325	48	1.92%	945.2	2.06%
PU.1(ETS)/ThioMac-PU.1-ChIP-Seq(GSE21512)/Homer	AGAGGAAGTG	1.00E+00	-3.36E-01	0.9336	72	2.88%	1403.5	3.06%
GFX(?)/Promoter/Homer	ATTCTCGCGAGA	1.00E+00	-3.36E-01	0.9336	1	0.04%	23.4	0.05%
EHF(ETS)/LoVo-EHF-ChIP-Seq(GSE49402)/Homer	AVCAGGAAGT	1.00E+00	-3.36E-01	0.9336	197	7.88%	3749.1	8.17%
Pbx3(Homeobox)/GM12878-PBX3-ChIP-Seq(GSE32465)/Homer	SCTGTCAMTCAN	1.00E+00	-3.28E-01	0.9336	37	1.48%	739.1	1.61%
Tbx20(T-box)/Heart-Tbx20-ChIP-Seq(GSE29636)/Homer	GGTGYTGACAGS	1.00E+00	-3.28E-01	0.9336	37	1.48%	739.3	1.61%
GLI3(Zf)/Limb-GLI3-ChIP-Chip(GSE11077)/Homer	CGTGGGTGGTCC	1.00E+00	-2.93E-01	0.9465	14	0.56%	298	0.65%
IRF2(IRF)/Erythroblas-IRF2-ChIP-Seq(GSE36985)/Homer	GAAASYGAAASY	1.00E+00	-2.93E-01	0.9465	14	0.56%	298.5	0.65%
p53(p53)/Saos-p53-ChIP-Seq(GSE15780)/Homer	RRCATGYCYRGRCATGYYYN	1.00E+00	-2.93E-01	0.9465	12	0.48%	258.2	0.56%
p53(p53)/Saos-p53-ChIP-Seq/Homer	RRCATGYCYRGRCATGYYYN	1.00E+00	-2.93E-01	0.9465	12	0.48%	258.2	0.56%
PRDM9(Zf)/Testis-DMC1-ChIP-Seq(GSE35498)/Homer	ADGGYAGYAGCATCT	1.00E+00	-2.93E-01	0.9465	64	2.56%	1266.3	2.76%
IRF1(IRF)/PBMC-IRF1-ChIP-Seq(GSE43036)/Homer	GAAAGTGAAAGT	1.00E+00	-2.70E-01	0.9503	19	0.76%	402.3	0.88%
EWS:FLI1-fusion(ETS)/SK_N_MC-EWS:FLI1-ChIP-Seq(SRA014231)/Homer	VACAGGAAAT	1.00E+00	-2.55E-01	0.9607	77	3.08%	1529.1	3.33%
IRF4(IRF)/GM12878-IRF4-ChIP-Seq(GSE32465)/Homer	ACTGAAACCA	1.00E+00	-2.41E-01	0.9699	75	3.00%	1497.8	3.27%
ISRE(IRF)/ThioMac-LPS-Expression(GSE23622)/Homer	AGTTTCASTTTC	1.00E+00	-2.32E-01	0.9732	8	0.32%	186.4	0.41%
PRDM1(Zf)/Hela-PRDM1-ChIP-Seq(GSE31477)/Homer	ACTTTCACTTTC	1.00E+00	-2.21E-01	0.9797	88	3.52%	1755.4	3.83%
AR-halfsite(NR)/LNCaP-AR-ChIP-Seq(GSE27824)/Homer	CCAGGAACAG	1.00E+00	-2.14E-01	0.982	725	29.00%	13655.2	29.77%
PBX1(Homeobox)/MCF7-PBX1-ChIP-Seq(GSE28007)/Homer	GSCTGTCACTCA	1.00E+00	-2.10E-01	0.982	11	0.44%	253.8	0.55%
n-Myc(bHLH)/mES-nMyc-ChIP-Seq(GSE11431)/Homer	VRCCACGTGG	1.00E+00	-1.79E-01	1	89	3.56%	1798.1	3.92%
Elk1(ETS)/Hela-Elk1-ChIP-Seq(GSE31477)/Homer	HACTTCCGGY	1.00E+00	-1.63E-01	1	55	2.20%	1148.8	2.50%
Oct2(POU,Homeobox)/Bcell-Oct2-ChIP-Seq(GSE21512)/Homer	ATATGCAAAT	1.00E+00	-1.62E-01	1	56	2.24%	1169	2.55%
Oct4(POU,Homeobox)/mES-Oct4-ChIP-Seq(GSE11431)/Homer	ATTTGCATAW	1.00E+00	-1.59E-01	1	88	3.52%	1792	3.91%
c-Myc(bHLH)/mES-cMyc-ChIP-Seq(GSE11431)/Homer	VVCCACGTGG	1.00E+00	-1.51E-01	1	61	2.44%	1272.2	2.77%
p53(p53)/mES-cMyc-ChIP-Seq(GSE11431)/Homer	ACATGCCCGGGCAT	1.00E+00	-1.47E-01	1	2	0.08%	64.3	0.14%
GABPA(ETS)/Jurkat-GABPa-ChIP-Seq(GSE17954)/Homer	RACCGGAAGT	1.00E+00	-1.43E-01	1	118	4.72%	2381.9	5.19%
HOXA2(Homeobox)/mES-Hoxa2-ChIP-Seq(Donaldson et al.)/Homer	GYCATCMATCAT	1.00E+00	-1.19E-01	1	14	0.56%	342.5	0.75%
NRF(NRF)/Promoter/Homer	STGCGCATGCGC	1.00E+00	-1.02E-01	1	8	0.32%	217.5	0.47%
ELF1(ETS)/Jurkat-ELF1-ChIP-Seq(SRA014231)/Homer	AVCCGGAAGT	1.00E+00	-8.72E-02	1	49	1.96%	1079.1	2.35%
Lhx2(Homeobox)/HFSC-Lhx2-ChIP-Seq(GSE48068)/Homer	TAATTAGN	1.00E+00	-5.63E-02	1	194	7.76%	3958.3	8.63%
NFkB-p50,p52(RHD)/Monocyte-p50-ChIP-Chip(Schreiber et al.)/Homer	GGGGGAATCCCC	1.00E+00	-5.46E-02	1	7	0.28%	215.3	0.47%
E-box(bHLH)/Promoter/Homer	SSGGTCACGTGA	1.00E+00	-5.07E-02	1	6	0.24%	193.3	0.42%
ETS1(ETS)/Jurkat-ETS1-ChIP-Seq(GSE17954)/Homer	ACAGGAAGTG	1.00E+00	-4.51E-02	1	135	5.40%	2839.9	6.19%
Max(bHLH)/K562-Max-ChIP-Seq(GSE31477)/Homer	RCCACGTGGYYN	1.00E+00	-3.85E-02	1	86	3.44%	1886	4.11%
bHLHE40(bHLH)/HepG2-BHLHE40-ChIP-Seq(GSE31477)/Homer	KCACGTGMCN	1.00E+00	-2.74E-02	1	30	1.20%	758.1	1.65%
Pit1+1bp(Homeobox)/GCrat-Pit1-ChIP-Seq(GSE58009)/Homer	ATGCATAATTCA	1.00E+00	-2.65E-02	1	53	2.12%	1244.2	2.71%
CRX(Homeobox)/Retina-Crx-ChIP-Seq(GSE20012)/Homer	GCTAATCC	1.00E+00	-2.44E-02	1	577	23.08%	11353.9	24.76%

Gfi1b(Zf)/HPC7-Gfi1b-ChIP-Seq(GSE22178)/Homer	MAATCACTGC	1.00E+00	-2.44E-02	1	141	5.64%	3019.7	6.58%
Mouse_Recombination_Hotspot(Zf)/Testis-DMC1-ChIP-Seq(GSE24438)/Homer	ACTYKNATTCGTGNTACTTC	1.00E+00	-1.87E-02	1	5	0.20%	196.6	0.43%
HNF6(Homeobox)/Liver-Hnf6-ChIP-Seq(ERP000394)/Homer	NTATYGATCH	1.00E+00	-1.71E-02	1	94	3.76%	2113.9	4.61%
PU.1-IRF(ETS:IRF)/Bcell-PU.1-ChIP-Seq(GSE21512)/Homer	MGGAAGTGAAAC	1.00E+00	-7.67E-03	1	188	7.52%	4059.9	8.85%
Ptf1a(bHLH)/Panc1-Ptf1a-ChIP-Seq(GSE47459)/Homer	ACAGCTGTTN	1.00E+00	-7.34E-03	1	528	21.12%	10613.3	23.14%
Tbet(T-box)/CD8-Tbet-ChIP-Seq(GSE33802)/Homer	AGGTGTGAAM	1.00E+00	-6.62E-03	1	210	8.40%	4507.8	9.83%
USF1(bHLH)/GM12878-Usf1-ChIP-Seq(GSE32465)/Homer	SGTCACGTGR	1.00E+00	-6.09E-03	1	49	1.96%	1248.4	2.72%
Usf2(bHLH)/C2C12-Usf2-ChIP-Seq(GSE36030)/Homer	GTCACGTGGT	1.00E+00	-5.21E-03	1	45	1.80%	1170.3	2.55%
CLOCK(bHLH)/Liver-Clock-ChIP-Seq(GSE39860)/Homer	GHCACGTG	1.00E+00	-4.37E-03	1	63	2.52%	1566.9	3.42%
Srebp1a(bHLH)/HepG2-Srebp1a-ChIP-Seq(GSE31477)/Homer	RTCACSCCAY	1.00E+00	-2.58E-03	1	34	1.36%	961.1	2.10%
E2A(bHLH)/proBcell-E2A-ChIP-Seq(GSE21978)/Homer	DNRCAGCTGY	1.00E+00	-2.57E-03	1	277	11.08%	5917.9	12.90%
c-Myc(bHLH)/LNCAP-cMyc-ChIP-Seq(Unpublished)/Homer	VCCACGTG	1.00E+00	-1.09E-03	1	33	1.32%	976.3	2.13%
Srebp2(bHLH)/HepG2-Srebp2-ChIP-Seq(GSE31477)/Homer	CGGTCACSCCAC	1.00E+00	-5.63E-04	1	21	0.84%	716.3	1.56%
NPAS2(bHLH)/Liver-NPAS2-ChIP-Seq(GSE39860)/Homer	KCCACGTGAC	1.00E+00	-5.07E-04	1	175	7.00%	4029.7	8.79%
LXRE(NR),DR4/RAW-LXRb.biotin-ChIP-Seq(GSE21512)/Homer	RGGTTACTANAGGTCA	1.00E+00	-2.59E-04	1	27	1.08%	891.5	1.94%
Nkx3.1(Homeobox)/LNCaP-Nkx3.1-ChIP-Seq(GSE28264)/Homer	AAGCACTTAA	1.00E+00	-2.14E-04	1	444	17.76%	9420.3	20.54%
Pitx1(Homeobox)/Chicken-Pitx1-ChIP-Seq(GSE38910)/Homer	TAATCCCN	1.00E+00	-1.14E-04	1	950	38.00%	19078.1	41.60%
E2A(bHLH),near_PU.1/Bcell-PU.1-ChIP-Seq(GSE21512)/Homer	NVCACCTGBN	1.00E+00	-5.60E-05	1	283	11.32%	6377	13.90%
BMAL1(bHLH)/Liver-Bmal1-ChIP-Seq(GSE39860)/Homer	GNCACGTG	1.00E+00	-1.20E-05	1	279	11.16%	6412.2	13.98%
GSC(Homeobox)/FrogEmbryos-GSC-ChIP-Seq(DRA000576)/Homer	RGGATTAR	1.00E+00	-9.00E-06	1	321	12.84%	7281.1	15.88%
Nkx2.1(Homeobox)/LungAC-Nkx2.1-ChIP-Seq(GSE43252)/Homer	RSCACTYRAG	1.00E+00	-1.00E-06	1	507	20.28%	11162.8	24.34%
Nkx2.5(Homeobox)/HL1-Nkx2.5.biotin-ChIP-Seq(GSE21529)/Homer	RRSCACTYAA	1.00E+00	-1.00E-06	1	381	15.24%	8691.2	18.95%
Eomes(T-box)/H9-Eomes-ChIP-Seq(GSE26097)/Homer	ATTAACACCT	1.00E+00	0.00E+00	1	373	14.92%	8586.6	18.72%
Tbx5(T-box)/HL1-Tbx5.biotin-ChIP-Seq(GSE21529)/Homer	AGGTGTCA	1.00E+00	0.00E+00	1	653	26.12%	14333.2	31.25%
Mef2a(MADS)/HL1-Mef2a.biotin-ChIP-Seq(GSE21529)/Homer	CYAAAAATAG	1.00E+00	0.00E+00	1	64	2.56%	2261.1	4.93%
Mef2c(MADS)/GM12878-Mef2c-ChIP-Seq(GSE32465)/Homer	DCYAAAAATAGM	1.00E+00	0.00E+00	1	66	2.64%	2469.2	5.38%
REST-NRSF(Zf)/Jurkat-NRSF-ChIP-Seq/Homer	GGMGCTGTCCATGGTGCTGA	1.00E+00	0.00E+00	1	0	0.00%	5.3	0.01%
CTCF-SatelliteElement(Zf?)/CD4+-CTCF-ChIP-Seq(Barski et al.)/Homer	TGCAGTTCCMVNWRTGGCCA	1.00E+00	0.00E+00	1	0	0.00%	27.8	0.06%
