# Supplementary material for: Genomic modelling of the ESR1 Y537S mutation for evaluating function and new therapeutic approaches for metastatic breast cancer
Source: Oncogene. 2016 Oct 17;36(16):2286–96. doi: 10.1038/onc.2016.382 (PMC5245767; doi:10.1038/onc.2016.382)
Supplement: Supplementary Table 3 [file onc2016382x5.pdf]

MCF7 E2 v MCF7 Vehicle GSEA Hallmarks

Pathways UP

NAME	SIZE	ES	NES	NOM p-val	FDR q-val	FWER p-val	RANK AT MAX
HALLMARK_E2F_TARGETS	199	0.637	10.741	0.0000	0.0000	0.0000	6857
HALLMARK_MYC_TARGETS_V1	199	0.571	9.472	0.0000	0.0000	0.0000	8334
HALLMARK_ESTROGEN_RESPONSE_EARLY	200	0.502	8.282	0.0000	0.0000	0.0000	3012
HALLMARK_MYC_TARGETS_V2	58	0.811	7.497	0.0000	0.0000	0.0000	4095
HALLMARK_G2M_CHECKPOINT	198	0.439	7.415	0.0000	0.0000	0.0000	7727
HALLMARK_ESTROGEN_RESPONSE_LATE	198	0.438	7.187	0.0000	0.0000	0.0000	6261
HALLMARK_MTORC1_SIGNALING	199	0.274	4.493	0.0000	0.0000	0.0000	8741
HALLMARK_DNA_REPAIR	142	0.318	4.384	0.0000	0.0000	0.0000	6876
HALLMARK_OXIDATIVE_PHOSPHORYLATION	198	0.222	3.565	0.0000	0.0000	0.0000	8430
HALLMARK_UNFOLDED_PROTEIN_RESPONSE	112	0.287	3.528	0.0000	0.0000	0.0000	5819
HALLMARK_FATTY_ACID_METABOLISM	148	0.193	2.800	0.0000	0.0000	0.0000	8500
HALLMARK_ADIPOGENESIS	191	0.159	2.543	0.0000	0.0001	0.0020	7254
HALLMARK_GLYCOLYSIS	196	0.144	2.340	0.0020	0.0008	0.0150	5427
HALLMARK_SPERMATOGENESIS	120	0.150	1.913	0.0019	0.0102	0.1970	7413
HALLMARK_UV_RESPONSE_UP	156	0.126	1.805	0.0179	0.0166	0.3210	6726
HALLMARK_BILE_ACID_METABOLISM	106	0.071	0.866	0.5903	0.6182	1.0000	6843

Pathways DOWN

NAME	SIZE	ES	NES	NOM p-val	FDR q-val	FWER p-val	RANK AT MAX
HALLMARK_TNFA_SIGNALING_VIA_NFKB	190	-0.424	-6.840	0.0000	0.0000	0.0000	4035
HALLMARK_HYPOXIA	194	-0.299	-4.860	0.0000	0.0000	0.0000	5242
HALLMARK_EPITHELIAL_MESENCHYMAL_TRANSITION	186	-0.295	-4.687	0.0000	0.0000	0.0000	6607
HALLMARK_APICAL_JUNCTION	194	-0.293	-4.609	0.0000	0.0000	0.0000	6801
HALLMARK_MITOTIC_SPINDLE	200	-0.278	-4.517	0.0000	0.0000	0.0000	6031
HALLMARK_APOPTOSIS	154	-0.298	-4.271	0.0000	0.0000	0.0000	6402
HALLMARK_TGF_BETA_SIGNALING	54	-0.477	-4.108	0.0000	0.0000	0.0000	7272
HALLMARK_P53_PATHWAY	196	-0.248	-3.945	0.0000	0.0000	0.0000	6611
HALLMARK_COAGULATION	112	-0.307	-3.896	0.0000	0.0000	0.0000	7869
HALLMARK_HEME_METABOLISM	186	-0.249	-3.873	0.0000	0.0000	0.0000	7641
HALLMARK_PI3K_AKT_MTOR_SIGNALING	103	-0.330	-3.872	0.0000	0.0000	0.0000	6935
HALLMARK_PROTEIN_SECRETION	94	-0.337	-3.863	0.0000	0.0000	0.0000	8942
HALLMARK_UV_RESPONSE_DN	141	-0.270	-3.762	0.0000	0.0000	0.0000	6529
HALLMARK_MYOGENESIS	188	-0.233	-3.728	0.0000	0.0000	0.0000	5431
HALLMARK_ANDROGEN_RESPONSE	98	-0.310	-3.593	0.0000	0.0000	0.0000	7174
HALLMARK_INFLAMMATORY_RESPONSE	182	-0.220	-3.490	0.0000	0.0000	0.0000	8335
HALLMARK_KRAS_SIGNALING_UP	181	-0.213	-3.348	0.0000	0.0000	0.0000	2901
HALLMARK_COMPLEMENT	181	-0.212	-3.331	0.0000	0.0000	0.0000	6364
HALLMARK_INTERFERON_GAMMA_RESPONSE	191	-0.201	-3.238	0.0000	0.0000	0.0000	10352
HALLMARK_IL2_STAT5_SIGNALING	187	-0.202	-3.149	0.0000	0.0000	0.0000	7683
HALLMARK_IL6_JAK_STAT3_SIGNALING	76	-0.286	-2.836	0.0000	0.0000	0.0000	6732
HALLMARK_NOTCH_SIGNALING	32	-0.417	-2.814	0.0000	0.0000	0.0000	5248
HALLMARK_CHOLESTEROL_HOMEOSTASIS	72	-0.273	-2.758	0.0000	0.0001	0.0010	7557
HALLMARK_INTERFERON_ALPHA_RESPONSE	93	-0.212	-2.382	0.0000	0.0006	0.0110	4569
HALLMARK_ANGIOGENESIS	31	-0.354	-2.355	0.0000	0.0006	0.0110	2913
HALLMARK_REACTIVE_OXIGEN_SPECIES_PATHWAY	47	-0.286	-2.348	0.0000	0.0006	0.0120	10434
HALLMARK_XENOBIOTIC_METABOLISM	180	-0.147	-2.263	0.0000	0.0010	0.0190	2595
HALLMARK_ALLOGRAFT_REJECTION	173	-0.145	-2.215	0.0000	0.0014	0.0270	5841
HALLMARK_APICAL_SURFACE	43	-0.281	-2.143	0.0000	0.0021	0.0430	7538
HALLMARK_PEROXISOME	98	-0.158	-1.873	0.0162	0.0123	0.2390	7419
HALLMARK_WNT_BETA_CATENIN_SIGNALING	42	-0.201	-1.541	0.0646	0.0638	0.7590	6729
HALLMARK_KRAS_SIGNALING_DN	168	-0.099	-1.525	0.0467	0.0666	0.7910	5556
HALLMARK_PANCREAS_BETA_CELLS	31	-0.198	-1.301	0.1552	0.1618	0.9780	9409
HALLMARK_HEDGEHOG_SIGNALING	36	-0.145	-1.054	0.3770	0.3726	1.0000	4746

Y537S E2 v MCF7 Vehicle GSEA Hallmarks

Pathways UP

NAME	SIZE	ES	NES	NOM p-val	FDR q-val	FWER p-val	RANK AT MAX
HALLMARK_E2F_TARGETS	199	0.6036	9.9394	0.0000	0.0000	0.0000	6418
HALLMARK_G2M_CHECKPOINT	198	0.4399	7.0980	0.0000	0.0000	0.0000	6418
HALLMARK_ESTROGEN_RESPONSE_LATE	198	0.3949	6.5398	0.0000	0.0000	0.0000	4090
HALLMARK_MYC_TARGETS_V1	199	0.3803	6.2734	0.0000	0.0000	0.0000	11892
HALLMARK_ESTROGEN_RESPONSE_EARLY	200	0.3667	6.0830	0.0000	0.0000	0.0000	3167
HALLMARK_FATTY_ACID_METABOLISM	146	0.2687	3.8157	0.0000	0.0000	0.0000	8016
HALLMARK_MTORC1_SIGNALING	197	0.2247	3.6519	0.0000	0.0000	0.0000	7835
HALLMARK_OXIDATIVE_PHOSPHORYLATION	197	0.2195	3.5668	0.0000	0.0000	0.0000	15523
HALLMARK_ADIPOGENESIS	189	0.1994	3.2300	0.0000	0.0000	0.0000	15803
HALLMARK_MYC_TARGETS_V2	58	0.3477	3.0990	0.0000	0.0000	0.0000	14218
HALLMARK_GLYCOLYSIS	195	0.1790	2.9317	0.0000	0.0000	0.0000	8104
HALLMARK_MITOTIC_SPINDLE	200	0.1732	2.9022	0.0000	0.0000	0.0000	2512
HALLMARK_PEROXISOME	100	0.2366	2.7508	0.0000	0.0000	0.0000	8688
HALLMARK_DNA_REPAIR	142	0.1826	2.5710	0.0000	0.0001	0.0020	8287
HALLMARK_CHOLESTEROL_HOMEOSTASIS	70	0.2095	2.0417	0.0043	0.0043	0.0810	7495
HALLMARK_XENOBIOTIC_METABOLISM	180	0.1257	1.9646	0.0101	0.0076	0.1380	8016
HALLMARK_SPERMATOGENESIS	118	0.1501	1.9128	0.0020	0.0099	0.1880	3475
HALLMARK_BILE_ACID_METABOLISM	103	0.1380	1.6711	0.0405	0.0338	0.5300	5777
HALLMARK_PROTEIN_SECRETION	93	0.0893	1.0265	0.3989	0.4255	1.0000	23562
HALLMARK_UNFOLDED_PROTEIN_RESPONSE	112	0.0821	1.0159	0.4339	0.4169	1.0000	8120

Pathways DOWN

NAME	SIZE	ES	NES	NOM p-val	FDR q-val	FWER p-val	RANK AT MAX
HALLMARK_EPITHELIAL_MESENCHYMAL_TRANSITION	185	-0.3612	-5.6764	0.0000	0.0000	0.0000	5345
HALLMARK_TNFA_SIGNALING_VIA_NFKB	188	-0.3322	-5.1330	0.0000	0.0000	0.0000	4552
HALLMARK_APICAL_JUNCTION	189	-0.2974	-4.7494	0.0000	0.0000	0.0000	7733
HALLMARK_INTERFERON_GAMMA_RESPONSE	185	-0.2612	-4.1382	0.0000	0.0000	0.0000	8647
HALLMARK_MYOGENESIS	185	-0.2538	-3.9765	0.0000	0.0000	0.0000	9936
HALLMARK_INTERFERON_ALPHA_RESPONSE	92	-0.3304	-3.7743	0.0000	0.0000	0.0000	4256
HALLMARK_INFLAMMATORY_RESPONSE	177	-0.2469	-3.7574	0.0000	0.0000	0.0000	8647
HALLMARK_KRAS_SIGNALING_UP	172	-0.2322	-3.5358	0.0000	0.0000	0.0000	7192
HALLMARK_IL6_JAK_STAT3_SIGNALING	73	-0.3107	-3.1912	0.0000	0.0000	0.0000	4845
HALLMARK_TGF_BETA_SIGNALING	54	-0.3592	-3.1102	0.0000	0.0000	0.0000	8273
HALLMARK_HYPOXIA	192	-0.1825	-3.0488	0.0000	0.0000	0.0000	4940
HALLMARK_UV_RESPONSE_DN	141	-0.1975	-2.6947	0.0000	0.0000	0.0000	6340
HALLMARK_ALLOGRAFT_REJECTION	165	-0.1710	-2.5222	0.0000	0.0005	0.0050	8647
HALLMARK_COAGULATION	112	-0.1868	-2.3180	0.0000	0.0012	0.0140	3358
HALLMARK_APOPTOSIS	152	-0.1580	-2.3150	0.0020	0.0012	0.0140	4531
HALLMARK_KRAS_SIGNALING_DN	162	-0.1541	-2.2680	0.0000	0.0018	0.0220	8319
HALLMARK_WNT_BETA_CATENIN_SIGNALING	41	-0.3004	-2.2654	0.0022	0.0017	0.0230	7457
HALLMARK_ANGIOGENESIS	31	-0.3272	-2.1222	0.0000	0.0043	0.0620	3565
HALLMARK_UV_RESPONSE_UP	154	-0.1463	-2.1017	0.0043	0.0048	0.0710	7900
HALLMARK_P53_PATHWAY	193	-0.1329	-2.0943	0.0019	0.0048	0.0750	3779
HALLMARK_HEDGEHOG_SIGNALING	34	-0.2852	-1.9899	0.0117	0.0084	0.1370	4467
HALLMARK_ANDROGEN_RESPONSE	98	-0.1699	-1.9819	0.0061	0.0083	0.1420	5658
HALLMARK_NOTCH_SIGNALING	31	-0.2962	-1.9779	0.0041	0.0081	0.1430	7213
HALLMARK_PI3K_AKT_MTOR_SIGNALING	100	-0.1615	-1.9181	0.0099	0.0114	0.2020	8828
HALLMARK_IL2_STAT5_SIGNALING	186	-0.1183	-1.8906	0.0137	0.0129	0.2330	7546
HALLMARK_COMPLEMENT	174	-0.1045	-1.6028	0.0415	0.0504	0.6690	9183
HALLMARK_PANCREAS_BETA_CELLS	31	-0.2245	-1.4598	0.0860	0.0937	0.8830	1887
HALLMARK_HEME_METABOLISM	182	-0.0823	-1.2705	0.2012	0.1942	0.9900	9906
HALLMARK_APICAL_SURFACE	42	-0.1524	-1.1725	0.2647	0.2651	0.9990	9936
HALLMARK_REACTIVE_OXIGEN_SPECIES_PATHWAY	47	-0.1180	-0.9880	0.4488	0.4486	1.0000	9400

Y537S E2 v Y537S Vehicle GSEA Hallmarks

Pathways UP

NAME	SIZE	ES	NES	NOM p-val	FDR q-val	FWER p-val	RANK AT MAX
HALLMARK_ESTROGEN_RESPONSE_EARLY	200	0.5835	9.6045	0.0000	0.0000	0.0000	4330
HALLMARK_MYC_TARGETS_V1	199	0.5419	8.7847	0.0000	0.0000	0.0000	7569
HALLMARK_E2F_TARGETS	199	0.5039	8.3105	0.0000	0.0000	0.0000	5068
HALLMARK_ESTROGEN_RESPONSE_LATE	196	0.5057	8.1250	0.0000	0.0000	0.0000	4066
HALLMARK_MYC_TARGETS_V2	58	0.7776	6.8721	0.0000	0.0000	0.0000	4176
HALLMARK_MTORC1_SIGNALING	198	0.3768	6.1500	0.0000	0.0000	0.0000	6382
HALLMARK_G2M_CHECKPOINT	198	0.3816	6.0949	0.0000	0.0000	0.0000	7205
HALLMARK_UNFOLDED_PROTEIN_RESPONSE	110	0.3160	3.8786	0.0000	0.0000	0.0000	7679
HALLMARK_GLYCOLYSIS	194	0.2393	3.8279	0.0000	0.0000	0.0000	6647
HALLMARK_OXIDATIVE_PHOSPHORYLATION	196	0.1827	2.9093	0.0000	0.0001	0.0010	8440
HALLMARK_DNA_REPAIR	141	0.1968	2.7671	0.0000	0.0001	0.0010	5994
HALLMARK_FATTY_ACID_METABOLISM	146	0.1980	2.7392	0.0020	0.0001	0.0020	8158
HALLMARK_HYPOXIA	188	0.1664	2.6522	0.0000	0.0002	0.0040	5287
HALLMARK_UV_RESPONSE_UP	153	0.1349	1.9641	0.0063	0.0094	0.1490	5186
HALLMARK_PEROXISOME	97	0.1711	1.9386	0.0062	0.0102	0.1710	5400
HALLMARK_ALLOGRAFT_REJECTION	147	0.1144	1.6199	0.0466	0.0486	0.6250	4628
HALLMARK_INTERFERON_ALPHA_RESPONSE	93	0.1433	1.6194	0.0342	0.0458	0.6250	9182
HALLMARK_SPERMATOGENESIS	109	0.0911	1.1238	0.3057	0.3127	1.0000	6201
HALLMARK_PANCREAS_BETA_CELLS	24	0.1094	0.6471	0.8973	0.9040	1.0000	22856

Pathways DOWN

NAME	SIZE	ES	NES	NOM p-val	FDR q-val	FWER p-val	RANK AT MAX
HALLMARK_TNFA_SIGNALING_VIA_NFKB	182	-0.2522	-4.0138	0.0000	0.0000	0.0000	3465
HALLMARK_MITOTIC_SPINDLE	200	-0.2502	-3.9437	0.0000	0.0000	0.0000	7179
HALLMARK_TGF_BETA_SIGNALING	53	-0.4362	-3.7845	0.0000	0.0000	0.0000	5550
HALLMARK_P53_PATHWAY	194	-0.2358	-3.7204	0.0000	0.0000	0.0000	6742
HALLMARK_APOPTOSIS	146	-0.2410	-3.3567	0.0000	0.0000	0.0000	7932
HALLMARK_MYOGENESIS	174	-0.2123	-3.2759	0.0000	0.0000	0.0000	4843
HALLMARK_APICAL_JUNCTION	179	-0.2145	-3.2590	0.0000	0.0000	0.0000	4641
HALLMARK_HEME_METABOLISM	179	-0.2151	-3.2568	0.0000	0.0000	0.0000	6581
HALLMARK_UV_RESPONSE_DN	137	-0.2387	-3.2531	0.0000	0.0000	0.0000	4613
HALLMARK_COMPLEMENT	163	-0.1962	-2.9633	0.0000	0.0000	0.0000	7965
HALLMARK_PROTEIN_SECRETION	93	-0.2537	-2.8613	0.0000	0.0000	0.0000	7089
HALLMARK_XENOBIOTIC_METABOLISM	173	-0.1692	-2.5891	0.0000	0.0002	0.0020	5902
HALLMARK_ANDROGEN_RESPONSE	96	-0.2047	-2.2954	0.0000	0.0025	0.0260	6850
HALLMARK_COAGULATION	106	-0.1766	-2.1302	0.0040	0.0054	0.0610	7965
HALLMARK_ADIPOGENESIS	186	-0.1308	-2.1197	0.0020	0.0055	0.0650	5622
HALLMARK_CHOLESTEROL_HOMEOSTASIS	70	-0.2149	-2.0951	0.0058	0.0055	0.0690	7692
HALLMARK_ANGIOGENESIS	30	-0.3205	-2.0923	0.0000	0.0053	0.0700	4843
HALLMARK_PI3K_AKT_MTOR_SIGNALING	98	-0.1766	-2.0797	0.0000	0.0055	0.0770	10373
HALLMARK_NOTCH_SIGNALING	31	-0.3142	-2.0594	0.0042	0.0058	0.0850	5448
HALLMARK_EPITHELIAL_MESENCHYMAL_TRANSITION	177	-0.1306	-1.9916	0.0101	0.0089	0.1350	5786
HALLMARK_IL2_STAT5_SIGNALING	180	-0.1260	-1.9660	0.0077	0.0104	0.1600	4858
HALLMARK_WNT_BETA_CATENIN_SIGNALING	41	-0.2559	-1.9350	0.0059	0.0117	0.1860	4941
HALLMARK_INTERFERON_GAMMA_RESPONSE	177	-0.1247	-1.8952	0.0145	0.0134	0.2200	8681
HALLMARK_KRAS_SIGNALING_DN	154	-0.1257	-1.8361	0.0059	0.0179	0.2970	1007
HALLMARK_IL6_JAK_STAT3_SIGNALING	70	-0.1579	-1.5952	0.0439	0.0553	0.6690	4991
HALLMARK_KRAS_SIGNALING_UP	160	-0.1050	-1.5549	0.0691	0.0648	0.7350	4257
HALLMARK_REACTIVE_OXIGEN_SPECIES_PATHWAY	45	-0.1924	-1.5076	0.0679	0.0762	0.8060	8589
HALLMARK_APICAL_SURFACE	41	-0.1922	-1.4885	0.0558	0.0796	0.8310	2713
HALLMARK_INFLAMMATORY_RESPONSE	157	-0.0922	-1.3508	0.1168	0.1377	0.9600	7480
HALLMARK_HEDGEHOG_SIGNALING	33	-0.1964	-1.3393	0.1273	0.1400	0.9680	3695
HALLMARK_BILE_ACID_METABOLISM	96	-0.1136	-1.3154	0.1406	0.1486	0.9780	6488

Y537S Vehicle v MCF7 Vehicle GSEA Hallmarks

Pathways UP

NAME	SIZE	ES	NES	NOM p-val	FDR q-val	FWER p-val	RANK AT MAX
HALLMARK_E2F_TARGETS	199	0.5741	9.2427	0.0000	0.0000	0.0000	4976
HALLMARK_G2M_CHECKPOINT	198	0.4290	6.9073	0.0000	0.0000	0.0000	2410
HALLMARK_ESTROGEN_RESPONSE_LATE	198	0.3099	5.0675	0.0000	0.0000	0.0000	5219
HALLMARK_ESTROGEN_RESPONSE_EARLY	200	0.2937	4.7879	0.0000	0.0000	0.0000	3965
HALLMARK_MYC_TARGETS_V1	199	0.2466	3.9966	0.0000	0.0000	0.0000	12713
HALLMARK_OXIDATIVE_PHOSPHORYLATION	197	0.2325	3.8251	0.0000	0.0000	0.0000	15062
HALLMARK_FATTY_ACID_METABOLISM	145	0.2731	3.7409	0.0000	0.0000	0.0000	7236
HALLMARK_DNA_REPAIR	142	0.2191	3.0387	0.0000	0.0000	0.0000	9294
HALLMARK_MITOTIC_SPINDLE	199	0.1846	3.0309	0.0000	0.0000	0.0000	1803
HALLMARK_GLYCOLYSIS	195	0.1847	3.0124	0.0000	0.0000	0.0000	9440
HALLMARK_MTORC1_SIGNALING	198	0.1845	2.9904	0.0000	0.0000	0.0000	9224
HALLMARK_ADIPOGENESIS	187	0.1738	2.7648	0.0000	0.0000	0.0000	12526
HALLMARK_XENOBIOTIC_METABOLISM	178	0.1659	2.5567	0.0000	0.0001	0.0020	7512
HALLMARK_PEROXISOME	99	0.2024	2.3594	0.0000	0.0005	0.0080	6669
HALLMARK_SPERMATOGENESIS	116	0.1643	2.0856	0.0020	0.0037	0.0670	5193
HALLMARK_CHOLESTEROL_HOMEOSTASIS	71	0.2066	2.0558	0.0040	0.0043	0.0800	7012
HALLMARK_P53_PATHWAY	195	0.1265	2.0476	0.0000	0.0042	0.0840	7570
HALLMARK_MYC_TARGETS_V2	58	0.1278	1.1246	0.2857	0.3296	1.0000	23527
HALLMARK_BILE_ACID_METABOLISM	104	0.0940	1.1200	0.3157	0.3168	1.0000	6960
HALLMARK_PROTEIN_SECRETION	93	0.0786	0.8988	0.5596	0.5763	1.0000	21796

Pathways DOWN

NAME	SIZE	ES	NES	NOM p-val	FDR q-val	FWER p-val	RANK AT MAX
HALLMARK_EPITHELIAL_MESENCHYMAL_TRANSITION	186	-0.3941	-6.2928	0.0000	0.0000	0.0000	7580
HALLMARK_TNFA_SIGNALING_VIA_NFKB	189	-0.3416	-5.4234	0.0000	0.0000	0.0000	4530
HALLMARK_INTERFERON_GAMMA_RESPONSE	187	-0.2728	-4.3703	0.0000	0.0000	0.0000	10321
HALLMARK_INTERFERON_ALPHA_RESPONSE	93	-0.3637	-4.1530	0.0000	0.0000	0.0000	5022
HALLMARK_APICAL_JUNCTION	190	-0.2669	-4.1365	0.0000	0.0000	0.0000	6583
HALLMARK_KRAS_SIGNALING_UP	174	-0.2626	-4.0157	0.0000	0.0000	0.0000	7677
HALLMARK_INFLAMMATORY_RESPONSE	176	-0.2522	-3.8305	0.0000	0.0000	0.0000	3404
HALLMARK_MYOGENESIS	187	-0.2247	-3.6526	0.0000	0.0000	0.0000	6172
HALLMARK_HYPOXIA	193	-0.2113	-3.4601	0.0000	0.0000	0.0000	4530
HALLMARK_IL6_JAK_STAT3_SIGNALING	74	-0.3081	-3.1555	0.0000	0.0000	0.0000	4362
HALLMARK_ALLOGRAFT_REJECTION	161	-0.2063	-2.9326	0.0000	0.0000	0.0000	4981
HALLMARK_UV_RESPONSE_DN	141	-0.2135	-2.9271	0.0000	0.0000	0.0000	7127
HALLMARK_TGF_BETA_SIGNALING	54	-0.3344	-2.8322	0.0000	0.0000	0.0000	6890
HALLMARK_ANDROGEN_RESPONSE	98	-0.2033	-2.3803	0.0000	0.0008	0.0100	3847
HALLMARK_COAGULATION	112	-0.1794	-2.2020	0.0000	0.0031	0.0400	3070
HALLMARK_APOPTOSIS	152	-0.1493	-2.1331	0.0020	0.0040	0.0530	8003
HALLMARK_IL2_STAT5_SIGNALING	187	-0.1336	-2.1021	0.0000	0.0045	0.0630	8152
HALLMARK_ANGIOGENESIS	31	-0.3165	-2.0742	0.0039	0.0051	0.0760	5732
HALLMARK_UV_RESPONSE_UP	155	-0.1340	-1.9929	0.0080	0.0076	0.1160	8386
HALLMARK_HEDGEHOG_SIGNALING	34	-0.2921	-1.9920	0.0020	0.0074	0.1180	3441
HALLMARK_COMPLEMENT	174	-0.1258	-1.9175	0.0039	0.0113	0.1830	8279
HALLMARK_NOTCH_SIGNALING	31	-0.2803	-1.8939	0.0078	0.0125	0.2120	6770
HALLMARK_KRAS_SIGNALING_DN	163	-0.1215	-1.7846	0.0120	0.0206	0.3300	6563
HALLMARK_PANCREAS_BETA_CELLS	30	-0.2653	-1.7249	0.0202	0.0280	0.4360	5780
HALLMARK_WNT_BETA_CATENIN_SIGNALING	41	-0.2274	-1.7187	0.0232	0.0278	0.4470	5378
HALLMARK_PI3K_AKT_MTOR_SIGNALING	101	-0.1303	-1.5074	0.0604	0.0781	0.8270	10187
HALLMARK_APICAL_SURFACE	41	-0.1638	-1.2625	0.1945	0.2014	0.9930	5100
HALLMARK_REACTIVE_OXIGEN_SPECIES_PATHWAY	47	-0.1553	-1.2467	0.1931	0.2064	0.9950	8364
HALLMARK_HEME_METABOLISM	183	-0.0777	-1.2319	0.2012	0.2113	0.9970	8126
HALLMARK_UNFOLDED_PROTEIN_RESPONSE	112	-0.0875	-1.0825	0.3395	0.3416	1.0000	10301

Y537S Vehicle v MCF7 E2 GSEA Hallmarks

Pathways UP

NAME	SIZE	ES	NES	NOM p-val	FDR q-val	FWER p-val	RANK AT MAX
HALLMARK_E2F_TARGETS	199	0.4886	8.0210	0.0000	0.0000	0.0000	5448
HALLMARK_G2M_CHECKPOINT	198	0.3841	6.3741	0.0000	0.0000	0.0000	1969
HALLMARK_FATTY_ACID_METABOLISM	148	0.3157	4.5562	0.0000	0.0000	0.0000	8125
HALLMARK_MITOTIC_SPINDLE	200	0.2203	3.6817	0.0000	0.0000	0.0000	6842
HALLMARK_ADIPOGENESIS	191	0.1969	3.1920	0.0000	0.0000	0.0000	7377
HALLMARK_ESTROGEN_RESPONSE_LATE	198	0.1969	3.1499	0.0000	0.0000	0.0000	6772
HALLMARK_GLYCOLYSIS	194	0.1924	3.0508	0.0000	0.0000	0.0000	4915
HALLMARK_XENOBIOTIC_METABOLISM	177	0.1924	2.9593	0.0000	0.0000	0.0000	8043
HALLMARK_PEROXISOME	98	0.2413	2.8387	0.0000	0.0000	0.0000	4560
HALLMARK_OXIDATIVE_PHOSPHORYLATION	198	0.1708	2.7872	0.0000	0.0000	0.0000	8106
HALLMARK_CHOLESTEROL_HOMEOSTASIS	73	0.2663	2.6805	0.0000	0.0001	0.0010	5798
HALLMARK_APOPTOSIS	151	0.1806	2.6322	0.0000	0.0001	0.0010	5923
HALLMARK_PROTEIN_SECRETION	94	0.2257	2.5565	0.0000	0.0003	0.0050	8893
HALLMARK_MTORC1_SIGNALING	199	0.1566	2.5554	0.0000	0.0003	0.0050	5829
HALLMARK_P53_PATHWAY	195	0.1523	2.4935	0.0000	0.0003	0.0060	6610
HALLMARK_DNA_REPAIR	142	0.1572	2.2282	0.0020	0.0016	0.0280	7707
HALLMARK_COMPLEMENT	176	0.1326	2.0944	0.0000	0.0036	0.0680	8032
HALLMARK_HEME_METABOLISM	185	0.1115	1.8105	0.0159	0.0186	0.3140	5401
HALLMARK_MYC_TARGETS_V1	199	0.1091	1.7737	0.0175	0.0218	0.3740	9380
HALLMARK_SPERMATOGENESIS	112	0.1381	1.7146	0.0340	0.0284	0.4760	1964
HALLMARK_BILE_ACID_METABOLISM	103	0.1194	1.4351	0.0922	0.0986	0.9170	6397
HALLMARK_REACTIVE_OXIGEN_SPECIES_PATHWAY	46	0.1307	1.0439	0.3516	0.3802	1.0000	6296

Pathways DOWN

NAME	SIZE	ES	NES	NOM p-val	FDR q-val	FWER p-val	RANK AT MAX
HALLMARK_EPITHELIAL_MESENCHYMAL_TRANSITION	187	-0.3667	-5.8666	0.0000	0.0000	0.0000	4784
HALLMARK_INTERFERON_ALPHA_RESPONSE	93	-0.4084	-4.7171	0.0000	0.0000	0.0000	5653
HALLMARK_INTERFERON_GAMMA_RESPONSE	188	-0.2757	-4.4402	0.0000	0.0000	0.0000	5627
HALLMARK_ESTROGEN_RESPONSE_EARLY	200	-0.2558	-4.3163	0.0000	0.0000	0.0000	4520
HALLMARK_MYC_TARGETS_V2	58	-0.4436	-4.0213	0.0000	0.0000	0.0000	7156
HALLMARK_KRAS_SIGNALING_UP	175	-0.2283	-3.4952	0.0000	0.0000	0.0000	8491
HALLMARK_TNFA_SIGNALING_VIA_NFKB	190	-0.2150	-3.4302	0.0000	0.0000	0.0000	6469
HALLMARK_INFLAMMATORY_RESPONSE	175	-0.2164	-3.3099	0.0000	0.0000	0.0000	6847
HALLMARK_APICAL_JUNCTION	192	-0.1984	-3.3033	0.0000	0.0000	0.0000	7569
HALLMARK_MYOGENESIS	185	-0.2122	-3.2842	0.0000	0.0000	0.0000	5010
HALLMARK_ALLOGRAFT_REJECTION	169	-0.1823	-2.8068	0.0000	0.0000	0.0000	14807
HALLMARK_UV_RESPONSE_DN	141	-0.2048	-2.7987	0.0000	0.0000	0.0000	4985
HALLMARK_HYPOXIA	192	-0.1695	-2.7640	0.0000	0.0000	0.0000	6439
HALLMARK_UNFOLDED_PROTEIN_RESPONSE	111	-0.2104	-2.5889	0.0000	0.0001	0.0010	7762
HALLMARK_IL6_JAK_STAT3_SIGNALING	76	-0.2453	-2.4683	0.0000	0.0004	0.0060	10448
HALLMARK_UV_RESPONSE_UP	155	-0.1427	-2.1228	0.0020	0.0041	0.0560	5421
HALLMARK_COAGULATION	111	-0.1688	-2.0450	0.0020	0.0067	0.0940	2960
HALLMARK_IL2_STAT5_SIGNALING	185	-0.1268	-2.0301	0.0040	0.0071	0.1040	6059
HALLMARK_ANDROGEN_RESPONSE	98	-0.1704	-1.9749	0.0060	0.0088	0.1350	6611
HALLMARK_HEDGEHOG_SIGNALING	36	-0.2641	-1.8480	0.0122	0.0172	0.2650	6844
HALLMARK_WNT_BETA_CATENIN_SIGNALING	42	-0.2336	-1.7723	0.0267	0.0245	0.3640	8418
HALLMARK_ANGIOGENESIS	31	-0.2622	-1.7375	0.0256	0.0286	0.4330	4561
HALLMARK_PANCREAS_BETA_CELLS	29	-0.2667	-1.6882	0.0219	0.0351	0.5210	4270
HALLMARK_NOTCH_SIGNALING	32	-0.2345	-1.5792	0.0541	0.0563	0.7090	4984
HALLMARK_APICAL_SURFACE	43	-0.1958	-1.5270	0.0479	0.0691	0.7940	6465
HALLMARK_TGF_BETA_SIGNALING	54	-0.1735	-1.4756	0.0725	0.0835	0.8680	10007
HALLMARK_KRAS_SIGNALING_DN	164	-0.0918	-1.3710	0.1241	0.1260	0.9500	3364
HALLMARK_PI3K_AKT_MTOR_SIGNALING	101	-0.0934	-1.1092	0.3088	0.3169	1.0000	7345
