# Supplementary material for: Genomic modelling of the ESR1 Y537S mutation for evaluating function and new therapeutic approaches for metastatic breast cancer
Source: Oncogene. 2016 Oct 17;36(16):2286–96. doi: 10.1038/onc.2016.382 (PMC5245767; doi:10.1038/onc.2016.382)
Supplement: Supplementary Table 4 [file onc2016382x6.pdf]

CLUSTER 1	Normalised Expression												Mean Expression Difference			
	MCF7			MCF7			Y537S			Y537S			MCF7	MCF7	Y537S	Y537S
Gene	Vehicle			Estrogen			Vehicle			Estrogen			Veh	E2	Veh	E2
	1	2	3	1	2	3	1	2	3	1	2					
TBC1D14	2226	2245	2212	2623	2510	2501	2677	2712	2770	2752	2836		1.0	1.1	1.2	1.3
GPD1L	1138	1141	1053	1511	1537	1460	1723	1725	1792	1774	2019		1.0	1.4	1.6	1.7
ORC3	620	634	634	789	770	750	896	912	865	894	987		1.0	1.2	1.4	1.5
PCNT	2571	2620	2593	3050	3007	2986	3465	3318	3610	3302	3561		1.0	1.2	1.3	1.3
TMEM123	5356	5342	5329	5855	6087	6050	6488	6558	6406	6659	6857		1.0	1.1	1.2	1.3
MCM4	3742	3823	3795	5022	5079	5043	6880	6649	6858	7421	7709		1.0	1.3	1.8	2.0
UNG	1528	1710	1533	2644	2605	2614	4234	4265	4189	5312	5333		1.0	1.6	2.7	3.3
FAM198B	780	843	791	1215	1292	1313	2133	2120	1956	2458	2396		1.0	1.6	2.6	3.0
IFITM10	97	108	103	347	318	370	1056	1125	1040	1414	1447		1.0	3.4	10.4	13.9
C6orf211	2235	2295	2259	3364	3488	3302	5282	5609	5275	5750	6286		1.0	1.5	2.4	2.7
CTSC	201	211	194	316	378	317	611	619	566	729	731		1.0	1.7	3.0	3.6
A4GALT	132	148	132	407	315	328	612	574	607	858	835		1.0	2.6	4.4	6.2
ASB13	657	654	633	1156	1058	1057	1425	1431	1545	1683	1711		1.0	1.7	2.3	2.6
ELOVL6	1067	1104	1025	1790	1801	1748	2265	2458	2244	2754	2755		1.0	1.7	2.2	2.6
ANAPC13	1395	1540	1405	1851	1753	1768	2058	2180	2159	2295	2269		1.0	1.2	1.5	1.6
LMNB1	2762	3003	2841	4005	3828	3764	4979	4836	5151	5312	5449		1.0	1.3	1.7	1.9
MCM2	1958	2017	1934	3716	3533	3446	5665	5423	5546	6561	6416		1.0	1.8	2.8	3.3
CDT1	931	938	929	1415	1325	1306	1912	1894	1938	2077	2061		1.0	1.4	2.1	2.2
ESR1	2652	2716	2684	3312	3232	3192	3652	3730	3823	3749	3851		1.0	1.2	1.4	1.4
MEPCE	4450	4513	4339	4951	5083	5087	5537	5433	5727	5893	5789		1.0	1.1	1.3	1.3
C16orf74	272	275	237	373	393	390	490	472	490	553	515		1.0	1.5	1.9	2.0
GALNT7	1115	1205	1201	1297	1468	1378	1494	1524	1470	1548	1671		1.0	1.2	1.3	1.4
CYB5D1	416	474	468	536	583	609	641	632	690	734	690		1.0	1.3	1.4	1.6
UBE4B	1637	1763	1768	2182	2268	2197	2392	2651	2681	2914	2937		1.0	1.3	1.5	1.7
FAM162A	754	825	722	891	951	912	1164	1241	1037	1178	1275		1.0	1.2	1.5	1.6
ABHD17C	1989	2009	1866	2254	2373	2318	2706	2763	2544	3026	3099		1.0	1.2	1.4	1.6
CHPT1	363	366	325	421	509	436	616	668	550	701	692		1.0	1.3	1.7	2.0
MRPL16	931	937	895	1073	1136	1128	1353	1229	1159	1269	1301		1.0	1.2	1.4	1.4
RBM8A	8518	8945	8389	9002	9358	9350	9487	9958	9847	10012	9829		1.0	1.1	1.1	1.2
RPL14	7966	8096	7826	8325	8668	9230	9296	9338	9275	9197	9381		1.0	1.1	1.2	1.2
SLC19A2	720	654	625	927	928	906	963	1037	903	1011	1159		1.0	1.4	1.5	1.6
UMPS	1989	1811	1913	2340	2432	2246	2340	2601	2363	2582	2724		1.0	1.2	1.3	1.4
EBPL	506	441	474	610	642	616	731	797	660	729	780		1.0	1.3	1.5	1.6
RPRD2	2541	2403	2594	3072	3038	2962	3531	3553	3640	3515	3750		1.0	1.2	1.4	1.4
ZNF488	32	23	37	95	91	83	154	147	171	170	260		1.0	2.9	5.1	7.0
TMEM229B	153	98	172	294	261	258	445	428	467	538	533		1.0	1.9	3.2	3.8
OPA1	2938	2833	2886	3152	3236	3121	3430	3356	3499	3496	3680		1.0	1.1	1.2	1.2
DDX20	849	732	788	957	1065	914	1222	1041	1147	1223	1271		1.0	1.2	1.4	1.6
WBP11	3455	3166	3298	4060	3946	4094	4677	4484	4699	5102	5183		1.0	1.2	1.4	1.6
CEP85	320	279	276	417	380	396	494	521	515	668	678		1.0	1.4	1.7	2.3
MLKL	148	98	107	194	217	187	227	249	292	379	351		1.0	1.7	2.2	3.1
XRCC3	1615	1390	1534	1713	1799	1733	1979	2043	2066	2293	2280		1.0	1.2	1.3	1.5
GPI	8507	8413	8480	9426	9072	9383	9308	9831	9434	10059	9998		1.0	1.1	1.1	1.2
TMEM109	2926	2814	2934	3730	3585	3773	3862	4181	4093	4465	4689		1.0	1.3	1.4	1.6
GINS3	153	173	159	283	260	288	318	379	286	429	442		1.0	1.7	2.0	2.7
FOS	403	466	429	614	552	646	618	687	671	837	843		1.0	1.4	1.5	1.9
KCNK6	915	939	957	1120	1165	1228	1182	1316	1159	1569	1483		1.0	1.2	1.3	1.6
TMA16	694	730	692	874	934	910	973	1006	885	1228	1161		1.0	1.3	1.4	1.7
CDC123	1909	1989	1941	2243	2252	2336	2495	2456	2441	2773	2899		1.0	1.2	1.3	1.5
FAM60A	1967	1960	1907	2111	2143	2316	2442	2384	2352	2472	2699		1.0	1.1	1.2	1.3
EMG1	1048	1155	1060	1270	1264	1327	1314	1376	1289	1357	1557		1.0	1.2	1.2	1.3
R3HDM1	2180	1969	2124	2616	2630	2503	2556	2599	2615	2801	3067		1.0	1.2	1.2	1.4
MTIF2	1158	1072	1113	1386	1379	1332	1464	1346	1352	1517	1630		1.0	1.2	1.2	1.4
NUP155	1926	1821	1936	2340	2472	2429	2504	2382	2498	2555	2869		1.0	1.3	1.3	1.4
PGAM1	12733	12397	12418	13270	13420	13160	13331	13335	13068	13711	14213		1.0	1.1	1.1	1.1
PDIA6	13335	13224	12731	13778	13780	14156	14626	14514	14161	15574	15619		1.0	1.1	1.1	1.2
ERLIN2	1402	1446	1259	1549	1667	1731	1898	1834	1720	2247	2195		1.0	1.2	1.3	1.6
DHX57	1423	1453	1341	1599	1714	1700	2015	1956	1835	2936	3182		1.0	1.2	1.4	2.2
SLC25A24	5014	5058	4795	5480	5585	5870	6508	6482	6190	7972	7960		1.0	1.1	1.3	1.6
CD47	5262	5097	4935	5622	5433	5681	5920	5954	5829	6503	6877		1.0	1.1	1.2	1.3
KCNF1	667	675	647	805	769	868	904	1069	925	1396	1295		1.0	1.2	1.5	2.0
SLC1A5	10624	10463	10391	11334	11449	11403	12260	12339	12306	13608	13343		1.0	1.1	1.2	1.3

DAP3	3318	3264	3190	3584	3692	3533	3883	3966	3842	4335	4362	1.0	1.1	1.2	1.3
ADCY1	1026	1000	1047	1388	1394	1359	1639	1704	1627	2350	2382	1.0	1.3	1.6	2.3
DOK7	582	589	591	1175	1082	1155	1658	1736	1640	2978	2800	1.0	1.9	2.9	4.9
RFWD3	2795	2719	2748	3257	3189	3140	3598	3563	3522	4201	4037	1.0	1.2	1.3	1.5
TPBG	5169	5531	5655	6604	6173	6500	7734	8001	7374	9968	10114	1.0	1.2	1.4	1.8
NT5DC3	318	310	325	450	445	436	550	619	537	790	829	1.0	1.4	1.8	2.5
PLOD2	7736	7754	7699	10156	10601	10081	13543	14439	12953	17856	18762	1.0	1.3	1.8	2.4
KCTD3	3568	3624	3552	4114	3921	3953	4365	4472	4258	4774	4865	1.0	1.1	1.2	1.3
C16orf13	1006	1070	1049	1227	1165	1283	1286	1221	1244	1373	1406	1.0	1.2	1.2	1.3
MRPL17	1150	1233	1238	1434	1409	1476	1518	1488	1452	1703	1730	1.0	1.2	1.2	1.4
GEMIN6	284	346	301	417	408	416	498	405	441	562	527	1.0	1.3	1.4	1.8
GLRX5	2721	2963	2723	3104	3130	3165	3253	3261	3354	3552	3420	1.0	1.1	1.2	1.2
TMEM147	1916	2169	1938	2361	2313	2312	2417	2354	2412	2684	2561	1.0	1.2	1.2	1.3
ALDH18A1	3705	3901	3789	4190	4173	4234	4276	4258	4286	4636	4497	1.0	1.1	1.1	1.2
HNRNPA1	31108	32466	31246	35070	34429	35388	36450	35918	37180	37412	36687	1.0	1.1	1.2	1.2
THNSL1	456	455	436	687	699	715	937	760	849	980	892	1.0	1.6	1.9	2.1
THYN1	155	189	149	246	232	237	362	309	303	379	348	1.0	1.5	2.0	2.2
ST7	341	418	353	470	475	509	567	547	540	601	613	1.0	1.3	1.5	1.6
PRMT7	579	646	658	865	729	833	946	924	834	965	950	1.0	1.3	1.4	1.5
LRRC45	694	755	749	944	822	925	987	961	948	1112	1010	1.0	1.2	1.3	1.4
CACYBP	4438	4400	4160	4821	4849	4919	4912	5103	4800	5270	5325	1.0	1.1	1.1	1.2
C21orf33	3580	3634	3574	3956	3923	3913	3930	4037	3796	4049	4074	1.0	1.1	1.1	1.1
SEPHS2	4289	4543	4147	4849	4663	4683	4847	4900	4719	5091	5122	1.0	1.1	1.1	1.2
TSPAN31	3218	3168	3106	4024	3633	3808	4072	3886	3840	4419	4395	1.0	1.2	1.2	1.4
WDR75	1170	1150	1074	1479	1400	1358	1500	1481	1489	1592	1726	1.0	1.2	1.3	1.5
THEM4	247	257	264	328	328	369	381	331	350	361	387	1.0	1.3	1.4	1.5
PPA1	4135	4251	4055	4479	4509	4647	4722	4523	4696	4652	4857	1.0	1.1	1.1	1.1
PPIL1	1130	1035	1052	1295	1350	1369	1352	1297	1279	1498	1478	1.0	1.2	1.2	1.4
RPL7L1	3824	3705	3633	4066	4172	4271	4273	4187	4068	4391	4358	1.0	1.1	1.1	1.2
MLH3	679	628	645	777	830	863	898	866	831	933	898	1.0	1.3	1.3	1.4
LZIC	514	454	460	593	569	682	743	646	611	743	753	1.0	1.3	1.4	1.6
CLNS1A	2613	2533	2371	2705	2781	2936	3123	2847	2836	3207	3209	1.0	1.1	1.2	1.3
CHD1L	1900	1821	1814	2063	2084	2189	2245	1989	2057	2291	2265	1.0	1.1	1.1	1.2
ANGEL1	1459	1309	1366	1645	1620	1646	1735	1645	1644	1753	1614	1.0	1.2	1.2	1.2
SNRPA	1572	1528	1428	1775	1685	1805	1982	1842	1917	2153	1869	1.0	1.2	1.3	1.3
ATP5G1	1171	1082	1173	1333	1285	1338	1405	1350	1484	1498	1410	1.0	1.2	1.2	1.3
PBX1	1901	1954	1906	2595	2449	2543	2877	2582	2831	2741	2712	1.0	1.3	1.4	1.4
MTPAP	916	915	911	1102	1094	1133	1291	1207	1231	1218	1249	1.0	1.2	1.4	1.3
SCIN	2997	2908	2953	4009	3854	3845	4821	4575	4605	4525	4330	1.0	1.3	1.6	1.5
COX15	838	807	857	1003	1061	960	1190	1128	1128	1165	1078	1.0	1.2	1.4	1.3
CABLES1	1679	1569	1572	2051	1826	1953	2120	2253	2135	2104	2072	1.0	1.2	1.4	1.3
FAM189B	977	926	956	1246	1121	1153	1277	1345	1228	1334	1348	1.0	1.2	1.3	1.4
TMED10	10610	10304	10279	11258	11017	10892	11452	11566	11402	11517	11538	1.0	1.1	1.1	1.1
LDLRAD3	749	728	801	980	948	1032	986	976	978	1105	1249	1.0	1.3	1.3	1.5
HSPA14	1315	1378	1347	1540	1604	1597	1597	1576	1555	1694	1799	1.0	1.2	1.2	1.3
CCDC138	127	141	149	206	222	214	228	234	241	264	315	1.0	1.5	1.7	2.1
INO80B	486	513	538	677	655	667	704	715	683	702	803	1.0	1.3	1.4	1.5
PFKM	1052	1094	1185	1606	1532	1613	1582	1692	1499	1775	1990	1.0	1.4	1.4	1.7
TIPIN	156	175	201	263	263	269	265	270	248	346	323	1.0	1.5	1.5	1.9
REPIN1	3988	4197	4225	4759	4585	4674	4597	4807	4739	5085	5020	1.0	1.1	1.1	1.2
TTF2	1177	1157	1105	1610	1524	1476	1865	1670	1663	1997	1910	1.0	1.3	1.5	1.7
E2F2	75	71	63	160	146	124	191	206	173	278	253	1.0	2.1	2.7	3.8
LYPD6	518	447	426	632	660	571	749	693	666	820	809	1.0	1.3	1.5	1.8
DYNC2H1	107	110	90	150	175	150	185	176	187	214	227	1.0	1.5	1.8	2.2
TXNRD3	352	330	315	447	443	418	483	456	509	525	524	1.0	1.3	1.5	1.6
POLR3K	1098	1065	974	1296	1359	1309	1412	1315	1426	1506	1467	1.0	1.3	1.3	1.4
EPS15L1	1311	1316	1327	2449	2432	2386	2468	2390	2588	3249	3259	1.0	1.8	1.9	2.5
SLC29A1	601	590	664	1263	1182	1201	1295	1221	1297	1802	1644	1.0	2.0	2.1	2.8
**GREB1**	**637**	**743**	**753**	**10398**	**10077**	**9437**	**16212**	**16529**	**16182**	**44266**	**43856**	**1.0**	**14.0**	**22.9**	**62.0**
NPY1R	51	45	40	481	380	469	601	599	702	1652	1632	1.0	9.8	14.0	36.3
EMP2	10447	10754	10809	19195	18436	18919	21267	20782	22195	28197	28071	1.0	1.8	2.0	2.6
NFIA	269	311	294	504	497	512	518	527	577	679	633	1.0	1.7	1.9	2.3
PTCD3	1546	1559	1529	1948	1992	1992	2099	1965	2050	2252	2171	1.0	1.3	1.3	1.4
CDCA7	162	170	226	494	542	498	540	538	516	714	784	1.0	2.8	2.9	4.0
C19orf48	1935	1903	2010	2758	2639	2863	2938	2773	2779	3039	3105	1.0	1.4	1.5	1.6
ALKBH2	537	553	554	745	727	780	780	756	730	840	896	1.0	1.4	1.4	1.6

PODXL2	1934	2000	2023	2652	2552	2736	2894	2848	2803	3041	2828	1.0	1.3	1.4	1.5
IRS1	8290	8537	8533	11416	11251	11029	11285	11904	11382	12546	11890	1.0	1.3	1.4	1.4
DHRS2	936	1023	908	1607	1598	1614	1829	1764	1807	1969	2069	1.0	1.7	1.9	2.1
**TFF1**	**3877**	**3865**	**3866**	**26053**	**24771**	**24606**	**42563**	**51429**	**41678**	**63939**	**64727**	**1.0**	**6.5**	**11.7**	**16.6**
PAPSS2	4607	4677	4507	6162	6139	6243	6869	7033	6793	7251	7220	1.0	1.3	1.5	1.6
DSCAM-AS1	4101	3924	3852	7204	6386	6682	7033	7153	6875	8737	8732	1.0	1.7	1.8	2.2
TET2	1741	1648	1723	5066	4736	4669	4767	4584	4991	6972	7021	1.0	2.8	2.8	4.1
C5AR2	25	13	21	310	304	290	289	294	360	638	677	1.0	15.3	15.9	33.3
KCNK5	142	153	126	466	417	489	421	449	443	602	603	1.0	3.3	3.1	4.3
MYB	80	107	73	456	465	485	535	489	465	846	896	1.0	5.4	5.7	10.0
CMSS1	627	640	591	985	993	1009	1116	1002	1028	1291	1412	1.0	1.6	1.7	2.2
PAICS	4808	4602	4472	6802	6935	6801	6883	6690	6970	8523	8542	1.0	1.5	1.5	1.8
RAB31	2037	1930	2037	3843	3957	3961	4098	3990	3774	5850	5977	1.0	2.0	2.0	3.0
CDK4	5180	5069	5065	7095	7121	6998	7199	7133	7070	8361	8638	1.0	1.4	1.4	1.7
RAPGEFL1	1145	1152	1117	2443	2232	2420	2240	2225	2234	3711	3551	1.0	2.1	2.0	3.2
SIAH2	1749	1792	1709	2424	2413	2418	2397	2316	2330	2870	2933	1.0	1.4	1.3	1.7
SLC39A6	8408	8559	8662	12745	12074	12539	11958	12150	11890	14550	15308	1.0	1.5	1.4	1.7
POLD2	1617	1595	1637	2052	2153	2061	2065	2065	2023	2332	2154	1.0	1.3	1.3	1.4
NOB1	2393	2289	2303	2710	2743	2674	2631	2718	2659	2851	2814	1.0	1.2	1.1	1.2
NTHL1	253	260	309	389	406	363	405	457	456	463	473	1.0	1.4	1.6	1.7
GTPBP3	494	477	523	664	639	608	616	661	718	732	717	1.0	1.3	1.3	1.5
ZNRF1	3611	3608	3777	4364	4269	4356	4766	4538	4592	5067	5107	1.0	1.2	1.3	1.4
GLB1L2	412	400	429	715	676	694	925	833	752	1050	1068	1.0	1.7	2.0	2.6
**NRIP1**	**1169**	**1167**	**1326**	**1998**	**2072**	**1951**	**2296**	**2139**	**2225**	**2923**	**2975**	**1.0**	**1.6**	**1.8**	**2.4**
UBQLN4	3770	3702	3806	4659	4492	4521	4640	4774	4646	5297	5302	1.0	1.2	1.2	1.4
STEAP3	1819	1664	1853	2309	2255	2247	2438	2414	2312	2655	2599	1.0	1.3	1.3	1.5
GPR68	6	4	8	72	47	59	105	81	132	247	230	1.0	9.6	17.1	38.3
CISH	238	245	303	510	401	507	567	523	577	845	758	1.0	1.8	2.1	3.1
NPM3	808	868	879	1052	1037	1098	1169	1132	1192	1313	1311	1.0	1.2	1.4	1.5
DIS3L	409	420	419	622	629	545	703	605	727	743	821	1.0	1.4	1.6	1.9
**CA12**	**1143**	**1148**	**1221**	**2746**	**2613**	**2610**	**4183**	**3913**	**3876**	**5778**	**5996**	**1.0**	**2.3**	**3.4**	**5.0**
**EGR3**	**292**	**322**	**313**	**1119**	**1103**	**1137**	**2048**	**2094**	**2145**	**4232**	**4299**	**1.0**	**3.6**	**6.8**	**13.8**
CAND1	8498	8573	8438	9832	10160	9932	10984	10864	10718	11670	12115	1.0	1.2	1.3	1.4
RASGRP1	2086	2037	2100	4013	3976	3905	5446	4895	5149	6524	6661	1.0	1.9	2.5	3.2
MTHFD1	3156	3227	3027	4381	4329	4274	5131	4715	5000	5735	5915	1.0	1.4	1.6	1.9
GINS2	887	855	783	1447	1402	1321	1683	1662	1689	1981	2102	1.0	1.7	2.0	2.4
DCLRE1B	953	952	997	1431	1465	1313	1630	1583	1671	1764	1828	1.0	1.5	1.7	1.9
CDC6	476	500	514	880	886	765	1084	1066	1179	1371	1421	1.0	1.7	2.2	2.8
DTL	114	114	137	272	290	280	384	387	440	547	511	1.0	2.3	3.3	4.3
AMZ1	157	206	169	421	464	375	501	513	570	825	771	1.0	2.4	3.0	4.5
ADAT1	1411	1444	1465	1723	1706	1637	1774	1766	1743	1856	1860	1.0	1.2	1.2	1.3
MYO19	1435	1448	1497	1902	1809	1630	1913	1785	1798	2044	1926	1.0	1.2	1.3	1.4
OXA1L	2562	2656	2485	2807	2848	2939	3679	3292	3402	3312	3331	1.0	1.1	1.3	1.3
RCCD1	459	437	425	574	512	566	774	705	693	685	575	1.0	1.3	1.6	1.4
ACKR3	2150	2279	2229	2580	2389	2506	4230	4059	4327	3529	3916	1.0	1.1	1.9	1.7
NR2F2	6680	6945	7012	7716	7297	7684	10819	11230	10773	9784	10021	1.0	1.1	1.6	1.4
LSS	7808	7522	7648	8398	8116	8073	9321	9435	9387	9233	8943	1.0	1.1	1.2	1.2
B3GALNT1	2884	2876	2564	3325	3267	3279	4572	4522	4728	5226	5600	1.0	1.2	1.7	2.0
RBBP8	834	804	756	1052	1032	1010	1477	1367	1415	1813	1879	1.0	1.3	1.8	2.3
NAA50	9861	9520	9214	10779	11185	10999	13062	13032	13048	14733	15336	1.0	1.2	1.4	1.6
HELLS	475	454	388	625	639	582	947	952	909	1080	1139	1.0	1.4	2.1	2.5
MXI1	335	289	261	407	430	431	645	555	572	724	744	1.0	1.4	2.0	2.5
ALDH3A2	1582	1584	1508	1842	1710	1816	2141	2119	1962	2278	2351	1.0	1.1	1.3	1.5
SF3B3	12123	11835	12138	13780	13592	13375	15725	15464	15840	16344	16476	1.0	1.1	1.3	1.4
BRCA1	298	281	314	489	441	433	850	713	767	878	933	1.0	1.5	2.6	3.0
WISP2	1344	1303	1260	2174	1941	1864	3325	3206	3137	4039	3860	1.0	1.5	2.5	3.0
FEN1	1301	1320	1305	1938	1808	1777	2634	2684	2707	3214	3205	1.0	1.4	2.0	2.5
PPM1K	298	272	281	564	442	517	1057	1116	1094	1385	1484	1.0	1.8	3.8	5.1
XRCC5	9982	9883	9956	11167	10599	10873	12137	11800	12011	12342	12812	1.0	1.1	1.2	1.3
TMEM97	1399	1367	1340	1835	1697	1822	2389	2254	2284	2480	2545	1.0	1.3	1.7	1.8
NASP	1108	1222	1074	1555	1473	1460	1996	1990	2161	2318	2522	1.0	1.3	1.8	2.1
CCDC117	1835	1899	1892	2273	2347	2249	2832	2703	2914	2961	3346	1.0	1.2	1.5	1.7
TIFA	185	157	164	283	238	230	423	504	444	583	608	1.0	1.5	2.7	3.5
VRK1	380	348	369	545	524	450	811	843	822	987	939	1.0	1.4	2.3	2.6
XPO1	7335	7274	7180	8121	8055	7713	9547	9286	9554	9839	10293	1.0	1.1	1.3	1.4
ERLIN1	971	965	1008	1199	1239	1125	1572	1614	1566	1726	1877	1.0	1.2	1.6	1.8

XRCC2	162	113	149	247	230	230	412	494	476	545	595	1.0	1.7	3.3	4.0
TOPBP1	1946	1725	1757	2083	2173	2130	2709	2713	2741	2832	2912	1.0	1.2	1.5	1.6
KNTC1	456	444	456	632	612	594	944	919	838	881	974	1.0	1.4	2.0	2.1
C4orf19	895	824	810	1314	1281	1306	2345	2332	2168	2352	2408	1.0	1.5	2.7	2.8
NUDT3	1466	1564	1531	1755	1770	1807	2105	2078	2064	2080	2161	1.0	1.2	1.4	1.4
PCYOX1	3378	3434	3219	3676	3866	3812	4577	4478	4443	4390	4510	1.0	1.1	1.3	1.3
ANXA9	265	297	257	362	363	388	535	633	566	607	615	1.0	1.4	2.1	2.2
GGCT	2493	2741	2477	3018	3018	3086	3867	4030	3878	4301	4305	1.0	1.2	1.5	1.7
PEX11A	208	281	232	379	405	402	760	741	733	915	968	1.0	1.6	3.1	3.9
SSBP1	1779	1915	1762	2065	2093	2113	2429	2453	2468	2521	2561	1.0	1.1	1.3	1.4
GINS1	676	845	683	999	1014	1010	1868	1803	1930	2110	2178	1.0	1.4	2.5	2.9
PDCD4	1135	1275	1105	1542	1467	1532	2324	2505	2529	2689	2698	1.0	1.3	2.1	2.3
PHB2	3477	3816	3417	4332	4112	4423	5454	5430	5457	5785	6040	1.0	1.2	1.5	1.7
SLC39A8	1140	1293	1137	1464	1385	1461	2055	2073	2014	2132	2295	1.0	1.2	1.7	1.9
STARD10	5536	5338	5446	6336	5808	6052	7873	8028	7527	8795	8872	1.0	1.1	1.4	1.6
TST	1007	989	1061	1346	1273	1247	2172	2232	2165	2916	2748	1.0	1.3	2.1	2.8
TSPAN15	1512	1595	1468	1945	1870	1846	3129	3111	3000	3800	3687	1.0	1.2	2.0	2.5
ABHD2	5289	5087	5341	5604	5834	6016	11069	11314	10816	14020	14785	1.0	1.1	2.1	2.7
UGDH	14413	14109	14425	14879	15391	15100	21513	21502	21179	24707	25017	1.0	1.1	1.5	1.7
ELOVL5	2208	2205	2152	2486	2417	2515	4162	4340	4018	5191	5256	1.0	1.1	1.9	2.4
HMCN1	2661	2709	2714	3028	3085	2982	3863	3614	4370	4126	4286	1.0	1.1	1.5	1.6
SIVA1	2029	2070	2050	2245	2339	2282	2735	2706	2818	2887	3006	1.0	1.1	1.3	1.4
NUP160	2745	2733	2680	2897	3052	3051	3456	3474	3702	3604	3810	1.0	1.1	1.3	1.4
TYMS	750	769	632	843	949	873	1978	1795	1991	2020	1974	1.0	1.2	2.7	2.8
CDC45	287	339	268	388	438	391	833	716	896	980	909	1.0	1.4	2.7	3.2
RMI2	627	728	670	832	831	803	1205	1181	1232	1232	1164	1.0	1.2	1.8	1.8
RNASEH2A	423	533	482	633	581	611	1049	1041	1118	1067	1001	1.0	1.3	2.2	2.2
MASTL	441	395	411	542	573	572	1026	971	974	1105	1098	1.0	1.4	2.4	2.6
NUP205	2489	2442	2443	2787	2956	2813	4063	3934	4040	4340	4296	1.0	1.2	1.6	1.8
SKA3	261	224	240	298	376	333	754	692	719	779	822	1.0	1.4	3.0	3.3
HLTF	2906	2812	2806	3331	3488	3343	4778	4966	4645	4838	5050	1.0	1.2	1.7	1.7
NCAPD3	836	830	797	916	991	1009	1414	1369	1358	1412	1475	1.0	1.2	1.7	1.8
XRCC6	8250	8026	8085	8749	8927	8702	10308	10265	10033	10728	10630	1.0	1.1	1.3	1.3
EIF2AK4	1033	990	1011	1202	1185	1140	1626	1635	1564	1710	1598	1.0	1.2	1.6	1.6
YBX1	13224	13561	13273	14968	15013	15260	17988	18650	18348	19005	19135	1.0	1.1	1.4	1.4
CHAF1A	355	379	390	483	509	514	788	852	810	899	897	1.0	1.3	2.2	2.4
CHEK2	223	256	229	380	380	374	821	805	814	1001	923	1.0	1.6	3.4	4.1
MCM7	4986	5124	4965	7016	6805	6918	11451	11493	11294	13456	13075	1.0	1.4	2.3	2.6
TFF3	519	634	606	808	806	939	1842	2204	1869	2464	2468	1.0	1.5	3.4	4.2
MIS18BP1	538	591	615	748	715	731	1320	1436	1304	1415	1515	1.0	1.3	2.3	2.5
LRIG1	1109	1177	1185	1350	1300	1345	1761	1731	1747	1803	1870	1.0	1.2	1.5	1.6
CDCA5	987	941	856	1290	1261	1148	2708	2587	2726	2799	2960	1.0	1.3	2.9	3.1
**H19**	**621**	**545**	**514**	**985**	**1017**	**915**	**5016**	**5300**	**5274**	**6293**	**6407**	**1.0**	**1.7**	**9.3**	**11.3**
KNSTRN	360	320	326	464	412	422	935	905	903	932	1016	1.0	1.3	2.7	2.9
C6orf132	142	155	148	237	229	186	656	661	623	632	716	1.0	1.5	4.4	4.5
PRDX3	2081	1991	1882	2324	2323	2133	3665	3766	3368	3619	3895	1.0	1.1	1.8	1.9
LAPTM4A	8704	8883	8557	9738	9462	9533	13679	13816	13496	14752	14481	1.0	1.1	1.6	1.7
CKS1B	639	646	598	876	793	775	2042	2071	2009	2270	2221	1.0	1.3	3.2	3.6
ASF1B	729	763	715	967	824	910	2167	2186	2014	2186	2301	1.0	1.2	2.9	3.1
KIF22	2148	2205	2014	2589	2365	2450	4316	4127	4192	4251	4292	1.0	1.2	2.0	2.0
PLK1	1464	1367	1354	1643	1719	1641	4821	4691	4793	4910	4644	1.0	1.2	3.4	3.4
CENPF	818	845	781	983	1022	962	2777	2623	2874	2878	2967	1.0	1.2	3.4	3.6
BIRC5	587	548	528	726	702	685	1938	1784	1900	1890	1932	1.0	1.3	3.4	3.4
RACGAP1	1413	1384	1351	1601	1613	1560	2866	2791	2880	2771	2772	1.0	1.2	2.1	2.0
FANCI	1961	1891	1849	2147	2254	2041	5558	5181	5240	5280	5706	1.0	1.1	2.8	2.9
SMC4	2237	2282	2257	2625	2659	2433	5607	5636	5390	5467	5797	1.0	1.1	2.5	2.5
CEP55	494	516	500	639	676	636	1834	1729	1676	1709	1942	1.0	1.3	3.5	3.6
LIG1	1361	1409	1400	1673	1549	1660	3630	3361	3308	3504	3427	1.0	1.2	2.5	2.5
H2AFZ	8136	8398	8242	9097	8850	9177	17386	17013	16367	17303	17536	1.0	1.1	2.0	2.1
TPX2	1742	1787	1732	2127	1945	1898	5248	5135	5164	5290	5589	1.0	1.1	3.0	3.1
MYBL2	3011	3207	3000	3474	3299	3366	6366	6125	6417	6354	6498	1.0	1.1	2.1	2.1
CNNM2	2627	2549	2586	2757	2891	2949	5081	4786	4848	5483	5639	1.0	1.1	1.9	2.1
NT5C2	4278	4081	4242	4526	4715	4685	8731	8484	8250	9247	9855	1.0	1.1	2.0	2.3
CAV2	5380	5488	5344	5889	6075	6039	8119	8197	8129	8796	9054	1.0	1.1	1.5	1.7
DARS2	1079	1098	976	1235	1326	1306	2605	2669	2515	2829	2958	1.0	1.2	2.5	2.8
**AREG**	**108**	**105**	**144**	**196**	**263**	**251**	**2021**	**2194**	**1787**	**2864**	**3026**	**1.0**	**2.0**	**16.8**	**24.7**

ZWILCH	825	768	794	979	1055	1016	1956	1968	1862	2059	2225	1.0	1.3	2.4	2.7
MBNL1	3998	3998	4159	4296	4602	4612	6725	6635	6777	6379	6678	1.0	1.1	1.7	1.6
FIGN	174	140	175	250	223	271	731	848	781	842	891	1.0	1.5	4.8	5.3
BUB1B	276	353	335	440	431	469	1253	1239	1274	1214	1360	1.0	1.4	3.9	4.0
CENPA	234	292	248	353	409	384	998	972	1020	1043	1061	1.0	1.5	3.9	4.1
CCP110	901	904	908	1016	1070	1078	1790	1674	1739	1856	1862	1.0	1.2	1.9	2.1
KPNA2	10105	10056	9602	11167	11174	11176	17278	16324	16536	17517	17361	1.0	1.1	1.7	1.8
TIMELESS	1977	2060	2074	2463	2470	2411	3890	3786	3899	4179	4007	1.0	1.2	1.9	2.0
PWP1	2428	2405	2467	2794	2698	2814	3763	3491	3462	3750	3713	1.0	1.1	1.5	1.5
RELL1	342	338	377	484	453	488	942	862	810	863	864	1.0	1.3	2.5	2.5
RPLP0	70551	69416	68624	73967	71465	73187	84229	82016	82925	83224	82803	1.0	1.0	1.2	1.2
TRIP13	434	456	448	675	598	580	1240	1194	1228	1312	1395	1.0	1.4	2.7	3.0
RAD51	190	176	183	296	247	277	630	586	630	675	737	1.0	1.5	3.4	3.9
RPS24	22828	22235	22122	24267	23353	23903	27456	27485	27350	27513	28580	1.0	1.1	1.2	1.3
RPL4	53347	53902	52998	57075	55817	58113	66440	65116	64927	66826	68025	1.0	1.1	1.2	1.3
CDC25A	112	135	143	248	242	186	330	383	352	437	388	1.0	1.7	2.7	3.2
POLE2	62	73	74	150	128	95	209	205	251	273	268	1.0	1.8	3.2	3.9
TBCD	2443	2617	2612	2998	2826	2860	3263	3063	3245	3343	3187	1.0	1.1	1.2	1.3
HADH	767	821	779	970	963	945	1349	1277	1369	1563	1492	1.0	1.2	1.7	1.9
CCDC170	389	433	401	599	509	570	894	847	867	1052	1029	1.0	1.4	2.1	2.6
PCNA	1725	1762	1605	2597	2215	2405	4289	4098	4193	5043	4984	1.0	1.4	2.5	3.0
E2F1	380	389	398	547	516	572	943	778	848	1099	983	1.0	1.4	2.2	2.7
LMNB2	3985	4074	4120	4485	4393	4522	5337	5286	5565	5820	5692	1.0	1.1	1.3	1.4
DNMT1	3240	3358	3450	3896	3706	3788	4790	4674	5123	5294	5244	1.0	1.1	1.5	1.6
MCM5	935	1051	1053	1630	1535	1519	2422	2495	2430	2768	2736	1.0	1.5	2.4	2.7
WDHD1	371	428	411	719	642	621	1097	1172	1144	1309	1391	1.0	1.6	2.8	3.3
**IGF1R**	**16129**	**16488**	**17020**	**20928**	**20478**	**20748**	**24756**	**26115**	**25606**	**27935**	**27698**	**1.0**	**1.3**	**1.5**	**1.7**
**XBP1**	**5252**	**5386**	**5297**	**12974**	**12888**	**12757**	**34491**	**38839**	**33195**	**49168**	**50437**	**1.0**	**2.4**	**6.7**	**9.4**
**CTSD**	**6508**	**6982**	**6851**	**15405**	**14239**	**14082**	**35509**	**38612**	**34277**	**53525**	**53380**	**1.0**	**2.1**	**5.3**	**7.9**
PKIB	75	85	106	268	259	235	1056	1263	1138	1709	1947	1.0	2.9	13.0	20.6
SKP2	120	130	163	233	268	273	577	608	618	690	785	1.0	1.9	4.4	5.4
UHRF1	2931	3002	3006	3658	3662	3659	4621	4685	4609	5548	5621	1.0	1.2	1.6	1.9
BRCA2	201	221	217	303	345	334	645	627	567	792	828	1.0	1.5	2.9	3.8
CDCA4	1077	1139	1060	1302	1329	1365	1815	1803	1786	2127	2151	1.0	1.2	1.6	2.0
FBL	3851	4055	3983	4504	4658	4737	5457	5464	5562	6287	5786	1.0	1.2	1.4	1.5
POP7	1471	1457	1465	1706	1732	1654	2027	1988	2084	2305	2028	1.0	1.2	1.4	1.5
POLD1	671	663	671	970	863	866	1285	1207	1275	1457	1297	1.0	1.3	1.9	2.1
NDC1	838	824	810	1041	1091	1045	1636	1462	1534	1623	1600	1.0	1.3	1.9	2.0
UBE2T	509	491	469	680	690	660	1135	1102	1201	1253	1143	1.0	1.4	2.3	2.4
TTC8	644	643	625	798	762	789	1023	1019	1062	1086	1020	1.0	1.2	1.6	1.7
CHTF18	615	555	622	727	755	853	1094	1065	1039	1127	1049	1.0	1.3	1.8	1.8
THRAP3	3588	3513	3610	3933	3817	3969	4299	4220	4191	4441	4305	1.0	1.1	1.2	1.2
SSRP1	4233	4126	4287	4853	4851	4815	5444	5359	5245	5857	5724	1.0	1.1	1.3	1.4
MRPL49	3002	2880	2891	3339	3177	3257	3662	3848	3699	3932	3763	1.0	1.1	1.3	1.3
DHCR7	8668	8330	9022	9768	9480	9700	10758	11387	11089	12185	11557	1.0	1.1	1.3	1.4
MRPL4	2592	2864	2748	3088	3057	3101	2878	2935	2854	3145	3031	1.0	1.1	1.1	1.1
NXT1	666	743	708	862	863	971	729	814	812	924	972	1.0	1.3	1.1	1.3
CCDC85B	1745	1915	1829	2315	2319	2406	1841	1959	2123	2452	2294	1.0	1.3	1.1	1.3
QTRTD1	1736	1746	1765	2051	2069	2151	1829	1845	1981	2072	2064	1.0	1.2	1.1	1.2
C3orf70	49	65	59	118	122	130	75	83	130	129	113	1.0	2.2	1.7	2.1
CCNB1IP1	624	663	694	770	856	911	747	793	847	892	865	1.0	1.3	1.2	1.3
TRAPPC2L	2197	2397	2424	2562	2579	2788	2434	2610	2701	2613	2611	1.0	1.1	1.1	1.1
ZNF236	445	496	457	629	577	596	478	652	550	576	641	1.0	1.3	1.2	1.3
ILF3-AS1	498	525	525	608	701	615	564	583	606	610	682	1.0	1.2	1.1	1.3
REV3L	1716	1798	1736	2012	2101	1912	1851	1878	2011	1948	2026	1.0	1.1	1.1	1.1
KBTBD6	368	349	358	495	453	460	425	478	449	488	410	1.0	1.3	1.3	1.3
ORC2	1176	1215	1209	1497	1415	1381	1421	1420	1345	1485	1411	1.0	1.2	1.2	1.2
ATRIP	166	187	213	311	275	267	273	264	242	273	275	1.0	1.5	1.4	1.5
TP53	3538	3333	3562	4368	3989	4011	4122	4105	4022	4140	4075	1.0	1.2	1.2	1.2
PYGL	3571	3499	3683	4093	3948	3891	4019	3949	3926	4002	4022	1.0	1.1	1.1	1.1
FAM117B	449	561	500	676	681	636	682	657	691	722	601	1.0	1.3	1.3	1.3
ZNRD1	78	127	119	179	174	194	198	182	166	213	200	1.0	1.7	1.7	1.9
ADA	80	92	80	139	122	143	128	131	132	128	136	1.0	1.6	1.6	1.6
CSTF1	1894	1935	1902	2212	2188	2250	2085	2247	2127	2238	2238	1.0	1.2	1.1	1.2
MEIS1	82	117	101	188	184	176	154	197	173	194	195	1.0	1.8	1.7	1.9
EHMT2	1849	1958	1968	2254	2260	2295	2257	2204	2378	2534	2348	1.0	1.2	1.2	1.3

EEF2K	2276	2272	2235	2649	2628	2622	2616	2624	2689	2804	2595	1.0	1.2	1.2	1.2
RBM24	14	26	13	85	94	90	76	82	89	176	185	1.0	5.0	4.6	10.1
TRA2B	6074	6468	6083	7101	7001	7197	7051	6936	7066	7642	7541	1.0	1.1	1.1	1.2
SLC2A1	1622	1603	1712	4621	4449	4443	3893	3996	3906	6374	6278	1.0	2.7	2.4	3.8
SFXN2	186	159	173	730	700	695	561	572	563	1138	1077	1.0	4.1	3.3	6.4
C1orf226	99	91	103	413	452	423	282	301	310	676	649	1.0	4.4	3.0	6.8
TFAP4	200	204	218	445	503	450	400	380	434	600	564	1.0	2.2	2.0	2.8
SLC35F6	2711	2733	2821	4341	4306	4165	4224	3837	3831	5213	4925	1.0	1.6	1.4	1.8
ZNF703	2129	2340	2224	3771	3508	3587	3214	3278	3247	4380	4098	1.0	1.6	1.5	1.9
LTBP1	2466	2544	2507	3713	3580	3630	3357	3168	3460	4091	4174	1.0	1.5	1.3	1.6
CD320	623	646	673	866	849	913	879	832	859	1026	936	1.0	1.4	1.3	1.5
IMPDH2	4167	4054	4075	4989	4864	5149	4983	4773	4943	5273	5239	1.0	1.2	1.2	1.3
CCDC88C	1524	1490	1576	1729	1758	1754	1454	1564	1682	1832	1828	1.0	1.1	1.0	1.2
IFT57	1279	1315	1168	1433	1502	1435	1275	1127	1407	1541	1514	1.0	1.2	1.0	1.2
NIP7	1449	1548	1520	1942	1705	1877	1670	1612	1585	1900	1895	1.0	1.2	1.1	1.3
TRIM28	18167	18385	18783	21057	19886	20790	19599	19261	18961	20223	20474	1.0	1.1	1.0	1.1
RTN4RL1	556	554	566	804	695	666	608	626	620	711	695	1.0	1.3	1.1	1.3
TYSND1	1075	1059	1021	1442	1296	1343	1078	1137	1189	1363	1232	1.0	1.3	1.1	1.2
NHP2	6039	6446	6113	7225	6899	7156	6239	6295	6469	6935	6823	1.0	1.1	1.0	1.1
TRPC6	4	6	5	56	45	93	7	11	8	36	57	1.0	12.9	1.7	9.3
HCK	3	5	3	75	55	77	5	7	4	47	44	1.0	18.7	1.4	12.4
SLC7A5	5970	5904	6319	11305	11169	11275	6218	6415	5980	10127	9700	1.0	1.9	1.0	1.6
NOLC1	6575	6530	6334	9241	9184	9243	6365	6273	6401	8459	8449	1.0	1.4	1.0	1.3
FADS1	2744	2563	2679	3953	3810	3717	2890	2984	2897	3441	3517	1.0	1.4	1.1	1.3
CEBPZ	2486	2451	2370	2943	2916	2838	2461	2559	2576	2747	2850	1.0	1.2	1.0	1.1
FKBP4	13105	12798	12780	15511	15695	15494	13069	12980	12866	15466	15348	1.0	1.2	1.0	1.2
HSPD1	21850	20903	20312	25057	25815	25085	21960	20721	21147	24861	25603	1.0	1.2	1.0	1.2
PPAT	1405	1275	1366	1768	1785	1655	1382	1359	1364	1711	1643	1.0	1.3	1.0	1.2
DDX10	614	636	644	808	850	808	610	596	600	777	824	1.0	1.3	1.0	1.3
BRI3BP	2580	2654	2649	3375	3385	3271	2680	2730	2697	3228	3439	1.0	1.3	1.0	1.3
FOXC1	321	299	320	407	447	504	327	336	324	465	459	1.0	1.4	1.0	1.5
FLAD1	1614	1621	1630	1861	1851	1961	1655	1617	1663	1904	1842	1.0	1.2	1.0	1.2
FARSB	1513	1495	1461	1895	1887	1925	1691	1632	1595	1915	1994	1.0	1.3	1.1	1.3
PPIF	2319	2335	2324	3495	3733	3674	2830	2860	2881	3667	3841	1.0	1.6	1.2	1.6
UCK2	1842	1764	1710	2454	2428	2418	1991	1978	2057	2546	2507	1.0	1.4	1.1	1.4
OSGIN1	718	708	658	1268	1226	1174	941	872	925	1358	1448	1.0	1.8	1.3	2.0
PRMT5	3480	3281	3268	4404	4369	4315	3830	3729	3794	4304	4335	1.0	1.3	1.1	1.3
IVNS1ABP	3958	4030	3904	4651	4624	4651	4359	4039	4184	4651	4812	1.0	1.2	1.1	1.2
SERBP1	10130	10395	10336	11631	12147	11767	10995	10580	10797	12126	12263	1.0	1.2	1.0	1.2
ABCE1	3546	3398	3289	4152	4440	4186	3628	3586	3588	4011	4298	1.0	1.2	1.1	1.2
PUS7	2280	2149	1946	2598	2744	2687	2303	2219	2238	2757	2847	1.0	1.3	1.1	1.3
AARS	7817	7611	7620	8667	8825	8588	7876	8063	7927	9149	9000	1.0	1.1	1.0	1.2
CXCL12	1232	1220	1124	2483	2419	2418	1425	1361	1277	3298	3293	1.0	2.0	1.1	2.8
SLC9A3R1	6921	6999	7153	11355	10915	11174	8095	8200	8227	12517	11974	1.0	1.6	1.2	1.7
ELFN2	610	615	625	1279	1231	1231	700	784	740	1456	1298	1.0	2.0	1.2	2.2
MIR3615	961	1051	977	1624	1560	1607	1156	1156	1115	1921	1751	1.0	1.6	1.1	1.8
IL17RB	210	204	180	326	296	297	247	217	201	348	288	1.0	1.5	1.1	1.6
GPHN	700	645	650	906	912	961	814	691	740	965	921	1.0	1.4	1.1	1.4
GPC6	875	954	947	1217	1314	1126	1068	892	922	1133	1252	1.0	1.3	1.0	1.3
ZC3H8	473	469	456	640	599	645	576	418	522	608	623	1.0	1.3	1.1	1.3
NARS	3369	3252	3244	3652	3604	3538	3565	3314	3336	3614	3689	1.0	1.1	1.0	1.1
DDX39A	2840	2668	2697	3163	3009	3158	3072	2926	2890	3142	3152	1.0	1.1	1.1	1.2
TIMM9	782	755	754	964	884	1000	882	888	843	965	969	1.0	1.2	1.1	1.3
ST6GALNAC2	2316	2261	2365	3022	2810	3059	2972	2900	2778	3070	3161	1.0	1.3	1.2	1.3
FIBP	2565	2441	2506	2831	2712	2857	2795	2757	2763	2817	2879	1.0	1.1	1.1	1.1
PALB2	749	709	664	1012	936	953	950	908	885	992	1026	1.0	1.4	1.3	1.4
GTF3A	2255	2239	2142	2694	2621	2555	2559	2466	2561	2749	2770	1.0	1.2	1.1	1.2
NAT10	2356	2373	2375	3349	3429	3171	3077	3063	3029	3428	3576	1.0	1.4	1.3	1.5
TPD52L1	1139	1136	1101	3852	3928	3958	2983	2867	2920	4548	4942	1.0	3.5	2.6	4.2
CAV1	10901	11048	11280	18597	17948	18433	17304	16089	16819	19839	20098	1.0	1.7	1.5	1.8
**PDZK1**	**27**	**31**	**20**	**445**	**521**	**399**	**185**	**195**	**212**	**580**	**560**	**1.0**	**17.4**	**7.6**	**21.8**
C1QTNF6	1525	1598	1491	3995	3759	3915	3032	2871	2849	3996	3770	1.0	2.5	1.9	2.5
BZW2	4445	4335	4423	5004	5070	4995	4879	4885	4710	5100	5117	1.0	1.1	1.1	1.2
RNF223	315	283	318	835	796	839	705	750	617	1010	1000	1.0	2.7	2.3	3.3
TMPRSS3	13	12	17	109	87	118	66	80	52	156	162	1.0	7.6	4.8	11.5
ATIC	4212	3939	4256	5505	5320	5301	5308	5002	4875	5479	5593	1.0	1.3	1.2	1.3

OXSM	255	210	212	345	314	312	330	264	291	301	330	1.0	1.4	1.3	1.4
USP54	620	582	520	778	693	691	718	718	662	732	757	1.0	1.3	1.2	1.3
FH	1390	1397	1342	1721	1526	1565	1673	1548	1562	1596	1774	1.0	1.2	1.2	1.2
WDR35	295	288	312	503	389	437	391	427	395	442	597	1.0	1.5	1.4	1.7
DOC2A	385	389	385	549	502	517	473	454	464	478	551	1.0	1.4	1.2	1.3
TCFL5	1419	1454	1449	1740	1716	1654	1678	1633	1676	1652	1842	1.0	1.2	1.2	1.2
RPGRIP1L	142	141	142	217	226	197	178	211	190	193	237	1.0	1.5	1.4	1.5
NOS1AP	36	48	43	174	177	152	114	131	75	153	219	1.0	4.0	2.5	4.4
FAM217B	1249	1227	1238	1504	1582	1596	1470	1483	1488	1462	1643	1.0	1.3	1.2	1.3
HDDC2	1432	1481	1332	2134	2322	2172	1815	1944	1958	2238	2406	1.0	1.6	1.3	1.6
SRRM1	3107	3020	3053	3399	3557	3453	3355	3321	3305	3459	3576	1.0	1.1	1.1	1.1
DDX19A	1721	1695	1603	1963	1908	1937	1759	1757	1776	1812	1936	1.0	1.2	1.1	1.1
PRDX6	7705	7854	7389	8558	8191	8280	8019	7884	8131	8497	8385	1.0	1.1	1.0	1.1
CDH1	36859	35563	36943	39082	39381	39317	38462	38622	38701	44145	43866	1.0	1.1	1.1	1.2
MGAT5	4286	4356	4527	4992	5006	4934	4790	5108	4834	6510	6405	1.0	1.1	1.1	1.5
MLPH	7913	7795	7824	8719	8229	8335	8487	8321	7724	9662	9520	1.0	1.1	1.0	1.2
TOR3A	2951	2862	3007	3257	3402	3175	3053	3041	2997	3512	3498	1.0	1.1	1.0	1.2
IGFBP4	4029	3930	3921	5490	5389	5562	4357	4398	4402	7647	7313	1.0	1.4	1.1	1.9
TIPARP	609	578	619	930	908	934	774	789	784	1301	1359	1.0	1.5	1.3	2.2
ATP2C1	6866	6807	6597	7248	7191	7219	7121	6911	6958	7447	7682	1.0	1.1	1.0	1.1
SYNCRIP	9642	9842	9620	10171	10485	10558	10154	9957	9890	10617	11290	1.0	1.1	1.0	1.1
MAPT	643	685	710	820	838	795	897	806	771	1048	1060	1.0	1.2	1.2	1.6
PSMG1	1140	1106	1257	1441	1419	1365	1426	1386	1458	1653	1743	1.0	1.2	1.2	1.5
CELSR2	20184	19677	20569	25581	25580	24330	25614	26419	25915	33943	33830	1.0	1.2	1.3	1.7
HR	1235	1158	1243	1572	1566	1589	1728	1663	1678	2204	2342	1.0	1.3	1.4	1.9
FKBP5	1287	1310	1296	1644	1707	1617	1743	1601	1771	2274	2220	1.0	1.3	1.3	1.7
**PGR**	**111**	**157**	**167**	**419**	**382**	**375**	**466**	**538**	**461**	**1319**	**1409**	**1.0**	**2.7**	**3.4**	**9.4**
JARID2	1874	1981	1992	2356	2401	2323	2376	2379	2489	3029	3102	1.0	1.2	1.2	1.6
PFKFB3	7221	7351	7581	8562	9002	9016	8598	8651	8869	10196	10543	1.0	1.2	1.2	1.4
NFIC	2753	2854	2959	3257	3286	3352	3379	3246	3364	3888	3849	1.0	1.2	1.2	1.4
AEN	1904	1826	1941	2116	2106	2118	2069	1974	2130	2352	2297	1.0	1.1	1.1	1.2
COLGALT1	2194	2035	2260	2430	2502	2425	2408	2351	2382	2874	2708	1.0	1.1	1.1	1.3
DCTPP1	2695	2905	2713	3426	3329	3412	2955	3271	3372	3922	3555	1.0	1.2	1.2	1.3
ENTPD1	424	414	388	560	577	528	481	558	623	653	658	1.0	1.4	1.4	1.6
OVOL2	89	84	60	128	145	124	94	113	110	154	136	1.0	1.7	1.4	1.9
KNOP1	1551	1490	1452	1751	1765	1667	1606	1750	1694	1848	1745	1.0	1.2	1.1	1.2
INTS5	1074	1110	1064	1213	1319	1283	1233	1186	1180	1409	1285	1.0	1.2	1.1	1.2
MRPL15	1585	1756	1594	1843	2066	2115	1862	1902	1893	2212	2015	1.0	1.2	1.1	1.3
ZNHIT6	488	469	522	616	714	678	615	640	663	779	767	1.0	1.4	1.3	1.6
BRIX1	1030	1014	1037	1144	1288	1251	1219	1185	1262	1395	1401	1.0	1.2	1.2	1.4
NUP93	1135	1094	1152	1297	1410	1370	1335	1310	1392	1470	1410	1.0	1.2	1.2	1.3
MOCOS	256	277	238	347	354	346	361	327	304	441	460	1.0	1.4	1.3	1.8
URI1	1881	1901	1755	2022	2150	2115	2113	2017	1957	2247	2204	1.0	1.1	1.1	1.2
AHSA1	7020	6688	6669	7688	7862	7781	7436	7329	7194	8281	8105	1.0	1.1	1.1	1.2
WDR89	600	542	538	742	798	782	763	664	670	838	837	1.0	1.4	1.2	1.5
NEIL2	394	366	345	522	540	567	496	518	485	628	636	1.0	1.5	1.4	1.7
CNBP	13574	13507	13030	14594	14744	15073	14340	14193	14305	15220	15405	1.0	1.1	1.1	1.1
SFXN4	549	594	618	741	787	742	690	675	680	793	885	1.0	1.3	1.2	1.4
SGPP1	773	880	790	1092	1060	1066	954	1001	955	1274	1384	1.0	1.3	1.2	1.6
DNMBP	1923	1974	1931	2488	2566	2413	2332	2357	2414	2863	3052	1.0	1.3	1.2	1.5
ZNRF3	523	577	542	736	716	677	672	689	695	838	878	1.0	1.3	1.3	1.6
TELO2	2766	2809	2790	3371	3250	3428	3170	3154	3191	3781	3558	1.0	1.2	1.1	1.3
HSPB8	1026	1053	1122	2647	2684	2527	2278	2148	1960	4538	4757	1.0	2.5	2.0	4.4
TSKU	372	352	376	786	822	817	753	717	681	1228	1325	1.0	2.2	2.0	3.5
MTR	1333	1301	1380	1779	1719	1692	1622	1599	1621	1946	2016	1.0	1.3	1.2	1.5
GEMIN4	984	954	1019	1331	1279	1362	1137	1135	1134	1453	1517	1.0	1.3	1.2	1.5
ELP2	1975	1970	1999	2600	2445	2682	2313	2289	2352	2721	2877	1.0	1.3	1.2	1.4
RBM14	2694	2671	2696	3336	3157	3284	3093	3046	2946	3498	3467	1.0	1.2	1.1	1.3
TUFM	6983	7158	7104	7958	7860	7899	7538	7472	7448	8165	8172	1.0	1.1	1.1	1.2
CNKSR3	495	513	535	654	646	641	591	610	643	669	678	1.0	1.3	1.2	1.3
HEY2	39	35	54	131	135	119	68	77	102	134	156	1.0	3.0	1.9	3.4
PDPR	2003	1960	2175	2701	2610	2572	2390	2253	2564	2739	2840	1.0	1.3	1.2	1.4
USP37	641	705	673	902	874	785	756	750	811	896	891	1.0	1.3	1.1	1.3
OGFOD1	911	849	925	1119	1139	1101	999	1040	1068	1260	1092	1.0	1.3	1.2	1.3
TSEN54	2316	2280	2302	2801	2653	2612	2519	2510	2507	2822	2754	1.0	1.2	1.1	1.2
MRPS34	2909	2857	2883	3404	3357	3222	3215	3126	3030	3633	3369	1.0	1.2	1.1	1.2

DNASE1	3011	2953	2938	3500	3363	3437	3122	3234	3133	3552	3394	1.0	1.2	1.1	1.2
NHP2L1	2614	2712	2684	3473	3404	3410	3112	2948	2946	3543	3229	1.0	1.3	1.1	1.3
PRMT5-AS1	1686	1556	1718	2076	2150	1926	1737	1746	1914	2016	2170	1.0	1.2	1.1	1.3
THAP9-AS1	467	413	417	575	585	536	480	481	501	539	629	1.0	1.3	1.1	1.4
IVD	2968	2873	3051	3376	3188	3281	3151	3072	3290	3371	3466	1.0	1.1	1.1	1.2
ANKRD17	5467	5212	5420	6013	5992	5900	5798	5600	5886	6092	6464	1.0	1.1	1.1	1.2
TSEN2	357	275	324	459	406	469	383	341	403	512	511	1.0	1.4	1.2	1.6
KIAA0100	12080	11718	12289	12905	12834	12852	12611	12455	12510	12742	13005	1.0	1.1	1.0	1.1

**CLUSTER 2**	**Normalised Expression**											**Mean Expression Difference**			
	**MCF7**			**MCF7**			**Y537S**			**Y537S**		**MCF7**	**MCF7**	**Y537S**	**Y537S**
**Gene**	**Vehicle**			**Estrogen**			**Vehicle**			**Estrogen**		**Vehicle**	**Estroge**	**Vehicle**	**Estroge**
	**1**	**2**	**3**	**1**	**2**	**3**	**1**	**2**	**3**	**1**	**2**				
DFFA	1588	1447	1474	1776	1748	1968	1677	1644	1437	1766	1788	1.0	1.2	1.1	1.2
ERI3	1033	1070	1067	1236	1198	1303	1103	1146	1029	1145	1198	1.0	1.2	1.0	1.1
MPHOSPH10	1813	1725	1688	2028	2185	2125	1957	1849	1951	1981	2045	1.0	1.2	1.1	1.2
PIGW	599	665	585	747	866	789	701	664	687	772	793	1.0	1.3	1.1	1.3
CCDC80	36	39	26	61	84	83	56	48	52	72	81	1.0	2.2	1.5	2.3
ZBTB2	1086	1123	1105	1639	1518	1550	1409	1270	1170	1434	1533	1.0	1.4	1.2	1.3
AK4	1493	1511	1517	1961	1959	2109	1756	1666	1544	1911	2036	1.0	1.3	1.1	1.3
TSFM	900	933	932	1188	1183	1166	1065	1035	953	1073	1078	1.0	1.3	1.1	1.2
SNHG16	3349	3361	3317	4219	4231	4394	3843	3803	3972	4206	4224	1.0	1.3	1.2	1.3
**RET**	**2827**	**2863**	**2791**	**4242**	**4438**	**4497**	**3753**	**3478**	**3678**	**4120**	**4064**	**1.0**	**1.6**	**1.3**	**1.4**
STC2	3855	3828	3774	21474	21281	21007	9247	10029	9279	16947	17037	1.0	5.6	2.5	4.4
DLC1	534	496	548	928	900	935	719	692	718	798	777	1.0	1.8	1.3	1.5
LINC01016	1	1	2	102	113	106	12	33	21	73	51	1.0	82.0	16.9	47.5
EXOSC5	491	482	464	645	735	729	553	601	590	679	638	1.0	1.5	1.2	1.4
RRP1B	3806	3871	3717	4813	4785	4729	4060	4058	3997	4694	4546	1.0	1.3	1.1	1.2
PFAS	731	692	798	1191	1179	1202	823	882	816	1185	1103	1.0	1.6	1.1	1.5
**RARA**	**4841**	**4853**	**4884**	**6528**	**6665**	**6781**	**4975**	**5309**	**5325**	**6577**	**6372**	**1.0**	**1.4**	**1.1**	**1.3**
GPR132	80	70	106	223	253	216	142	124	105	225	228	1.0	2.7	1.4	2.7
STARD7	8302	8183	8339	9519	9642	9400	8778	8629	8894	9383	9331	1.0	1.2	1.1	1.1
CAD	5118	4842	5218	6567	6682	6623	5493	5328	5598	6589	6552	1.0	1.3	1.1	1.3
NCL	30986	30273	30781	39192	39947	39678	33698	32809	34518	40035	39645	1.0	1.3	1.1	1.3
NOP56	3826	3750	3893	5168	5382	5176	4293	4194	4446	5135	5182	1.0	1.4	1.1	1.3
PDCD5	1523	1347	1343	1567	1670	1654	1460	1484	1461	1563	1671	1.0	1.2	1.0	1.2
HLA-DRB5	44	29	23	61	71	76	32	46	37	66	59	1.0	2.2	1.2	1.9
NOP58	2399	2132	2133	2763	2825	2887	2416	2648	2515	2907	3021	1.0	1.3	1.1	1.3
TFRC	21605	21364	21313	24817	25828	25698	23204	24105	23068	26739	27369	1.0	1.2	1.1	1.3
C17orf97	75	65	64	111	122	129	81	121	77	113	125	1.0	1.8	1.4	1.8
POLR3C	1224	1165	1236	1350	1493	1503	1316	1459	1302	1512	1450	1.0	1.2	1.1	1.2
HEATR1	2925	2673	3193	3277	3477	3435	2990	2909	2881	3139	3204	1.0	1.2	1.0	1.1
CIRH1A	2149	2046	2183	2585	2549	2650	2114	2097	2180	2502	2339	1.0	1.2	1.0	1.1
PPFIA4	71	59	104	202	206	234	88	97	72	165	141	1.0	2.8	1.1	2.0
ILF3	13543	13357	13565	14779	14532	14731	13981	13579	13694	14278	14247	1.0	1.1	1.0	1.1
TTLL12	2444	2472	2517	2999	2980	3074	2678	2516	2542	2875	2751	1.0	1.2	1.0	1.1
GAPDHS	78	56	87	128	139	137	93	72	92	139	105	1.0	1.8	1.2	1.7
ZBTB24	483	488	510	564	722	641	555	522	511	560	557	1.0	1.3	1.1	1.1
MRPS35	1802	1774	1731	1911	2164	1995	1843	1855	1811	1904	2036	1.0	1.1	1.0	1.1
FASTKD2	1116	1060	1086	1171	1375	1378	1225	1221	1174	1300	1253	1.0	1.2	1.1	1.2
FAM136A	2986	2942	2895	3261	3269	3312	2848	3136	2983	3006	3358	1.0	1.1	1.0	1.1
HPDL	111	103	106	163	160	173	88	101	94	118	176	1.0	1.5	0.9	1.4
FAM86B1	79	62	89	123	142	118	67	110	83	87	115	1.0	1.7	1.1	1.3
CRTAP	2397	2590	2654	2844	2811	2881	2830	2727	2631	2748	2682	1.0	1.1	1.1	1.1
HLF	22	43	59	89	100	119	45	36	33	67	65	1.0	2.5	0.9	1.6
PMM2	2337	2420	2543	2709	2723	2772	2446	2444	2553	2599	2574	1.0	1.1	1.0	1.1
SETBP1	281	348	358	457	388	495	364	324	368	373	353	1.0	1.4	1.1	1.1
FRAT2	597	582	615	811	771	735	710	614	634	668	676	1.0	1.3	1.1	1.1
CLUAP1	260	231	244	331	356	330	302	264	287	273	275	1.0	1.4	1.2	1.1
SLC26A2	837	846	842	1148	1160	1124	933	846	917	805	845	1.0	1.4	1.1	1.0
PIK3R1	2108	2030	1959	2828	2908	2820	2065	2071	2182	2081	2284	1.0	1.4	1.0	1.1
NADSYN1	1736	1752	1858	2141	1958	2165	1985	1805	1904	1948	2063	1.0	1.2	1.1	1.1
SUPV3L1	559	567	609	804	679	727	693	604	654	669	737	1.0	1.3	1.1	1.2
ANKMY1	456	414	457	587	529	593	478	524	522	489	512	1.0	1.3	1.1	1.1
ALG1	911	925	937	1255	1223	1208	1022	1058	1062	1079	1049	1.0	1.3	1.1	1.2

POFUT1	1926	1822	1901	2262	2272	2261	1977	2030	2020	2129	2058	1.0	1.2	1.1	1.1
AFAP1L2	547	595	583	755	679	701	577	635	522	514	530	1.0	1.2	1.0	0.9
PGP	1631	1681	1795	2063	1954	2091	1595	1730	1474	1624	1549	1.0	1.2	0.9	0.9
CHERP	3108	3025	3171	3442	3590	3424	3014	3113	3149	3088	3124	1.0	1.1	1.0	1.0
RASL10B	208	182	239	301	292	297	159	192	219	208	219	1.0	1.4	0.9	1.0
CSRP2BP	659	588	668	803	783	736	638	651	698	655	646	1.0	1.2	1.0	1.0
FAM86C1	314	319	313	441	430	421	293	341	314	359	316	1.0	1.4	1.0	1.1
FAM86EP	110	88	98	195	181	165	85	116	102	119	100	1.0	1.8	1.0	1.1
DANCR	884	873	851	1197	1000	1045	888	883	916	940	846	1.0	1.2	1.0	1.0
IMP3	1734	1681	1883	2082	1949	1988	1737	1738	1703	1874	1701	1.0	1.1	1.0	1.0
SELO	1019	1090	1061	1244	1214	1228	1001	1052	1130	1158	1000	1.0	1.2	1.0	1.0
FAM46A	1093	1210	1152	1318	1351	1314	1179	1114	1064	1173	1229	1.0	1.2	1.0	1.0
NPIPB3	401	465	442	523	549	575	445	423	380	409	421	1.0	1.3	1.0	1.0
DLX1	439	456	488	599	612	549	505	459	454	530	488	1.0	1.3	1.0	1.1
SLC25A15	678	770	752	895	994	924	788	735	759	852	789	1.0	1.3	1.0	1.1
NLE1	685	716	733	919	944	867	704	644	709	729	669	1.0	1.3	1.0	1.0
NCR3LG1	17	32	31	79	63	71	19	13	17	30	35	1.0	2.6	0.6	1.2
TBL3	1498	1637	1673	2030	1882	1944	1452	1402	1573	1711	1562	1.0	1.2	0.9	1.0
LAP3	2784	3082	2938	3262	3390	3181	2829	2723	2785	3067	2878	1.0	1.1	0.9	1.0
HTT	8104	8065	8262	9170	9603	9138	7973	7974	8620	8709	8801	1.0	1.1	1.0	1.1
SRR	785	765	790	900	965	924	723	731	766	862	862	1.0	1.2	0.9	1.1
SRPRB	2796	2710	2766	3096	3263	3154	2669	2717	2778	3015	3077	1.0	1.2	1.0	1.1
MAP6D1	1031	984	902	1113	1333	1247	963	927	986	1133	1051	1.0	1.3	1.0	1.1
RRP1	2966	3190	2978	3551	3808	3773	2927	2928	3181	3738	3431	1.0	1.2	1.0	1.2
CD3EAP	1704	1623	1732	1937	2006	1963	1606	1493	1546	1954	1715	1.0	1.2	0.9	1.1
EMC1	1827	1675	1737	2039	1972	1995	1792	1644	1681	1937	1796	1.0	1.1	1.0	1.1
SLC25A19	281	290	284	403	410	428	304	270	268	403	320	1.0	1.5	1.0	1.3
IFRD2	1577	1581	1493	2146	2160	2050	1559	1434	1469	1950	1788	1.0	1.4	1.0	1.2
AKAP1	4773	4167	4511	5829	5726	5618	4261	4150	4442	4934	4933	1.0	1.3	1.0	1.1
GPATCH4	1637	1394	1530	1960	1888	1857	1453	1390	1398	1678	1655	1.0	1.3	0.9	1.1
PES1	5168	4838	4842	6028	6020	6177	4576	4518	4640	5262	5219	1.0	1.2	0.9	1.1
SNHG1	1613	1451	1457	1878	1861	2009	1464	1374	1390	1640	1541	1.0	1.3	0.9	1.1
QTRT1	667	604	653	797	769	768	579	576	597	709	639	1.0	1.2	0.9	1.1
POLR1A	4323	4070	4289	5256	5460	5280	3716	3807	4163	4654	4304	1.0	1.3	0.9	1.1
CLUH	4949	4960	5201	6520	6186	6336	4648	4756	4690	5715	5449	1.0	1.3	0.9	1.1
FARSA	3481	3438	3352	4116	3939	4064	3129	3089	3151	3734	3510	1.0	1.2	0.9	1.1
LONRF2	1789	1818	1798	2832	2881	2723	1369	1473	1467	2190	2057	1.0	1.6	0.8	1.2
IMP4	3567	3411	3317	4182	4248	4235	3350	3464	3269	3927	3836	1.0	1.2	1.0	1.1
ARHGAP26	867	751	813	1562	1558	1504	761	754	673	1190	1186	1.0	1.9	0.9	1.5
NOP2	2027	1895	1919	2580	2566	2431	1945	1955	1916	2259	2178	1.0	1.3	1.0	1.1
PLEKHH1	717	677	678	830	876	835	674	719	695	791	777	1.0	1.2	1.0	1.1
IPO5	4036	3712	3848	4437	4591	4252	3848	3887	3727	4182	4147	1.0	1.1	1.0	1.1
NOC3L	749	678	673	863	882	849	735	678	664	769	845	1.0	1.2	1.0	1.2
LARS	3577	3418	3512	3984	4086	4003	3463	3296	3416	3617	3898	1.0	1.1	1.0	1.1
UTP20	2644	2610	2633	3149	3509	3171	2695	2544	2580	2859	3137	1.0	1.2	1.0	1.1
ISG20L2	2875	2859	2801	3635	3611	3639	2732	2854	2801	3123	3079	1.0	1.3	1.0	1.1
PNPO	1197	1247	1206	1606	1589	1535	1161	1149	1216	1388	1337	1.0	1.3	1.0	1.1
SLC16A6	225	233	198	372	357	366	205	192	199	258	276	1.0	1.7	0.9	1.2
EIF3B	13674	13950	13650	16027	15853	16089	13351	12843	13437	14398	14173	1.0	1.2	1.0	1.0
PWP2	1878	1818	1825	2149	2229	2386	1780	1748	1828	2068	2048	1.0	1.2	1.0	1.1
IL1RAP	207	227	206	316	350	392	188	187	188	261	268	1.0	1.7	0.9	1.2
WDR3	2047	1887	1791	2904	2904	2665	1922	1775	1960	2523	2512	1.0	1.5	1.0	1.3
POLR1B	1874	1798	1744	2781	2743	2674	1924	1828	1989	2424	2262	1.0	1.5	1.1	1.3
AMD1	3631	3516	3435	4419	4699	4619	3620	3387	3724	4127	4358	1.0	1.3	1.0	1.2
WDR43	3318	3284	3232	4064	3959	4123	3135	2945	3097	3621	3697	1.0	1.2	0.9	1.1
MRTO4	1648	1641	1631	2062	2038	2124	1656	1503	1547	1918	1974	1.0	1.3	1.0	1.2
GPS2	4255	4082	4190	4834	4751	4840	4357	4031	4138	4502	4581	1.0	1.2	1.0	1.1
KARS	7697	7520	7711	8857	8900	9025	7758	7448	7384	8155	8311	1.0	1.2	1.0	1.1
POLR2A	6609	6434	6615	7118	7029	7097	6636	6502	6635	6765	6710	1.0	1.1	1.0	1.0
AHCY	7531	7320	7592	9751	9672	9502	7447	7194	7590	8213	7992	1.0	1.3	1.0	1.1
PLCD3	3813	3484	3787	4675	4348	4433	3659	3813	3802	4131	4078	1.0	1.2	1.0	1.1
METTL1	806	733	762	988	976	985	749	740	806	856	899	1.0	1.3	1.0	1.1
NBPF1	1809	1623	1662	2552	2343	2378	1622	1722	1692	1959	2130	1.0	1.4	1.0	1.2
TIGD1	331	276	332	451	445	460	314	317	305	355	401	1.0	1.4	1.0	1.2
GNL3	2040	1989	2004	2261	2352	2498	2054	2039	2065	2169	2275	1.0	1.2	1.0	1.1
C10orf2	1671	1614	1553	2051	2125	2223	1566	1621	1634	1958	1902	1.0	1.3	1.0	1.2

EIF5A	13481	13155	13211	15451	15307	15772	13321	13471	13624	14891	14875	1.0	1.2	1.0	1.1
HNRNPDL	3727	3756	3625	4579	4504	4772	3780	3764	3959	4193	4196	1.0	1.2	1.0	1.1
BOD1	2355	2467	2372	3870	3653	3858	2637	2710	2409	3141	3175	1.0	1.6	1.1	1.3
ADORA1	107	113	132	1041	1028	1023	104	112	116	391	394	1.0	8.8	0.9	3.3
SEMA3B	413	451	437	1400	1378	1404	406	502	465	829	824	1.0	3.2	1.1	1.9
RIMS4	1325	1402	1310	3027	2827	2852	1424	1358	1430	2082	2032	1.0	2.2	1.0	1.5
KCNK15	445	481	462	1111	1049	1074	518	510	469	794	722	1.0	2.3	1.1	1.6
FREM2	898	919	1025	1970	2160	2046	1119	1012	1166	1247	1474	1.0	2.2	1.2	1.4
TUBA1B	40282	40931	40741	44449	43775	45069	40569	40478	40888	41693	42719	1.0	1.1	1.0	1.0
FAM57A	762	790	801	1110	990	1046	687	767	776	925	963	1.0	1.3	0.9	1.2
RPF2	1099	1014	1089	1287	1320	1337	986	1025	1017	1250	1321	1.0	1.2	0.9	1.2
TMEM120B	2038	2002	2055	2678	2468	2533	1934	1896	1957	2546	2610	1.0	1.3	0.9	1.3
SEPT9	12716	12666	13352	14318	13833	14359	12300	12280	11906	13895	13359	1.0	1.1	0.9	1.1
CCT7	12385	12375	12284	13191	13162	13378	11937	12040	11783	13187	12824	1.0	1.1	1.0	1.1
KRT19	64112	68055	63882	77160	75698	77675	62979	69425	64613	71158	70435	1.0	1.2	1.0	1.1
TSR1	2274	2208	2355	2782	2882	2731	2112	2319	2085	2497	2593	1.0	1.2	1.0	1.1
U2SURP	2783	2831	2833	3272	3399	3356	2680	2806	2635	3126	3015	1.0	1.2	1.0	1.1
GOT2	2914	2995	3026	3296	3303	3206	2938	3040	2906	3146	3207	1.0	1.1	1.0	1.1
NBPF10	1681	1723	1743	1889	2058	1923	1367	1369	1625	1793	1843	1.0	1.1	0.8	1.1
XPOT	10452	10459	10395	12540	12753	12456	9351	9154	9222	10408	10888	1.0	1.2	0.9	1.0
WDR74	1818	1840	1892	2257	2292	2281	1552	1504	1647	1815	1976	1.0	1.2	0.8	1.0
PPRC1	2690	2595	2868	3199	3115	3185	2496	2357	2499	2658	2819	1.0	1.2	0.9	1.0
DDX46	4098	4155	4076	4439	4463	4439	3788	3674	3972	3931	4223	1.0	1.1	0.9	1.0
BCLAF1	6966	6896	7006	7427	7621	7315	6781	6636	7011	6883	7614	1.0	1.1	1.0	1.0
MGA	1684	1678	1744	1880	2099	2025	1441	1298	1542	1554	1560	1.0	1.2	0.8	0.9
WDR36	2016	1930	2019	2464	2615	2473	1586	1416	1609	1562	1744	1.0	1.3	0.8	0.8
PPARGC1B	327	328	399	418	564	526	242	209	229	251	237	1.0	1.4	0.6	0.7
AMOTL1	659	573	623	762	755	743	397	342	419	371	369	1.0	1.2	0.6	0.6
ACSS1	204	195	192	281	274	313	71	59	94	99	57	1.0	1.5	0.4	0.4
AMPD2	2714	2699	2887	3443	3269	3333	2313	2207	2256	2329	2259	1.0	1.2	0.8	0.8
PRDM6	293	291	328	396	387	420	253	191	246	245	236	1.0	1.3	0.8	0.8
DUS1L	3839	3639	3813	4319	3989	4123	3276	3348	3354	3506	3430	1.0	1.1	0.9	0.9
PISD	1664	1602	1699	1997	1828	1843	1344	1391	1398	1432	1464	1.0	1.1	0.8	0.9
SLC46A1	1041	1015	985	1311	1242	1221	752	822	842	881	880	1.0	1.2	0.8	0.9
SULT2B1	1210	1161	1232	1679	1483	1546	898	964	881	1048	1036	1.0	1.3	0.8	0.9
ANKRD13B	641	614	635	920	838	839	391	388	385	521	479	1.0	1.4	0.6	0.8
SLC12A9	4954	5161	5086	5514	5503	5447	4374	4405	4264	4590	4249	1.0	1.1	0.9	0.9
SLC25A39	11721	11778	11418	13262	12946	13010	10179	10234	10276	10767	10200	1.0	1.1	0.9	0.9
NOP16	3185	3223	3110	3714	3665	3822	2573	2568	2589	2875	2675	1.0	1.2	0.8	0.9
HAGHL	1319	1357	1284	1576	1551	1597	1059	1087	1064	1210	1121	1.0	1.2	0.8	0.9
GGA2	3813	3787	3778	4277	4134	4087	3219	3536	3422	3536	3331	1.0	1.1	0.9	0.9
SLC47A1	49	28	26	105	109	84	12	11	8	10	7	1.0	2.9	0.3	0.3
SLC24A3	611	604	617	825	861	866	336	390	376	362	423	1.0	1.4	0.6	0.6
PLXDC2	749	739	771	914	890	886	578	634	574	616	591	1.0	1.2	0.8	0.8
G3BP1	8477	8619	8482	9528	9540	9628	6665	6458	6350	7044	7462	1.0	1.1	0.8	0.9
MINA	1107	1177	1146	1374	1433	1418	782	743	776	883	934	1.0	1.2	0.7	0.8
RRP12	2698	2847	2952	3279	3396	3507	1879	2010	1914	2285	2285	1.0	1.2	0.7	0.8
LARP1	16462	16048	16632	17981	18664	18655	12469	12906	12421	13996	13869	1.0	1.1	0.8	0.9
SLC3A2	5662	5881	5936	6657	6850	7019	4147	3985	4047	4862	4707	1.0	1.2	0.7	0.8
RAN	19580	20024	19255	21475	21989	22009	16265	16023	16316	17267	17000	1.0	1.1	0.8	0.9
PGAM5	3874	3875	4016	4345	4288	4219	2898	2839	2921	3201	3211	1.0	1.1	0.7	0.8
HSPA4	8409	8155	8095	9174	9185	9042	4935	4888	4996	5628	5665	1.0	1.1	0.6	0.7
NAT8L	1714	1718	1778	1908	2046	1942	926	899	941	1194	1058	1.0	1.1	0.5	0.6
DHX37	2273	2170	2432	2632	2691	2700	1315	1354	1368	1560	1478	1.0	1.2	0.6	0.7
YARS	2706	2701	2704	2981	2887	3059	1927	1995	1968	2141	2255	1.0	1.1	0.7	0.8
LRRC59	9725	9718	9841	10276	10304	10387	8179	7840	8014	8345	8488	1.0	1.1	0.8	0.9
JAK1	8642	8265	8631	9591	9652	9549	6307	5956	6122	6660	7031	1.0	1.1	0.7	0.8
COL12A1	9699	10186	10718	12831	14077	13879	5187	4837	5475	5865	6499	1.0	1.3	0.5	0.6
ATAD3B	607	591	657	766	751	745	431	415	451	529	478	1.0	1.2	0.7	0.8
RCN1	956	865	973	1132	1147	1176	618	598	677	736	743	1.0	1.2	0.7	0.8
C3orf58	1274	1337	1347	1599	1571	1437	982	931	1041	1071	1096	1.0	1.2	0.7	0.8
ARRDC3	829	805	843	1000	937	987	615	543	523	615	637	1.0	1.2	0.7	0.8
MTHFD1L	2507	2474	2428	2838	2790	2878	1782	1687	1599	2019	2034	1.0	1.1	0.7	0.8
DDX42	7077	6683	6642	7668	7502	7557	5879	5527	5602	6342	6002	1.0	1.1	0.8	0.9
PPM1G	8164	7672	7830	8646	8437	8485	6879	6476	6716	7230	7232	1.0	1.1	0.8	0.9
PSME3	4723	4411	4423	5015	5149	5134	3893	3769	3747	4072	4043	1.0	1.1	0.8	0.9

RHOBTB3	4945	4912	4616	5653	5362	5092	3310	3329	3176	3271	3717	1.0	1.1	0.7	0.7
SLC38A2	26420	26149	26308	27712	27565	27523	15274	15121	15239	16261	16401	1.0	1.0	0.6	0.6
ODC1	2595	2692	2584	2972	2960	3200	1293	1259	1246	1262	1325	1.0	1.2	0.5	0.5
COL18A1	14371	15190	15611	16588	15796	16669	10697	10760	11004	11385	10949	1.0	1.1	0.7	0.7
CS	9103	8921	9175	9821	9535	9558	7646	7722	7480	7806	7589	1.0	1.1	0.8	0.8
RPS26	7178	7071	7209	7784	7666	7657	5722	5756	5560	5673	5652	1.0	1.1	0.8	0.8
KPNB1	13103	13038	12895	13777	13966	13916	11126	10674	10573	11125	10943	1.0	1.1	0.8	0.8
TAF9	1728	1698	1641	1854	1887	1974	1147	1214	1175	1197	1196	1.0	1.1	0.7	0.7
EIF4B	25469	26885	25680	28851	27848	28192	25020	25187	25225	24674	25114	1.0	1.1	1.0	1.0
KCNJ8	87	112	84	152	135	165	75	72	64	56	60	1.0	1.6	0.7	0.6
SLC38A1	15858	15699	15522	16423	16730	16909	15110	15301	15619	15291	15297	1.0	1.1	1.0	1.0
TMEM186	1902	1816	1942	2520	2617	2774	1691	1766	1788	1935	1964	1.0	1.4	0.9	1.0
PCYOX1L	562	563	566	797	810	877	469	476	494	547	593	1.0	1.5	0.9	1.0
NRCAM	10862	11092	11136	12345	12135	12074	10677	10735	10640	10748	11041	1.0	1.1	1.0	1.0
INHBB	3162	3154	3171	4412	4385	4438	2896	2836	2897	2937	3056	1.0	1.4	0.9	0.9
ZNF552	169	163	182	250	280	234	164	162	145	170	190	1.0	1.5	0.9	1.0
C14orf132	760	877	837	1103	1102	1106	687	792	677	850	862	1.0	1.3	0.9	1.0
NME1	2000	2114	2100	2639	2645	2579	1898	1948	1911	2153	2268	1.0	1.3	0.9	1.1
YTHDF1	6289	6453	6260	6831	6811	6907	6124	6151	6034	6334	6534	1.0	1.1	1.0	1.0
CYP2U1	403	350	374	509	468	460	312	371	312	327	396	1.0	1.3	0.9	1.0
CHCHD4	485	453	451	600	567	584	430	457	432	462	527	1.0	1.3	0.9	1.1
SMG7	8286	7658	8159	8577	8707	8770	7491	7337	7339	7950	7953	1.0	1.1	0.9	1.0
CAP2	981	862	933	1117	1140	1161	744	656	739	845	900	1.0	1.2	0.8	0.9
KCTD17	734	649	723	873	811	848	605	542	612	721	618	1.0	1.2	0.8	1.0
UBAP2L	11135	10549	11079	11967	12026	11862	10547	10187	10016	10706	10678	1.0	1.1	0.9	1.0
HSP90AB1	72722	70800	73298	78122	79650	79971	69130	65933	67128	70730	71412	1.0	1.1	0.9	1.0
EFTUD2	3104	3067	3171	3643	3553	3544	2978	2888	2773	2944	3017	1.0	1.1	0.9	1.0
SKIV2L2	1673	1676	1728	2113	2107	1986	1501	1415	1342	1495	1600	1.0	1.2	0.8	0.9
SLC19A1	6111	5854	6142	7323	6995	7130	5726	5552	5359	5948	5747	1.0	1.2	0.9	1.0
SCARB1	2851	2721	2771	4377	4312	4120	2256	2296	2126	2709	2674	1.0	1.5	0.8	1.0
PYCR1	3484	3298	3434	4075	4100	4047	2957	2935	2838	3486	3236	1.0	1.2	0.9	1.0
ABAT	24066	22900	24507	35944	34864	35076	19043	18552	18803	23924	23373	1.0	1.5	0.8	1.0
GEMIN5	2384	2316	2401	3231	3277	3287	1885	1822	1926	2320	2280	1.0	1.4	0.8	1.0
MYBBP1A	2809	2907	2901	3656	3570	3680	2410	2408	2380	2907	2744	1.0	1.3	0.8	1.0
XYLT2	1164	1188	1216	1414	1432	1466	1103	1020	1089	1195	1132	1.0	1.2	0.9	1.0
AK2	2438	2281	2212	2627	2951	2773	2077	2065	2036	2221	2266	1.0	1.2	0.9	1.0
MCCC2	3369	3266	3157	4206	3915	4205	2485	2485	2477	2768	2945	1.0	1.3	0.8	0.9
NPM1	48087	46914	46321	57635	57700	58156	40225	39640	39024	43741	45438	1.0	1.2	0.8	0.9
LRPPRC	8891	8882	8639	10287	10240	10345	7811	7349	7482	8135	8204	1.0	1.2	0.9	0.9
RHOU	536	562	558	905	910	823	365	351	358	409	429	1.0	1.6	0.6	0.8
KRT13	111	138	165	1291	1268	1145	18	33	18	64	64	1.0	8.9	0.2	0.5
LSG1	3400	3330	3261	3946	3938	3847	3122	2860	2980	3212	3173	1.0	1.2	0.9	1.0
CHDH	890	841	839	1105	1113	1010	715	690	689	791	810	1.0	1.3	0.8	0.9
CPXM2	35	31	28	150	120	87	6	2	8	25	25	1.0	3.8	0.2	0.8
DDX49	2004	1951	1911	2301	2373	2311	1729	1631	1724	1905	1906	1.0	1.2	0.9	1.0
SRSF1	14423	14011	13980	16308	16302	15565	13418	13207	13218	14142	14747	1.0	1.1	0.9	1.0
BMS1	3815	3827	3789	4144	4128	4107	3742	3597	3612	3850	3938	1.0	1.1	1.0	1.0
PA2G4	5383	4823	5097	6732	7128	6823	4836	4517	4405	5517	5521	1.0	1.4	0.9	1.1
TOMM40	4626	4534	4720	5515	5378	5398	4232	4248	3880	4751	4904	1.0	1.2	0.9	1.0
NOC2L	5052	4998	5087	5768	5783	5805	4567	4782	4467	5283	5346	1.0	1.1	0.9	1.1
NLN	1509	1505	1416	1659	1754	1764	1288	1271	1214	1477	1530	1.0	1.2	0.9	1.0
ZNF239	215	156	198	273	315	261	172	147	153	155	206	1.0	1.5	0.8	1.0
PNN	4543	4207	4596	4972	4991	4954	4375	4095	4294	4375	4462	1.0	1.1	1.0	1.0
SNHG17	405	418	384	494	481	553	307	290	264	369	315	1.0	1.3	0.7	0.8
C14orf79	1089	1082	1005	1247	1243	1366	993	937	948	1085	1006	1.0	1.2	0.9	1.0
C1orf233	1222	1186	1087	1444	1357	1583	1022	1032	974	1094	1059	1.0	1.3	0.9	0.9
SF1	9336	8908	9316	9972	9512	10185	8669	8728	8595	9147	8759	1.0	1.1	0.9	1.0
PRKAR1B	2233	2245	2285	2728	2414	2603	2098	2085	2018	2204	2076	1.0	1.1	0.9	0.9
E2F4	2852	2984	2906	3222	3233	3365	2874	2669	2690	3069	2849	1.0	1.1	0.9	1.0
KRT15	204	214	176	431	363	412	154	166	148	239	180	1.0	2.0	0.8	1.1
ARMC6	1595	1623	1491	1883	1836	1904	1426	1407	1348	1731	1562	1.0	1.2	0.9	1.0
SMPD2	424	447	454	549	568	549	378	420	389	457	398	1.0	1.3	0.9	1.0
ANKRD16	289	294	313	387	400	387	232	271	260	301	255	1.0	1.3	0.9	0.9
ISG20	262	219	261	345	430	392	152	223	185	256	224	1.0	1.6	0.8	1.0
KMO	244	242	268	339	404	338	183	202	191	243	214	1.0	1.4	0.8	0.9
URB1	2990	2907	3150	3361	3701	3380	2611	2682	2742	2983	2824	1.0	1.2	0.9	1.0

IRS2	206	249	234	337	290	333	159	201	160	217	194	1.0	1.4	0.8	0.9
FAM207A	1692	1953	1968	2310	2206	2216	1419	1600	1538	1878	1744	1.0	1.2	0.8	1.0
MANEAL	1737	1666	1735	2227	2237	2246	1208	1358	1223	1642	1649	1.0	1.3	0.7	1.0
SLC22A5	1238	1119	1237	1671	1576	1588	722	784	736	1036	1067	1.0	1.3	0.6	0.9
C16orf45	1490	1497	1532	1870	1721	1769	1004	1071	1056	1408	1413	1.0	1.2	0.7	0.9
APRT	9111	8922	8850	9847	9522	9743	8397	8441	8266	9058	8970	1.0	1.1	0.9	1.0
CBFA2T3	3428	3354	3489	4139	3845	3981	2923	3016	3096	3487	3492	1.0	1.2	0.9	1.0
SDAD1	2195	2212	2171	2502	2540	2591	1877	1868	1974	2177	2112	1.0	1.2	0.9	1.0
HNRNPAB	14868	15301	15043	16334	16536	16603	13069	13240	13573	14579	14193	1.0	1.1	0.9	1.0
MATK	369	401	422	652	677	626	223	252	254	370	374	1.0	1.6	0.6	0.9
ATAD3A	1324	1406	1353	1594	1645	1626	1095	1074	1110	1375	1184	1.0	1.2	0.8	0.9
AIMP2	1624	1717	1570	1934	1941	1916	1325	1394	1408	1695	1523	1.0	1.2	0.8	1.0
FAM208B	6658	6546	7023	7254	7979	7490	5625	5511	5631	6011	6413	1.0	1.1	0.8	0.9
MACF1	2605	2567	2541	2778	3231	2998	1738	1949	2005	2133	2340	1.0	1.2	0.7	0.9
EBNA1BP2	2576	2499	2503	2783	2923	2861	2053	2145	2135	2451	2399	1.0	1.1	0.8	1.0
C15orf48	101	89	76	151	158	143	31	33	33	66	56	1.0	1.7	0.4	0.7
ARL10	615	627	662	777	814	794	474	502	469	562	552	1.0	1.3	0.8	0.9
CCDC137	1663	1671	1715	1915	2008	1892	1353	1364	1357	1539	1531	1.0	1.2	0.8	0.9
PUS1	1613	1669	1699	2073	2125	2013	1268	1296	1228	1579	1473	1.0	1.2	0.8	0.9
SRSF2	9203	9467	9350	10100	10110	10591	7717	7346	7805	8719	8750	1.0	1.1	0.8	0.9
NKAIN1	150	206	195	317	304	297	108	96	94	189	191	1.0	1.7	0.5	1.0
TLN2	430	438	466	655	674	604	373	286	305	408	417	1.0	1.4	0.7	0.9
TGIF2	2156	2134	2237	2730	2543	2552	1856	1580	1731	2065	2091	1.0	1.2	0.8	1.0
THOC7	6112	6392	6073	6604	6864	6883	5889	5572	5378	5777	6107	1.0	1.1	0.9	1.0
PRSS23	10179	9846	10010	10787	10905	10461	6662	6351	6643	9681	9693	1.0	1.1	0.7	1.0
NOP14-AS1	2813	2588	2651	2925	2950	3031	1961	2004	1774	2543	2513	1.0	1.1	0.7	0.9
LRRN2	219	202	183	272	308	277	87	77	68	166	152	1.0	1.4	0.4	0.8
STRAP	6364	6130	6199	6761	6877	6688	5444	5413	5112	6094	5848	1.0	1.1	0.9	1.0
GTPBP4	5638	5576	5476	5820	6115	6173	5030	4905	5051	5841	5584	1.0	1.1	0.9	1.0

**CLUSTER 3**	**Normalised Expression**											**Mean Expression Difference**			
	**MCF7**			**MCF7**			**Y537S**			**Y537S**		**MCF7**	**MCF7**	**Y537S**	**Y537S**
**Gene**	**Vehicle**			**Estrogen**			**Vehicle**			**Estrogen**		**Vehicle**	**Estroge**	**Vehicle**	**Estroge**
	**1**	**2**	**3**	**1**	**2**	**3**	**1**	**2**	**3**	**1**	**2**				
MAP3K6	1067	1010	1047	859	882	826	1071	991	1155	1080	995	1.0	0.8	1.0	1.0
DNAL1	619	590	644	505	554	484	587	564	632	646	576	1.0	0.8	1.0	1.0
IL10RB	382	352	413	326	300	319	435	356	398	430	397	1.0	0.8	1.0	1.1
RAB8B	922	886	852	720	705	743	951	985	937	898	894	1.0	0.8	1.1	1.0
EDEM1	1508	1521	1385	1037	1047	1094	1645	1650	1494	1498	1430	1.0	0.7	1.1	1.0
SEMA4C	4982	4948	4952	4058	3888	3916	5143	5406	5143	5031	4991	1.0	0.8	1.1	1.0
SGPL1	4985	5026	5087	4165	4151	4254	5389	5354	5453	5001	4913	1.0	0.8	1.1	1.0
GTF2IRD1	2110	2187	2185	1570	1513	1565	2407	2471	2395	2232	2136	1.0	0.7	1.1	1.0
LMNA	15595	16094	16072	13211	13227	13275	16335	16708	16527	15669	15560	1.0	0.8	1.0	1.0
CAPN1	11052	10740	10920	9043	8961	8991	11629	11746	11379	10921	10279	1.0	0.8	1.1	1.0
ARHGEF16	994	1055	1045	823	818	878	1168	1138	1057	1073	1037	1.0	0.8	1.1	1.0
TMEM41B	1147	1154	1125	773	835	840	1288	1277	1192	1196	1193	1.0	0.7	1.1	1.0
ERRFI1	5114	5231	5145	2914	3182	3305	5616	6099	5425	5862	6070	1.0	0.6	1.1	1.2
MAFF	407	428	397	263	279	245	467	470	453	459	465	1.0	0.6	1.1	1.1
KLF6	4222	4448	4419	2976	2873	3195	4552	4470	4675	4711	4320	1.0	0.7	1.0	1.0
LDLR	6553	6344	6855	5078	5192	5350	7017	7102	7096	6898	7025	1.0	0.8	1.1	1.1
ARHGAP32	1286	1266	1300	1142	1031	1108	1328	1372	1379	1333	1307	1.0	0.9	1.1	1.0
GJB3	196	168	176	133	118	119	208	195	212	210	200	1.0	0.7	1.1	1.1
INO80C	589	561	528	461	481	439	676	584	615	629	588	1.0	0.8	1.1	1.1
ACOX1	4578	4621	4549	3686	3810	3876	5272	5139	5380	4799	4712	1.0	0.8	1.1	1.0
CLDN4	12043	12357	12058	8521	8727	9043	15668	16389	16426	14125	13335	1.0	0.7	1.3	1.1
ALAS1	984	978	957	722	800	804	1103	1141	1223	1085	1010	1.0	0.8	1.2	1.1
GDPD3	179	159	158	119	116	133	215	189	214	188	159	1.0	0.7	1.2	1.0
KANK2	5025	5101	5072	4247	4137	4038	5774	5886	5785	5529	5361	1.0	0.8	1.1	1.1
CRABP2	14725	14630	14809	12056	12080	12246	16700	17342	16334	16018	15595	1.0	0.8	1.1	1.1
TMUB1	1761	1762	1751	1484	1401	1486	2035	2203	2017	2042	1782	1.0	0.8	1.2	1.1
RAB27B	3715	3824	3606	2417	2586	2575	4857	5168	4727	3965	4129	1.0	0.7	1.3	1.1
SPTSSB	78910	80245	75627	64219	65231	63470	90299	89861	89165	79440	81746	1.0	0.8	1.1	1.0
SCCPDH	6384	6637	5998	5075	4967	5045	8503	8413	8364	7092	7504	1.0	0.8	1.3	1.2
B4GALT4	2111	2059	2101	1609	1548	1548	2797	3005	2821	2415	2412	1.0	0.8	1.4	1.2
APOBEC3B	144	168	162	94	108	102	266	256	294	166	174	1.0	0.6	1.7	1.1
PLEKHG6	281	294	279	210	243	232	387	357	410	316	305	1.0	0.8	1.4	1.1

EMP1	128	204	150	91	101	135	283	273	315	224	194	1.0	0.7	1.8	1.3
YPEL3	1145	1232	1219	959	815	983	1477	1442	1470	1072	1131	1.0	0.8	1.2	0.9
ATP2A3	4678	4828	4970	4346	3785	4142	5639	5724	5548	4697	4554	1.0	0.8	1.2	1.0
CABLES2	1227	1206	1219	976	930	935	1412	1457	1539	1239	1241	1.0	0.8	1.2	1.0
HPS3	5419	5147	5478	4244	4293	4110	7416	7230	7600	5265	5479	1.0	0.8	1.4	1.0
CP	1032	1015	990	774	742	752	1460	1459	1414	1010	1128	1.0	0.7	1.4	1.1
SLC44A2	8671	8674	8539	7226	7186	7280	10332	10131	10231	8749	8780	1.0	0.8	1.2	1.0
CCNG2	2694	2820	2882	2215	2258	2155	3420	3544	3365	2719	2705	1.0	0.8	1.2	1.0
IDI2-AS1	858	827	836	729	697	668	1132	1142	1081	1020	1090	1.0	0.8	1.3	1.3
PPARG	926	792	828	609	625	619	1576	1493	1485	1250	1479	1.0	0.7	1.8	1.6
S100A16	11792	11075	11324	8915	8941	9019	16898	17689	16220	16181	16019	1.0	0.8	1.5	1.4
RTKN2	648	678	660	473	480	475	1224	1293	1139	1273	1287	1.0	0.7	1.8	1.9
NR4A1	1849	2024	1968	1346	1245	1301	4393	4612	4446	3912	3866	1.0	0.7	2.3	2.0
CDC42EP4	1187	1203	1276	1064	989	1008	1931	1946	1837	1834	1763	1.0	0.8	1.6	1.5
TC2N	1271	1214	1323	1016	975	1049	2549	2637	2081	2168	2252	1.0	0.8	1.9	1.7
CCNF	1182	1233	1210	925	1057	1010	1904	1798	1921	1892	1909	1.0	0.8	1.6	1.6
CEP70	196	264	222	162	180	185	448	389	429	392	389	1.0	0.8	1.9	1.7
PKP1	944	914	1044	718	798	720	1896	2086	2000	1753	1650	1.0	0.8	2.1	1.8
KIAA1217	889	832	883	712	744	693	1378	1492	1443	1265	1334	1.0	0.8	1.7	1.5
KRT83	210	218	185	117	173	99	1337	1578	1391	896	959	1.0	0.6	7.0	4.5
NYNRIN	230	273	244	205	181	204	1101	984	1029	885	817	1.0	0.8	4.2	3.4
CLSTN2	1103	1113	1144	904	926	950	4275	4482	4249	3501	3879	1.0	0.8	3.9	3.3
S100P	951	914	880	751	779	763	4917	5500	4991	4640	4572	1.0	0.8	5.6	5.0
TNFRSF1A	2893	2796	2789	2411	2323	2309	4835	5036	4724	4893	5027	1.0	0.8	1.7	1.8
CAPS	921	865	861	737	683	664	2767	3262	2996	2824	2774	1.0	0.8	3.4	3.2
KRT81	30865	32334	32587	23590	24147	22943	98739	104040	98906	95835	93644	1.0	0.7	3.1	3.0
USP30	663	579	633	497	561	498	821	861	815	784	738	1.0	0.8	1.3	1.2
HSH2D	139	118	119	83	92	69	168	209	160	171	160	1.0	0.6	1.4	1.3
S100A14	17916	17418	17107	14213	14553	14394	20941	22926	21742	21903	21687	1.0	0.8	1.3	1.2
CASD1	896	886	825	685	753	718	991	1065	1021	1005	1010	1.0	0.8	1.2	1.2
FAM114A1	990	923	1003	703	742	778	1326	1253	1380	1149	1203	1.0	0.8	1.4	1.2
PLEKHA2	922	902	897	718	781	768	1313	1135	1250	1109	1145	1.0	0.8	1.4	1.2
NCEH1	830	771	819	605	652	713	1097	1112	996	1042	992	1.0	0.8	1.3	1.3
CAPN5	313	332	340	252	255	252	538	516	485	449	410	1.0	0.8	1.6	1.3
KYNU	2705	2858	2767	2051	2106	2062	4175	4204	4041	3817	3780	1.0	0.7	1.5	1.4
SPDEF	4923	4936	4764	3748	3717	3851	6807	7126	6865	6252	6148	1.0	0.8	1.4	1.3
LYPD3	1836	1868	1773	1277	1224	1269	3077	3068	3077	2682	2558	1.0	0.7	1.7	1.4
C2CD4A	131	199	168	110	100	103	318	320	284	273	302	1.0	0.6	1.9	1.7
AHR	2410	2506	2525	1845	1908	2019	3397	3554	3206	3213	3297	1.0	0.8	1.4	1.3
DUSP6	786	786	797	447	483	469	1442	1468	1328	1341	1355	1.0	0.6	1.8	1.7
KLF5	1199	1199	1235	920	967	958	1665	1672	1621	1509	1611	1.0	0.8	1.4	1.3
CAPG	980	1056	1013	733	840	808	1341	1420	1410	1270	1356	1.0	0.8	1.4	1.3
GPR87	1600	1858	1695	1091	1188	1265	2342	2382	2273	2209	2185	1.0	0.7	1.4	1.3
GDF15	976	1035	1084	732	781	832	1360	1514	1374	1311	1305	1.0	0.8	1.4	1.3
ZFP36	911	873	897	798	712	688	1158	1154	1233	1220	1211	1.0	0.8	1.3	1.4
ZNF750	144	167	149	94	70	69	293	220	263	259	243	1.0	0.5	1.7	1.6
PTPN12	3833	3750	3726	3230	3087	3055	4330	4306	4437	4020	4217	1.0	0.8	1.2	1.1
ARNT	2486	2469	2482	2165	2047	2098	2882	2931	2798	2739	2789	1.0	0.8	1.2	1.1
TMPRSS13	485	472	432	398	394	357	690	673	589	601	540	1.0	0.8	1.4	1.2
ELF3	569	539	495	459	448	418	843	859	741	600	649	1.0	0.8	1.5	1.2
ABTB1	577	508	529	458	403	411	793	727	731	600	537	1.0	0.8	1.4	1.1
CMYA5	324	308	348	275	257	235	533	494	548	420	384	1.0	0.8	1.6	1.2
RAP1GAP	437	363	437	286	310	255	717	709	675	497	435	1.0	0.7	1.7	1.1
BRAF	452	532	536	435	419	387	705	694	721	575	641	1.0	0.8	1.4	1.2
TACSTD2	9155	10132	9920	8428	7960	8234	12211	12391	12603	10707	10461	1.0	0.8	1.3	1.1
FLOT1	1044	1159	1095	879	923	897	1675	1733	1727	1456	1513	1.0	0.8	1.6	1.4
NTN4	976	1002	905	678	722	708	1714	1917	1828	1364	1469	1.0	0.7	1.9	1.5
TMEM150C	1434	1574	1411	1249	1140	1199	2375	2374	2474	1842	2001	1.0	0.8	1.6	1.3
IKZF2	186	176	199	171	126	129	565	614	649	412	375	1.0	0.8	3.3	2.1
TMPRSS4	175	192	175	146	108	137	190	240	223	211	244	1.0	0.7	1.2	1.3
ZFYVE19	601	543	612	526	497	460	590	666	671	635	642	1.0	0.8	1.1	1.1
MARVELD3	1470	1407	1410	1231	1094	1161	1647	1630	1517	1493	1580	1.0	0.8	1.1	1.1
MFAP3L	360	306	332	279	243	243	416	467	376	381	393	1.0	0.8	1.3	1.2
ARHGAP27	383	353	451	290	271	275	532	464	485	456	478	1.0	0.7	1.2	1.2
ACP6	793	919	877	696	599	604	1088	1058	1050	905	986	1.0	0.7	1.2	1.1
DTX2	636	631	660	542	475	508	818	761	799	658	758	1.0	0.8	1.2	1.1

SEMA6C	247	206	303	163	129	171	431	438	440	328	339	1.0	0.6	1.7	1.3
CHST11	1059	1021	1139	909	881	925	1305	1382	1441	1208	1248	1.0	0.8	1.3	1.1
BRI3	1304	1278	1412	1137	1066	1198	1473	1624	1482	1311	1437	1.0	0.9	1.1	1.0
KREMEN1	643	614	655	491	469	539	911	1087	914	771	844	1.0	0.8	1.5	1.3
PRKCH	1375	1289	1317	1046	1045	1171	1582	1701	1602	1375	1602	1.0	0.8	1.2	1.1
NAB2	3782	3828	3742	3201	3137	3159	3503	3846	3675	4402	4164	1.0	0.8	1.0	1.1
ITGA6	985	1031	1040	795	860	815	1031	1144	1076	1243	1348	1.0	0.8	1.1	1.3
SLC44A3	155	159	161	118	102	121	201	242	146	188	214	1.0	0.7	1.2	1.3
S100A2	1137	1087	1050	766	867	765	1218	1586	1113	1276	1323	1.0	0.7	1.2	1.2
SGCB	695	650	718	472	569	549	801	854	818	846	789	1.0	0.8	1.2	1.2
SLC25A18	155	144	167	84	94	120	183	245	190	197	191	1.0	0.6	1.3	1.3
SOX9	811	670	832	540	616	655	803	894	893	880	938	1.0	0.8	1.1	1.2
ZNF365	832	782	800	507	573	678	958	938	847	894	916	1.0	0.7	1.1	1.1
CSGALNACT2	859	859	871	697	696	756	934	857	902	917	948	1.0	0.8	1.0	1.1
IFT88	552	470	472	400	422	426	533	474	436	428	579	1.0	0.8	1.0	1.0
SP4	394	421	350	324	313	309	437	413	413	344	398	1.0	0.8	1.1	1.0
ZG16B	340	360	305	278	261	276	402	415	316	313	359	1.0	0.8	1.1	1.0
C5orf49	59	65	46	38	22	32	54	51	55	26	66	1.0	0.5	0.9	0.8
PLK3	180	224	198	157	118	134	191	210	180	184	219	1.0	0.7	1.0	1.0
CHMP1B	2807	2954	2858	2524	2230	2538	2948	3051	2808	2704	2852	1.0	0.8	1.0	1.0
PBRM1	1771	1806	1943	1546	1552	1503	1863	1918	2028	1750	1760	1.0	0.8	1.1	1.0
DMTN	484	468	524	368	373	365	507	559	610	496	484	1.0	0.7	1.1	1.0
SECISBP2L	953	920	933	807	837	716	969	1031	1024	887	941	1.0	0.8	1.1	1.0
CCDC83	202	151	215	152	125	115	180	224	229	155	167	1.0	0.7	1.1	0.9
RND3	2802	2869	2756	2327	2368	2353	3051	3030	2929	2392	2607	1.0	0.8	1.1	0.9
DDIT4	3206	3232	3065	2642	2650	2635	3647	3722	3684	2672	2630	1.0	0.8	1.2	0.8
FAM171B	591	513	506	422	382	454	593	662	636	398	488	1.0	0.8	1.2	0.8
ABHD4	816	746	788	606	563	677	965	1064	975	679	745	1.0	0.8	1.3	0.9
TM4SF18	77	80	98	40	40	55	105	155	118	60	55	1.0	0.5	1.5	0.7
MARK2	4067	4104	4104	3561	3364	3553	3981	4264	4132	3914	3860	1.0	0.9	1.0	0.9
NAGK	3152	3170	2986	2683	2472	2594	3176	3374	3235	2979	2834	1.0	0.8	1.1	0.9
RAB11B-AS1	257	263	263	222	192	194	249	287	284	239	224	1.0	0.8	1.0	0.9
EPB41L5	2416	2282	2259	1883	1831	1778	2402	2411	2337	2065	2097	1.0	0.8	1.0	0.9
HEXIM1	2724	2709	2621	2112	2124	2042	2680	2893	2699	2424	2388	1.0	0.8	1.0	0.9
S100A9	509	480	503	388	354	353	498	515	497	447	475	1.0	0.7	1.0	0.9
KHNYN	3520	3548	3653	2950	2890	2880	3722	3636	3761	3372	3411	1.0	0.8	1.0	0.9
TTC9	760	795	795	577	632	529	848	895	850	757	696	1.0	0.7	1.1	0.9
PITPNM1	1382	1527	1492	1135	1135	1102	1453	1677	1495	1455	1269	1.0	0.8	1.1	0.9
RBBP8NL	371	378	301	278	273	256	442	445	406	322	299	1.0	0.8	1.2	0.9
ETV4	172	178	148	132	113	108	184	206	204	182	131	1.0	0.7	1.2	0.9
EFEMP1	7573	7434	7416	5910	5746	5531	8593	8245	8405	6549	6511	1.0	0.8	1.1	0.9
PGPEP1	700	728	665	525	518	503	788	808	837	679	608	1.0	0.7	1.2	0.9
TM4SF1	1102	1162	1106	380	428	460	1339	1365	1391	781	675	1.0	0.4	1.2	0.6
CLDN9	655	620	653	500	436	495	701	697	675	556	495	1.0	0.7	1.1	0.8
MSRB1	1133	1156	1053	943	834	892	1116	1122	1107	991	928	1.0	0.8	1.0	0.9
EZR-AS1	475	512	516	320	332	384	483	513	619	530	406	1.0	0.7	1.1	0.9
CASZ1	1487	1582	1633	1140	1042	1185	1552	1641	1760	1400	1430	1.0	0.7	1.1	0.9
UBALD2	1148	1159	1213	759	707	860	1157	1152	1279	1027	1040	1.0	0.7	1.0	0.9
CEBPB	3210	3348	3563	2768	2478	2899	3482	3664	3626	3251	3003	1.0	0.8	1.1	0.9
TTYH3	4766	4916	5118	4186	4013	4228	5026	5030	5143	4650	4322	1.0	0.8	1.0	0.9
SNN	1636	1531	1373	1230	1171	1209	1492	1555	1638	1281	1319	1.0	0.8	1.0	0.9
DDX59	615	556	546	477	413	453	556	595	698	493	547	1.0	0.8	1.1	0.9
TMEM54	1551	1591	1595	1417	1289	1335	1552	1540	1557	1387	1495	1.0	0.9	1.0	0.9
GATA2	1534	1623	1546	1415	1186	1336	1626	1576	1610	1308	1373	1.0	0.8	1.0	0.9
HDAC5	454	623	531	406	411	445	634	588	543	455	442	1.0	0.8	1.1	0.8
EPOR	100	115	145	78	83	79	153	137	124	80	69	1.0	0.7	1.1	0.6
CLEC3A	573	639	527	450	385	371	540	507	521	234	257	1.0	0.7	0.9	0.4
BMF	1274	1383	1378	1097	974	1047	1603	1447	1392	765	757	1.0	0.8	1.1	0.6
HPCA	474	497	503	409	393	432	468	499	478	392	388	1.0	0.8	1.0	0.8
FAM20C	2772	2873	2967	2467	2257	2398	2743	2784	2865	2168	2039	1.0	0.8	1.0	0.7
SEMA3E	2210	2304	2232	1880	1887	1936	2176	2289	2270	1630	1733	1.0	0.8	1.0	0.7
MAP2	908	907	891	753	719	721	870	881	954	721	667	1.0	0.8	1.0	0.8
SP2	1141	1126	1184	979	903	934	1157	1216	1261	920	941	1.0	0.8	1.1	0.8
VAT1L	95	75	72	54	42	60	77	116	95	54	35	1.0	0.6	1.2	0.5
STX5	1465	1368	1408	1215	1138	1234	1349	1465	1299	1255	1186	1.0	0.8	1.0	0.9
SLC26A10	333	291	317	238	242	263	260	310	261	267	254	1.0	0.8	0.9	0.8

DDA1	1461	1320	1532	1247	1173	1258	1341	1482	1435	1361	1221	1.0	0.9	1.0	0.9
PPP1R18	144	131	163	94	108	112	116	130	97	127	89	1.0	0.7	0.8	0.7
DENND1C	599	535	562	398	406	396	429	545	482	519	392	1.0	0.7	0.9	0.8
BIN1	299	266	256	220	207	199	199	248	227	223	180	1.0	0.8	0.8	0.7
NME7	910	881	832	720	757	721	712	834	755	770	732	1.0	0.8	0.9	0.9
RTN2	593	615	588	503	431	417	575	562	604	562	465	1.0	0.8	1.0	0.9
PVRL2	6486	6647	6358	5498	5254	5301	5619	6088	6190	5743	5443	1.0	0.8	0.9	0.9
PI4K2B	1563	1640	1491	1306	1273	1260	1448	1450	1540	1352	1356	1.0	0.8	0.9	0.9
VPS37D	181	216	202	131	154	137	155	195	240	169	146	1.0	0.7	1.0	0.8
SNHG18	55	60	38	28	29	29	40	62	57	46	35	1.0	0.6	1.0	0.8
MESDC1	2277	2731	2729	2178	2106	2136	2430	2680	2721	2715	2417	1.0	0.8	1.0	1.0
CHIC2	176	221	221	165	142	149	162	165	199	191	174	1.0	0.7	0.8	0.9
RAB43	207	222	207	140	132	184	178	171	213	214	169	1.0	0.7	0.9	0.9
MOB4	631	676	623	457	495	540	603	612	638	610	614	1.0	0.8	1.0	1.0
KCTD5	2361	2558	2416	1883	2041	2147	2319	2290	2225	2318	2295	1.0	0.8	0.9	0.9
PKNOX1	1007	936	952	831	805	821	918	892	932	902	912	1.0	0.8	0.9	0.9
MEAF6	1770	1710	1654	1517	1407	1436	1646	1597	1667	1669	1666	1.0	0.8	1.0	1.0
VSIG10L	206	205	242	150	113	127	171	203	193	184	207	1.0	0.6	0.9	0.9
BHLHE40-AS1	123	103	110	94	56	63	95	96	81	70	81	1.0	0.6	0.8	0.7
ARNTL	332	302	337	296	242	237	295	294	274	296	284	1.0	0.8	0.9	0.9
DYRK1B	974	1047	1149	928	896	860	1027	1117	1119	955	952	1.0	0.8	1.0	0.9
CYP4B1	135	141	164	116	94	105	152	197	153	118	114	1.0	0.7	1.1	0.8
ITPKA	46	57	93	47	28	33	50	73	66	40	60	1.0	0.6	1.0	0.8
ZNF254	492	484	578	427	387	369	539	481	501	440	487	1.0	0.8	1.0	0.9
TRIOBP	186	226	242	168	163	149	216	209	179	165	180	1.0	0.7	0.9	0.8
EMP3	117	146	163	90	93	96	143	151	132	111	134	1.0	0.7	1.0	0.9
PPARD	795	852	928	655	624	645	827	796	851	769	719	1.0	0.7	1.0	0.9
DNAJB1	8251	8501	8500	7197	7156	7200	8059	8295	8599	7787	7889	1.0	0.9	1.0	0.9
GIPC1	9205	9596	9471	7977	7777	7911	8994	9126	9092	8868	8746	1.0	0.8	1.0	0.9
RAB20	420	528	459	342	343	374	409	439	424	344	384	1.0	0.8	0.9	0.8
SIPA1	161	184	145	118	102	127	142	178	134	110	119	1.0	0.7	0.9	0.7
ARFGAP3	691	805	679	624	623	594	652	680	631	585	613	1.0	0.8	0.9	0.8
MARCH5	2508	2676	2363	2096	2118	2131	2418	2374	2210	2173	2375	1.0	0.8	0.9	0.9
MB	369	350	325	238	260	230	329	378	273	245	300	1.0	0.7	0.9	0.8
SLC4A2	10750	10588	11073	8726	8225	8306	9782	10122	9758	8541	8449	1.0	0.8	0.9	0.8
LGALS8	3069	3069	3219	2628	2599	2561	2983	3012	2923	2614	2723	1.0	0.8	1.0	0.9
KIAA1522	8941	9032	9279	7385	7210	6966	8668	8582	8672	7175	7431	1.0	0.8	1.0	0.8
PDPK1	4452	4342	4451	3810	3779	3603	4142	4153	4146	3657	3671	1.0	0.8	0.9	0.8
BCL2L1	4733	4654	4710	3953	3918	3892	4478	4487	4501	3792	3873	1.0	0.8	1.0	0.8
ADAM10	4839	4759	4595	3635	3634	3533	4237	4322	4170	3561	3638	1.0	0.8	0.9	0.8
CLMN	458	408	443	323	287	275	340	358	403	291	318	1.0	0.7	0.8	0.7
AKAP9	3628	3705	3803	3061	3013	3012	3592	3569	3790	3159	3242	1.0	0.8	1.0	0.9
MSX2	3067	3065	3048	2261	2079	2037	2983	2887	2857	2383	2384	1.0	0.7	1.0	0.8
METRNL	5180	5220	5193	4270	4183	4161	4943	4931	4903	4489	4334	1.0	0.8	0.9	0.8
LFNG	3299	3490	3612	3068	2775	2875	3350	3327	3332	2921	2953	1.0	0.8	1.0	0.8
EDN1	651	787	760	469	506	538	647	599	641	480	494	1.0	0.7	0.9	0.7
FAM110C	1140	1218	1217	779	799	767	945	968	975	671	719	1.0	0.7	0.8	0.6
ITGB6	26370	26181	26405	17479	18643	18398	22619	22068	22241	16636	17048	1.0	0.7	0.8	0.6
SMTN	6947	7227	7128	5034	5029	5059	5950	5639	6046	4726	4780	1.0	0.7	0.8	0.7
SEPT10	515	577	565	389	394	372	472	443	454	367	365	1.0	0.7	0.8	0.7
SLC9A3R2	2909	3053	3027	2372	2341	2495	2669	2852	2613	2334	2382	1.0	0.8	0.9	0.8
ATP2C2	977	1070	974	738	714	716	845	867	811	713	761	1.0	0.7	0.8	0.7
TSC22D2	4781	4868	4887	3965	4023	4048	4267	4358	4336	3932	3940	1.0	0.8	0.9	0.8
OXTR	2709	2828	2854	1398	1462	1405	2030	2061	1947	1540	1481	1.0	0.5	0.7	0.5
CRIP2	11815	12472	11924	9566	9266	9600	10412	10961	10941	9538	9238	1.0	0.8	0.9	0.8
SOX13	2421	2387	2466	1981	1860	2036	2236	2202	2339	1949	2014	1.0	0.8	0.9	0.8
CCDC68	365	336	367	191	184	233	278	261	277	216	232	1.0	0.6	0.8	0.6
EDARADD	280	255	284	175	148	177	200	215	198	154	169	1.0	0.6	0.7	0.6
MLLT11	729	663	732	543	470	532	606	631	620	558	560	1.0	0.7	0.9	0.8
ZFYVE1	1233	1250	1268	926	872	915	1084	1154	1065	947	1019	1.0	0.7	0.9	0.8
JUNB	8151	9114	9185	5512	5530	6068	7271	7523	7568	7169	6473	1.0	0.6	0.8	0.8
BAIAP2L1	2713	2804	2868	1926	2133	2225	2605	2508	2499	2341	2316	1.0	0.7	0.9	0.8
BCL3	1031	1093	1165	772	789	889	994	921	964	908	818	1.0	0.7	0.9	0.8
GYLTL1B	195	189	189	143	135	162	170	167	177	168	153	1.0	0.8	0.9	0.8
RIT1	1212	1250	1201	978	930	1029	1179	1090	1151	1052	990	1.0	0.8	0.9	0.8
C1orf106	1606	1504	1530	1112	1070	1143	1400	1245	1360	1256	1229	1.0	0.7	0.9	0.8

FERMT2	2790	2741	2773	2059	2064	2163	2612	2416	2466	2299	2275	1.0	0.8	0.9	0.8
IRF2BPL	2976	3453	3422	2583	2655	2782	3263	3201	3193	2788	2584	1.0	0.8	1.0	0.8
GAS2L1	2415	2625	2562	2108	2086	2056	2368	2434	2499	2257	2099	1.0	0.8	1.0	0.9
PTPRJ	1956	1988	2008	1582	1681	1639	2078	1961	2053	1626	1605	1.0	0.8	1.0	0.8
MYO6	5072	5221	4965	4235	4324	4285	5251	4865	5221	4369	4620	1.0	0.8	1.0	0.9
ARHGAP29	538	560	615	399	460	416	520	517	571	416	459	1.0	0.7	0.9	0.8
KIAA1462	1246	1198	1256	846	940	898	1102	1075	1211	940	892	1.0	0.7	0.9	0.7
PDGFB	2388	2403	2531	1965	1974	2077	2385	2408	2294	2133	2131	1.0	0.8	1.0	0.9
LIPH	566	565	579	328	339	364	534	541	516	379	396	1.0	0.6	0.9	0.7
SSH3	3534	3324	3488	2851	2711	2780	3541	3362	3339	2944	2943	1.0	0.8	1.0	0.9
IQGAP1	18985	18009	19148	15016	15409	14964	18891	18519	18477	16553	17018	1.0	0.8	1.0	0.9
SPNS2	515	533	494	323	323	320	505	502	432	386	424	1.0	0.6	0.9	0.8
DDAH2	1692	1783	1587	1330	1312	1294	1655	1638	1624	1473	1455	1.0	0.8	1.0	0.9
GAB1	841	892	788	642	626	611	782	815	762	723	683	1.0	0.7	0.9	0.8
CSGALNACT1	1436	1426	1374	923	1016	1001	1251	1421	1265	975	1029	1.0	0.7	0.9	0.7
PLD2	608	550	524	413	408	413	536	520	512	428	399	1.0	0.7	0.9	0.7
S100A10	40473	40372	38343	33343	33239	33321	38406	37189	36359	34375	34704	1.0	0.8	0.9	0.9
BAD	861	993	856	789	695	794	729	921	828	831	858	1.0	0.8	0.9	0.9
PTS	378	354	337	252	297	300	269	347	307	274	339	1.0	0.8	0.9	0.9
OSTM1	1133	1158	1154	800	973	879	957	1154	927	984	993	1.0	0.8	0.9	0.9
SPRY4	370	412	356	239	278	278	309	369	297	363	327	1.0	0.7	0.9	0.9
PHLDA1	4194	4633	4674	2720	3083	3134	3517	3549	3543	4018	4176	1.0	0.7	0.8	0.9
CORO1A	503	474	554	367	371	387	446	481	384	483	469	1.0	0.7	0.9	0.9
LGALS8-AS1	168	172	158	121	106	111	122	129	116	135	141	1.0	0.7	0.7	0.8
TAF13	2299	2236	2246	1847	1876	1952	2056	2133	2005	1975	2004	1.0	0.8	0.9	0.9
VASP	5179	5178	5223	3985	3951	4183	4583	4734	4617	4469	4370	1.0	0.8	0.9	0.9
CAPN2	7532	7497	7382	5698	5677	5820	6335	6694	6451	6223	6299	1.0	0.8	0.9	0.8
OVOL1	1483	1471	1475	1183	1165	1174	1332	1275	1204	1206	1259	1.0	0.8	0.9	0.8
FAM214A	2109	2139	2068	1751	1696	1694	2008	1955	1775	1794	1830	1.0	0.8	0.9	0.9
ARNTL2	1165	1408	1360	989	1066	911	1065	1106	1150	1094	1142	1.0	0.8	0.8	0.9
MAP1S	906	957	1048	747	715	734	837	884	848	803	818	1.0	0.8	0.9	0.8
TMC7	400	468	476	341	340	346	387	383	357	387	380	1.0	0.8	0.8	0.9
OTUD1	1080	1154	1251	871	841	963	970	1009	929	909	916	1.0	0.8	0.8	0.8
PTPRE	1138	1134	1175	755	828	927	878	916	886	932	873	1.0	0.7	0.8	0.8
STX1A	1072	996	1083	791	860	899	948	950	857	868	921	1.0	0.8	0.9	0.9
GCLM	743	842	838	608	696	726	697	701	671	647	725	1.0	0.8	0.9	0.8
PANK2	707	771	789	607	721	593	688	861	655	783	691	1.0	0.8	1.0	1.0
SAMD8	555	536	553	462	494	412	515	620	519	444	560	1.0	0.8	1.0	0.9
CAB39L	281	249	261	197	233	185	266	319	252	232	272	1.0	0.8	1.1	1.0
CCDC114	90	97	84	57	68	51	100	93	84	82	73	1.0	0.6	1.0	0.9
SPAG7	972	926	986	823	838	744	989	926	949	909	840	1.0	0.8	1.0	0.9
DUSP5	1002	974	1017	526	577	516	848	891	786	867	789	1.0	0.5	0.8	0.8
MEF2D	1279	1266	1263	1069	1091	1011	1253	1168	1178	1137	1209	1.0	0.8	0.9	0.9
CDK17	1638	1751	1757	1387	1500	1376	1703	1517	1556	1647	1584	1.0	0.8	0.9	0.9
MKNK1	574	516	503	395	474	443	504	499	507	484	457	1.0	0.8	0.9	0.9
LCOR	945	934	987	621	842	741	903	958	921	934	905	1.0	0.8	1.0	1.0
TMTC2	864	858	879	692	812	687	887	875	955	846	928	1.0	0.8	1.0	1.0
STAM2	1218	1194	1218	977	1074	1025	1245	1266	1279	1200	1200	1.0	0.8	1.0	1.0
ATP11B	2518	2531	2443	2057	2222	2016	2604	2588	2617	2508	2520	1.0	0.8	1.0	1.0
TMEM87B	1933	1985	2030	1604	1678	1676	2085	2194	2064	1872	2060	1.0	0.8	1.1	1.0
KTN1	9807	9617	9694	8197	8361	8162	9737	10083	9453	9237	9832	1.0	0.8	1.0	1.0
WDR47	1491	1412	1538	1136	1167	1096	1515	1534	1500	1359	1513	1.0	0.8	1.0	1.0
TP53BP2	1427	1356	1420	1122	1171	1206	1403	1417	1458	1289	1325	1.0	0.8	1.0	0.9
KIAA0232	2931	2931	2913	2360	2557	2525	2905	2887	2990	2802	2754	1.0	0.8	1.0	0.9
TEAD3	1552	1469	1504	1116	1185	1218	1574	1567	1564	1466	1504	1.0	0.8	1.0	1.0
USP6NL	2685	2615	2606	2065	2160	2167	2588	2628	2636	2524	2519	1.0	0.8	1.0	1.0

**CLUSTER 4**	**Normalised Expression**											**Mean Expression Difference**			
	**MCF7**			**MCF7**			**Y537S**			**Y537S**		**MCF7**	**MCF7**	**Y537S**	**Y537S**
**Gene**	**Vehicle**			**Estrogen**			**Vehicle**			**Estrogen**		**Vehicle**	**Estroge**	**Vehicle**	**Estroge**
	**1**	**2**	**3**	**1**	**2**	**3**	**1**	**2**	**3**	**1**	**2**				
STK40	1513	1510	1568	1239	1230	1155	1352	1293	1313	1220	1172	1.0	0.8	0.9	0.8
NOTCH3	15108	14993	15793	12779	12453	11856	13332	13505	12863	12078	11552	1.0	0.8	0.9	0.8
TCF7L2	1193	1083	1245	946	908	891	992	922	1011	920	772	1.0	0.8	0.8	0.7
KIAA0226	2257	2096	2296	1906	1794	1741	2007	1963	2024	1809	1699	1.0	0.8	0.9	0.8
PPP1R13L	2132	2240	2272	1861	1722	1783	1860	1938	1952	1805	1581	1.0	0.8	0.9	0.8

MIDN	7175	7566	8024	5983	5732	6142	6196	6471	6514	6039	5675	1.0	0.8	0.8	0.8
ID2	1297	1303	1271	1111	982	1100	1105	1111	1142	991	923	1.0	0.8	0.9	0.7
BSDC1	1567	1536	1576	1345	1229	1411	1415	1413	1428	1250	1181	1.0	0.9	0.9	0.8
SPIRE2	1227	1358	1211	817	801	857	990	1056	1099	670	636	1.0	0.7	0.8	0.5
RAB3D	3428	3249	3347	2443	2317	2441	2826	2702	2839	1919	2009	1.0	0.7	0.8	0.6
YPEL2	1448	1404	1442	1212	1166	1172	1279	1232	1367	989	1112	1.0	0.8	0.9	0.7
SMIM5	163	218	191	111	115	119	114	134	128	73	95	1.0	0.6	0.7	0.4
**IL1R1**	**673**	**772**	**864**	**526**	**483**	**588**	**563**	**567**	**589**	**328**	**342**	**1.0**	**0.7**	**0.7**	**0.4**
LXN	5439	5512	5463	4530	4440	4482	5112	5289	4598	3977	4256	1.0	0.8	0.9	0.8
SERPINB1	845	961	864	674	593	672	740	755	669	573	567	1.0	0.7	0.8	0.6
ATP1B1	12853	13076	12995	8642	8982	9020	9493	10307	9163	8462	8574	1.0	0.7	0.7	0.7
PLCB3	4673	4550	4685	3776	3724	3851	3853	4112	3843	3714	3564	1.0	0.8	0.8	0.8
CYTH2	2262	2222	2177	1644	1584	1683	1833	1841	1788	1633	1569	1.0	0.7	0.8	0.7
GJB2	327	287	300	149	187	215	211	228	189	153	159	1.0	0.6	0.7	0.5
NEK7	2137	2098	2091	1655	1792	1744	1783	1899	1698	1681	1690	1.0	0.8	0.9	0.8
YPEL5	2924	3098	2986	2300	2404	2383	2523	2692	2346	2185	2147	1.0	0.8	0.8	0.7
FER1L4	1005	996	995	779	810	830	877	950	834	790	786	1.0	0.8	0.9	0.8
MBOAT2	3584	3471	3498	2828	2884	2909	3050	3263	2923	2656	2716	1.0	0.8	0.9	0.8
ELL	738	719	706	629	531	602	619	567	570	569	566	1.0	0.8	0.8	0.8
ARMC7	953	894	903	793	735	788	787	738	773	744	749	1.0	0.8	0.8	0.8
TPST1	850	672	718	602	547	525	623	543	528	563	488	1.0	0.7	0.8	0.7
ARL8A	1887	1976	1938	1589	1469	1496	1697	1592	1424	1453	1395	1.0	0.8	0.8	0.7
ANKRD20A5P	142	124	134	97	92	100	104	98	100	114	88	1.0	0.7	0.8	0.8
LRRC1	1632	1482	1622	1285	1247	1393	1400	1256	1327	1309	1173	1.0	0.8	0.8	0.8
ICAM1	1038	1043	1205	595	662	693	595	500	603	562	474	1.0	0.6	0.5	0.5
KCTD11	1609	1522	1585	1072	1134	1212	1007	1030	1025	1038	1005	1.0	0.7	0.6	0.6
C2CD4C	187	213	218	111	140	126	122	114	116	128	99	1.0	0.6	0.6	0.6
CCDC71L	567	612	553	398	452	444	469	420	417	402	411	1.0	0.7	0.8	0.7
MOB3C	347	370	336	218	233	228	257	192	219	233	196	1.0	0.6	0.6	0.6
PPP2CB	3340	3280	3151	2582	2667	2550	2508	2566	2598	2630	2508	1.0	0.8	0.8	0.8
HOMER3	1443	1499	1400	1263	1177	1176	1206	1191	1223	1254	1206	1.0	0.8	0.8	0.8
ADAM9	7250	7271	7350	5721	5781	5836	6304	6207	6133	5979	6070	1.0	0.8	0.9	0.8
SEMA4B	9999	10082	10201	6471	6828	7206	7461	7680	7580	6878	6868	1.0	0.7	0.8	0.7
COQ10B	1027	979	992	824	844	839	884	863	856	848	813	1.0	0.8	0.9	0.8
TNKS1BP1	7129	7034	7563	6192	6172	6171	6252	6221	6252	6339	6268	1.0	0.9	0.9	0.9
VASN	1157	1160	1236	857	911	906	945	900	919	920	918	1.0	0.8	0.8	0.8
SMURF1	4532	4639	4681	3444	3583	3576	3673	3599	3644	3527	3575	1.0	0.8	0.8	0.8
ARAP2	1736	1782	1934	1338	1323	1341	1509	1345	1455	1331	1321	1.0	0.7	0.8	0.7
SLC12A7	2062	2098	2180	1598	1587	1528	1661	1576	1594	1558	1488	1.0	0.7	0.8	0.7
PLEKHH3	1600	1703	1550	1396	1360	1336	1298	1372	1423	1288	1269	1.0	0.8	0.8	0.8
PVRL4	2011	2084	1939	1605	1307	1322	1371	1448	1513	1106	1119	1.0	0.7	0.7	0.6
NFKBIA	3287	3542	3515	2897	2741	3010	3099	2945	3193	2773	2921	1.0	0.8	0.9	0.8
SDCBP2	321	336	377	257	234	242	252	236	308	248	249	1.0	0.7	0.8	0.7
KIAA0355	505	614	594	482	438	474	447	424	490	446	398	1.0	0.8	0.8	0.7
PCGF3	4742	4456	4641	3904	3734	3885	3970	3828	3954	3616	3641	1.0	0.8	0.8	0.8
PRKCD	2222	2073	2222	1738	1765	1740	1791	1624	1775	1617	1540	1.0	0.8	0.8	0.7
DRAM1	2594	2497	2644	1870	1911	1940	1943	1785	1867	1664	1550	1.0	0.7	0.7	0.6
C7orf43	1451	1347	1478	1152	1139	1174	1209	1109	1094	1030	1017	1.0	0.8	0.8	0.7
TMEM189	4970	5014	5077	4188	4016	3975	4172	3880	3845	3699	3774	1.0	0.8	0.8	0.7
TNFAIP8	1409	1427	1522	1056	1140	1112	1152	1025	1031	985	1004	1.0	0.8	0.7	0.7
ETS2	3203	3285	3340	2419	2513	2446	2440	2421	2368	2255	2315	1.0	0.8	0.7	0.7
MICAL1	1806	1590	1790	1327	1343	1255	1267	1282	1210	1187	1083	1.0	0.8	0.7	0.7
TRAK1	2099	2016	2109	1694	1655	1683	1608	1667	1572	1472	1533	1.0	0.8	0.8	0.7
EHD2	2495	2316	2381	1696	1703	1769	1701	1689	1614	1555	1512	1.0	0.7	0.7	0.6
C22orf29	5538	5188	5603	3681	3707	3761	3480	3435	3545	3083	3108	1.0	0.7	0.6	0.6
SH3RF1	2566	2643	2594	1796	1893	1778	1775	1620	1748	1576	1477	1.0	0.7	0.7	0.6
EPHA2	3332	3494	3591	2078	2243	2300	2015	1940	2091	1611	1792	1.0	0.6	0.6	0.5
GALNT1	8992	8841	8702	6885	7109	7182	6914	6900	6938	6109	6271	1.0	0.8	0.8	0.7
MYO9B	3321	3148	3342	2781	2772	2574	2585	2545	2597	2304	2473	1.0	0.8	0.8	0.7
ABCC3	14321	13238	14676	11368	11404	11050	10340	10285	10273	9461	9408	1.0	0.8	0.7	0.7
FOXP4	4445	4751	5144	3695	3618	3788	3317	3393	3672	3138	3029	1.0	0.8	0.7	0.6
ZSWIM4	977	993	1105	675	593	706	590	577	605	469	488	1.0	0.6	0.6	0.5
CLIP2	1532	1577	1681	1133	1102	1159	1079	1014	1008	988	878	1.0	0.7	0.6	0.6
MKNK2	2412	2404	2467	2126	1959	2058	1948	1920	1916	1875	1787	1.0	0.8	0.8	0.8
HIVEP1	1277	1192	1409	1019	987	1033	996	1029	1033	967	929	1.0	0.8	0.8	0.7
ZSWIM8	3249	3155	3353	2626	2602	2740	2407	2746	2563	2421	2336	1.0	0.8	0.8	0.7

FOXA1	13484	14320	14717	11951	11808	11864	11604	12141	11604	11071	10872	1.0	0.8	0.8	0.8
CGN	7550	7724	7744	6134	5921	5864	6183	5960	6191	5107	4999	1.0	0.8	0.8	0.7
PINK1	1947	1983	2058	1617	1524	1519	1583	1584	1666	1323	1252	1.0	0.8	0.8	0.6
TP53INP2	1183	1170	1274	746	655	689	842	760	794	572	494	1.0	0.6	0.7	0.4
PPL	6800	7225	7264	4682	4680	4548	5198	5248	5317	3913	3760	1.0	0.7	0.7	0.5
ARID3A	1727	1724	1978	1339	1444	1274	1399	1353	1346	1162	1148	1.0	0.7	0.8	0.6
CAB39	5290	5370	5279	4144	4322	4150	4516	4365	4384	3804	3917	1.0	0.8	0.8	0.7
HID1	6387	6422	6327	5109	4955	5170	5295	5347	5195	4736	4573	1.0	0.8	0.8	0.7
PCDH1	4313	4464	4444	2788	2787	2865	2991	3123	2955	2389	2384	1.0	0.6	0.7	0.5
PRSS8	2733	2754	2649	2025	1961	1973	2100	2114	1945	1770	1807	1.0	0.7	0.8	0.7
MCL1	27541	29135	28158	21101	21788	22426	21048	21373	21474	19408	19608	1.0	0.8	0.8	0.7
SPAG9	7119	7217	7200	5471	5809	5714	5677	5647	5804	5265	5172	1.0	0.8	0.8	0.7
IER5	6802	7293	7331	4822	5010	5244	4865	5294	5156	4591	4564	1.0	0.7	0.7	0.6
TEP1	1989	2005	2053	1528	1616	1645	1539	1626	1568	1456	1467	1.0	0.8	0.8	0.7
EPHB3	1638	1612	1685	1152	1109	1174	1089	1253	1165	950	963	1.0	0.7	0.7	0.6
SPINT1	10372	10233	10605	8140	8154	8189	7902	8313	8036	7148	7056	1.0	0.8	0.8	0.7
BCAR3	1877	1813	1794	1278	1236	1312	1261	1329	1303	1107	1149	1.0	0.7	0.7	0.6
MYH14	20399	21073	21164	16242	15891	15931	15743	16315	17108	14457	13940	1.0	0.8	0.8	0.7
PLXNB2	18673	19006	19496	15634	15073	15326	14847	15464	15392	13983	13126	1.0	0.8	0.8	0.7
THBD	3576	3714	3999	2870	2789	2957	2832	3030	2897	2265	2411	1.0	0.8	0.8	0.6
TRAF4	3837	4051	4110	3100	3117	3208	3073	3168	3010	2519	2554	1.0	0.8	0.8	0.6
ZDHHC7	9808	9995	9992	7258	7268	7639	7628	7358	7328	6081	6088	1.0	0.7	0.7	0.6
SAMD4A	2839	2757	2959	2335	2386	2581	2396	2371	2440	2133	2220	1.0	0.9	0.8	0.8
SRC	3008	2940	3163	2397	2536	2578	2456	2472	2457	2243	2152	1.0	0.8	0.8	0.7
TRIM16	2603	2613	2734	2053	2209	2263	2383	2201	2177	1914	1974	1.0	0.8	0.9	0.7
CPEB4	1537	1558	1708	1178	1196	1304	1300	1305	1294	1059	1137	1.0	0.8	0.8	0.7
TSC22D1	5329	5245	5444	4283	4350	4646	4681	4694	4713	4156	4085	1.0	0.8	0.9	0.8
SORBS3	930	971	1004	715	744	824	763	786	796	692	675	1.0	0.8	0.8	0.7
PACSIN1	632	633	637	425	492	461	474	464	428	367	290	1.0	0.7	0.7	0.5
GPR157	395	439	388	294	321	324	331	341	314	300	258	1.0	0.8	0.8	0.7
EFNA2	88	106	98	52	58	83	53	57	50	44	29	1.0	0.7	0.5	0.4
TRIP10	1850	1969	1845	1570	1526	1514	1470	1453	1478	1399	1241	1.0	0.8	0.8	0.7
PHLDA3	5405	6002	5734	4529	4595	4698	4358	4679	4591	4272	3834	1.0	0.8	0.8	0.7
GDPGP1	1256	1364	1178	876	931	969	860	889	863	920	745	1.0	0.7	0.7	0.7
**EFNA1**	**2612**	**2727**	**2755**	**2335**	**2221**	**2326**	**1993**	**2048**	**2022**	**1577**	**1532**	**1.0**	**0.9**	**0.7**	**0.6**
**MXD4**	**7934**	**8088**	**8420**	**7050**	**6757**	**6817**	**6423**	**6554**	**6614**	**5306**	**5345**	**1.0**	**0.8**	**0.8**	**0.7**
USP43	837	833	901	719	629	702	611	622	584	454	494	1.0	0.8	0.7	0.6
AKAP8L	2276	2250	2485	1973	1930	2016	1826	1923	1761	1638	1491	1.0	0.8	0.8	0.7
GET4	274	281	301	262	211	212	203	227	210	156	133	1.0	0.8	0.7	0.5
JDP2	1178	1197	1135	976	954	1011	814	859	892	667	719	1.0	0.8	0.7	0.6
SH2D4A	1532	1531	1413	1222	1111	1190	1054	1088	1095	721	831	1.0	0.8	0.7	0.5
FAM160B1	1391	1419	1395	1190	1194	1201	1123	1108	1226	950	1033	1.0	0.9	0.8	0.7
FILIP1L	4220	4030	4179	3265	3226	3302	3366	3148	3183	2411	2528	1.0	0.8	0.8	0.6
DKK1	14655	14986	14429	11955	11917	11965	12114	11808	12246	8783	9182	1.0	0.8	0.8	0.6
C2orf54	276	246	261	169	154	137	108	90	104	31	34	1.0	0.6	0.4	0.1
**TGFB2**	**10420**	**10117**	**10057**	**7335**	**7720**	**7327**	**6364**	**5872**	**6297**	**4244**	**4229**	**1.0**	**0.7**	**0.6**	**0.4**
CERK	964	883	951	778	729	771	727	685	741	548	525	1.0	0.8	0.8	0.6
MFI2	333	328	387	290	281	249	225	269	288	214	192	1.0	0.8	0.7	0.6
ARL4C	457	436	429	289	309	318	256	262	327	237	209	1.0	0.7	0.6	0.5
IRX5	791	855	830	681	692	676	514	555	603	492	429	1.0	0.8	0.7	0.6
TMEM156	88	93	104	60	63	60	39	37	41	31	23	1.0	0.6	0.4	0.3
CSRNP1	875	873	984	599	563	615	440	462	465	397	305	1.0	0.7	0.5	0.4
SPECC1	4745	4813	4730	3882	3986	4211	3557	3450	3571	3262	3096	1.0	0.8	0.7	0.7
**ARNT2**	**4145**	**3882**	**4092**	**2979**	**3063**	**3335**	**2244**	**2169**	**2493**	**1981**	**1887**	**1.0**	**0.8**	**0.6**	**0.5**
ETNK2	962	913	943	649	641	727	500	463	530	366	379	1.0	0.7	0.5	0.4
LINC00152	676	679	546	422	419	470	266	215	294	214	216	1.0	0.7	0.4	0.3
RCAN1	953	918	858	692	677	753	646	498	569	535	511	1.0	0.8	0.6	0.6
PLEKHG1	325	244	270	217	228	203	171	140	154	134	119	1.0	0.8	0.6	0.5
GOLGA7B	372	310	285	192	224	239	162	152	148	140	104	1.0	0.7	0.5	0.4
EFHD1	191	221	191	139	134	134	127	71	100	75	37	1.0	0.7	0.5	0.3
PLEKHM1P	670	688	699	558	553	634	573	498	451	403	374	1.0	0.8	0.7	0.6
ZC3H12A	838	862	869	629	679	685	674	579	584	482	521	1.0	0.8	0.7	0.6
VPS54	1870	1991	1995	1529	1619	1662	1532	1390	1386	1076	1257	1.0	0.8	0.7	0.6
TBC1D10A	760	837	822	639	600	658	649	644	609	631	633	1.0	0.8	0.8	0.8
ZNF143	553	618	613	522	449	494	503	449	470	479	486	1.0	0.8	0.8	0.8
SUV420H2	422	512	469	420	378	364	432	366	340	343	389	1.0	0.8	0.8	0.8

ERICH1	413	387	376	291	336	322	301	315	294	351	326	1.0	0.8	0.8	0.9
LIF	1809	1700	1905	1073	1162	1227	1049	1046	1058	1300	1208	1.0	0.6	0.6	0.7
BHLHE40	3960	3937	4170	2558	2628	2752	2501	2732	2425	2633	2630	1.0	0.7	0.6	0.7
KLF13	1832	1835	1866	1394	1389	1512	1391	1396	1408	1470	1398	1.0	0.8	0.8	0.8
GPER1	177	211	185	125	122	164	122	130	104	145	128	1.0	0.7	0.6	0.7
ERO1L	4665	4542	4567	3462	3553	3458	3372	3702	3444	3480	3931	1.0	0.8	0.8	0.8
TMEM45A	447	462	452	288	331	317	284	283	265	246	302	1.0	0.7	0.6	0.6
EGR2	552	624	638	215	304	309	205	285	208	236	230	1.0	0.5	0.4	0.4
ANGPTL4	1634	1645	1750	879	989	1026	824	1022	811	864	900	1.0	0.6	0.5	0.5
HSBP1L1	535	447	451	370	361	354	351	368	284	302	339	1.0	0.8	0.7	0.7
CPM	772	708	750	624	653	614	592	576	513	531	646	1.0	0.8	0.8	0.8
SERINC5	922	811	899	714	696	704	560	634	585	594	568	1.0	0.8	0.7	0.7
MAPK11	698	577	706	497	501	468	310	397	342	327	289	1.0	0.7	0.5	0.5
ARHGEF18	885	859	924	738	734	721	560	696	575	593	630	1.0	0.8	0.7	0.7
PLCH2	131	134	145	91	99	62	54	78	43	50	34	1.0	0.6	0.4	0.3
LIMD2	305	329	297	233	255	211	189	239	177	191	177	1.0	0.8	0.7	0.6
GPRC5C	1869	1842	1803	1583	1624	1418	1295	1485	1385	1119	1312	1.0	0.8	0.8	0.7
TAGLN	2702	2703	2594	2095	2102	2145	596	563	515	469	487	1.0	0.8	0.2	0.2
TGFBI	4274	4108	4271	3044	3124	3021	635	541	537	428	434	1.0	0.7	0.1	0.1
AMPH	900	912	948	659	653	634	18	11	10	7	5	1.0	0.7	0.0	0.0
ANKRD34B	962	912	891	728	751	778	92	92	85	61	81	1.0	0.8	0.1	0.1
IGFL1	545	445	489	349	327	263	18	10	21	10	15	1.0	0.6	0.0	0.0
PRKCA	3523	3231	3493	2897	3008	2856	1150	1185	1272	1079	1239	1.0	0.9	0.4	0.3
FOXI1	225	191	205	149	128	144	22	19	20	8	15	1.0	0.7	0.1	0.1
UBE2QL1	1931	1875	1882	1085	1200	1168	128	115	92	51	90	1.0	0.6	0.1	0.0
CSRP1	25575	25297	25029	20461	20280	20538	7685	7466	7696	6232	6378	1.0	0.8	0.3	0.2
SOCS2	1951	1927	1852	1631	1652	1587	590	548	596	464	514	1.0	0.8	0.3	0.3
BASP1	7689	7627	7812	6673	6413	6220	3339	3407	3189	2835	2873	1.0	0.8	0.4	0.4
NPAS2	1735	1664	1754	1496	1487	1369	824	722	756	642	675	1.0	0.8	0.4	0.4
DIO2-AS1	1288	1303	1490	1223	1127	1023	376	395	427	248	273	1.0	0.8	0.3	0.2
FBN2	1567	1687	1648	1397	1372	1359	350	316	349	206	217	1.0	0.8	0.2	0.1
DIO2	83175	77614	80850	64414	65642	62630	22644	21661	22329	15681	16428	1.0	0.8	0.3	0.2
NR2F1	420	355	413	289	251	332	43	58	54	33	50	1.0	0.7	0.1	0.1
GPR98	1894	2074	2012	1536	1630	1601	489	560	556	477	483	1.0	0.8	0.3	0.2
FAM84A	904	923	951	743	778	717	191	254	254	178	203	1.0	0.8	0.3	0.2
ACTG2	482	488	525	348	388	470	16	29	34	17	8	1.0	0.8	0.1	0.0
WTIP	650	705	666	512	558	591	220	193	195	178	165	1.0	0.8	0.3	0.3
MMP9	3763	3556	3723	1894	2170	2205	116	91	112	93	62	1.0	0.6	0.0	0.0
SLCO2A1	4806	4664	5020	3187	3306	3272	558	489	594	433	379	1.0	0.7	0.1	0.1
C4orf26	1556	1587	1371	1075	1134	1155	203	197	220	156	136	1.0	0.7	0.1	0.1
PRKAR2B	1960	1960	1902	1612	1515	1607	614	586	627	613	489	1.0	0.8	0.3	0.3
HMOX1	378	332	366	278	290	296	138	154	200	127	135	1.0	0.8	0.5	0.4
C3orf36	181	191	176	105	109	78	6	1	17	2	0	1.0	0.5	0.0	0.0
VIPR2	229	245	260	200	205	180	77	70	99	60	75	1.0	0.8	0.3	0.3
CCNYL1	1508	1332	1426	1125	1281	1171	840	911	909	853	810	1.0	0.8	0.6	0.6
PDZD2	158	132	176	91	109	108	33	58	47	38	33	1.0	0.7	0.3	0.2
SPIRE1	5275	5034	5494	4282	4411	4459	3225	3152	3267	3060	3108	1.0	0.8	0.6	0.6
PLEKHH2	1308	1120	1266	725	835	801	348	335	392	279	286	1.0	0.6	0.3	0.2
PTK2B	438	316	409	247	283	268	93	105	97	83	89	1.0	0.7	0.3	0.2
ADM2	189	157	220	129	140	147	49	44	57	34	42	1.0	0.7	0.3	0.2
SLC4A3	591	482	573	417	421	484	250	274	283	240	280	1.0	0.8	0.5	0.5
AHNAK2	1267	1297	1358	1078	1160	1105	900	821	985	773	772	1.0	0.9	0.7	0.6
TRAM2	2209	2150	2423	1923	1927	1862	1459	1428	1578	1271	1200	1.0	0.8	0.7	0.5
PLEKHG4B	167	174	193	139	130	107	70	55	68	37	38	1.0	0.7	0.4	0.2
SOWAHB	1442	1448	1482	1150	1031	1087	627	644	682	486	480	1.0	0.7	0.4	0.3
TACC1	5763	5678	5628	4890	4727	4613	3036	2858	3364	2368	2384	1.0	0.8	0.5	0.4
MUC3A	1004	988	1038	693	668	731	279	294	306	184	129	1.0	0.7	0.3	0.2
ACHE	4667	4309	4700	2829	2816	2872	923	848	1006	540	434	1.0	0.6	0.2	0.1
PRICKLE1	1068	996	965	775	807	797	529	398	527	344	348	1.0	0.8	0.5	0.3
STON1	444	479	422	372	377	373	201	169	202	119	123	1.0	0.8	0.4	0.3
SALL4	161	202	172	118	125	121	53	48	61	42	21	1.0	0.7	0.3	0.2
MROH2A	244	268	268	181	180	174	118	83	107	85	63	1.0	0.7	0.4	0.3
SCARA3	1152	1268	1321	1032	933	956	758	696	819	560	560	1.0	0.8	0.6	0.4
SYNGR3	712	811	776	632	568	590	494	434	447	364	358	1.0	0.8	0.6	0.5
GRHL3	1974	2014	2028	1217	1203	1176	657	614	717	379	323	1.0	0.6	0.3	0.2
LMCD1	1768	1986	1910	1236	1217	1188	737	697	780	514	475	1.0	0.6	0.4	0.3

N4BP3	4791	5003	5188	3509	3265	3595	2430	2514	2500	1717	1647	1.0	0.7	0.5	0.3
IDH2	15173	15598	15649	13122	13040	13448	11402	11413	11618	10143	9955	1.0	0.9	0.7	0.6
SNAI2	802	777	743	597	683	623	471	525	511	385	395	1.0	0.8	0.6	0.5
CHKA	1862	1801	1857	1517	1526	1492	1266	1247	1239	1053	1134	1.0	0.8	0.7	0.6
LIMA1	2193	2107	2090	1511	1488	1506	1088	1007	895	706	806	1.0	0.7	0.5	0.4
**ANXA3**	**5462**	**5380**	**4844**	**3987**	**3831**	**4024**	**3078**	**2929**	**3072**	**2332**	**2411**	**1.0**	**0.8**	**0.6**	**0.5**
ATG16L1	3126	2951	2873	2496	2526	2580	2189	2221	2180	1843	1909	1.0	0.8	0.7	0.6
**MYO1B**	**24177**	**24202**	**24060**	**19768**	**20586**	**20152**	**15951**	**15185**	**15720**	**11978**	**12811**	**1.0**	**0.8**	**0.6**	**0.5**
IFFO2	703	676	649	515	565	515	338	337	357	241	293	1.0	0.8	0.5	0.4
NBEA	1941	1783	1935	1413	1523	1419	958	966	1038	773	770	1.0	0.8	0.5	0.4
GALNT10	20865	20372	20650	16590	16332	16341	11624	11615	11647	9315	9624	1.0	0.8	0.6	0.5
ARHGAP5	6075	6177	6133	5172	5298	5125	4093	4065	4142	3545	3662	1.0	0.8	0.7	0.6
ZFAND3	4623	4678	4523	3969	3876	3955	3241	3269	3347	3067	2963	1.0	0.9	0.7	0.7
JUP	30062	30006	30307	25243	24180	24275	20346	20284	19979	18280	17960	1.0	0.8	0.7	0.6
TRAFD1	4080	3988	3956	3231	3070	2965	2282	2338	2288	1963	1908	1.0	0.8	0.6	0.5
ID3	3888	3906	3835	3419	3117	3035	2347	2412	2432	2127	2107	1.0	0.8	0.6	0.5
DNAJC22	4209	4312	4410	2807	2808	2875	1605	1592	1518	1244	1128	1.0	0.7	0.4	0.3
MTSS1L	12206	12338	12582	10256	9741	9935	7816	7811	7448	7110	6923	1.0	0.8	0.6	0.6
RHOBTB2	2328	2231	2457	1846	1651	1885	1213	1163	1260	1046	1082	1.0	0.8	0.5	0.5
MIR4435-1HG	1461	1499	1433	1111	1050	1212	645	695	715	614	587	1.0	0.8	0.5	0.4
HBEGF	1670	1779	1656	789	829	986	273	306	347	240	236	1.0	0.5	0.2	0.1
KRT80	123697	117708	123820	92040	94237	93537	64967	63343	64155	53816	54199	1.0	0.8	0.5	0.4
PXN	30681	29495	30744	23218	23927	23916	16331	15963	16209	14336	14351	1.0	0.8	0.5	0.5
RAPGEF2	3205	3146	3250	2502	2669	2555	1875	1845	1795	1604	1641	1.0	0.8	0.6	0.5
CRIM1	8485	8718	8547	6189	6471	6358	3805	3857	3911	3322	3410	1.0	0.7	0.4	0.4
CTNNA1	27691	27572	26914	23005	23457	23283	17494	17697	17215	16263	15899	1.0	0.8	0.6	0.6
EPAS1	36634	35446	37014	26947	28280	28119	15267	15569	15387	12675	13183	1.0	0.8	0.4	0.4
ZYX	21657	21767	22474	16903	17057	17827	10462	10646	10510	9353	9020	1.0	0.8	0.5	0.4
TP53I3	1425	1390	1315	1015	1014	1107	704	689	651	570	581	1.0	0.8	0.5	0.4
FAM107B	1967	1917	1916	1520	1518	1493	1095	943	994	810	853	1.0	0.8	0.5	0.4
DGKD	5986	5608	5865	4662	4479	4549	3166	2965	3020	2497	2655	1.0	0.8	0.5	0.4
HIP1R	6410	6400	6715	5188	5068	5409	3761	3455	3659	3254	3007	1.0	0.8	0.6	0.5
NEDD9	5566	5790	5620	4130	4092	4434	2862	2575	2781	2264	2171	1.0	0.7	0.5	0.4
UACA	2985	3172	2948	2350	2460	2418	1741	1698	1770	1517	1616	1.0	0.8	0.6	0.5
KLHL5	6841	6956	7003	5274	5426	5186	3951	3590	4052	3288	3330	1.0	0.8	0.6	0.5
PDLIM5	7076	7167	7153	5558	5737	5906	4425	4065	4386	3550	3826	1.0	0.8	0.6	0.5
CACNG6	555	604	571	441	348	347	149	100	96	78	86	1.0	0.7	0.2	0.1
SLC25A30	1165	1215	1207	1076	1049	921	646	613	616	505	558	1.0	0.8	0.5	0.4
CPA4	131	135	143	98	104	85	34	44	34	25	25	1.0	0.7	0.3	0.2
WNT11	82	117	131	61	59	61	5	7	5	3	1	1.0	0.5	0.1	0.0
ITGA3	13383	13226	14011	10435	10504	10892	5629	6066	5873	5315	5436	1.0	0.8	0.4	0.4
RASD1	3921	4059	4089	1761	1692	1885	146	174	181	87	110	1.0	0.4	0.0	0.0
FSCN1	4819	4846	5017	3945	3832	3793	1974	2058	2001	1898	1709	1.0	0.8	0.4	0.4
ASAP2	9258	9147	9083	7356	7564	7189	4350	4402	4186	3978	4020	1.0	0.8	0.5	0.4
RAB32	3780	3701	3704	2919	2864	2934	1489	1560	1428	1321	1358	1.0	0.8	0.4	0.4
PTGER4	2204	2274	2181	1638	1563	1666	556	571	529	402	359	1.0	0.7	0.2	0.2
CARD14	950	918	929	746	711	801	394	423	415	330	324	1.0	0.8	0.4	0.4
TMEM184A	5702	5948	6141	4937	4612	4748	3023	3118	3011	2631	2720	1.0	0.8	0.5	0.5
SYNPO	4767	4547	4869	3027	3014	2852	842	917	973	621	596	1.0	0.6	0.2	0.1
PACS1	16139	15623	16907	14300	13859	13429	9375	8926	9232	7997	7928	1.0	0.9	0.6	0.5
HDAC7	5858	5603	5586	4854	4757	4812	3167	3058	3090	2668	2736	1.0	0.8	0.5	0.5
RND1	2307	2426	2416	1467	1423	1525	488	450	423	304	369	1.0	0.6	0.2	0.1
PAWR	15922	16734	16144	12822	13442	13374	8123	7754	8183	6631	6935	1.0	0.8	0.5	0.4
MKL1	3786	3623	3749	3267	3094	3093	2996	3033	2970	2728	2536	1.0	0.8	0.8	0.7
CACNB3	3542	3610	3555	3037	2912	2906	2751	2695	2636	2495	2284	1.0	0.8	0.8	0.7
TAF10	2792	2560	2643	2337	2188	2295	1985	2119	2041	1862	1812	1.0	0.9	0.8	0.7
THEG	525	401	455	283	267	289	167	188	186	134	115	1.0	0.6	0.4	0.3
C7orf26	1567	1609	1530	1304	1290	1370	1241	1261	1241	1187	1140	1.0	0.8	0.8	0.7
GATA6	696	817	735	489	431	546	407	388	387	302	267	1.0	0.7	0.5	0.4
RHOC	5960	5791	5959	5066	4859	4966	4424	4297	4209	4027	3825	1.0	0.8	0.7	0.7
LMO7	6084	5773	5815	3850	3728	3676	2766	2569	2753	2245	2077	1.0	0.6	0.5	0.4
ABLIM3	2426	2260	2295	2013	1933	1918	1650	1614	1624	1577	1479	1.0	0.8	0.7	0.7
BAIAP2	2099	2005	2116	1704	1626	1604	1234	1230	1237	1180	1077	1.0	0.8	0.6	0.5
SLC5A10	506	518	526	405	436	418	342	339	342	321	304	1.0	0.8	0.7	0.6
USP2	481	484	528	289	295	306	153	152	177	133	120	1.0	0.6	0.3	0.3
SH2D3A	1815	1959	1920	1329	1340	1382	915	913	979	802	738	1.0	0.7	0.5	0.4

SLC12A2	5106	4955	5099	4103	4282	4110	3664	3757	3561	3204	3148	1.0	0.8	0.7	0.6
RAB5B	1808	1701	1773	1455	1482	1484	1246	1273	1226	1058	1078	1.0	0.8	0.7	0.6
DRD1	471	439	480	221	238	219	134	111	114	69	65	1.0	0.5	0.3	0.1
CSNK1D	8370	8525	8617	7362	7131	7332	6353	6656	6194	5855	5616	1.0	0.9	0.8	0.7
CAP1	10994	10447	10709	8898	9297	9128	7873	8241	7801	7336	7548	1.0	0.8	0.7	0.7
ATP2B1	12504	11965	12214	10217	10147	10012	8873	8950	8706	8170	8449	1.0	0.8	0.7	0.7
PRKAR1A	21750	21420	21077	18007	18462	18118	16358	16249	16177	15240	15413	1.0	0.8	0.8	0.7
PPP2R5B	1188	1214	1110	934	887	874	711	789	661	598	649	1.0	0.8	0.6	0.5
RAB11FIP4	2561	2574	2423	1977	1851	1936	1558	1587	1371	1237	1248	1.0	0.8	0.6	0.5
ILK	2815	2931	3017	2548	2332	2338	2099	2084	1987	1820	1916	1.0	0.8	0.7	0.6
PDGFA	1416	1357	1437	1139	1197	1099	1042	1128	1129	920	966	1.0	0.8	0.8	0.7
CRISPLD2	542	545	592	376	395	370	312	393	356	250	276	1.0	0.7	0.6	0.5
CDYL2	8781	8332	8721	6515	6710	6444	6204	6410	6235	5137	5440	1.0	0.8	0.7	0.6
MAP7D1	3358	3602	3709	2926	2981	2880	2517	2702	2633	2455	2328	1.0	0.8	0.7	0.7
UBTD1	931	1014	1071	805	849	735	658	731	667	609	603	1.0	0.8	0.7	0.6
C1QL4	176	169	173	113	133	92	79	98	90	71	63	1.0	0.7	0.5	0.4
POFUT2	2321	2246	2213	1891	1892	1786	1654	1785	1711	1633	1590	1.0	0.8	0.8	0.7
EHBP1L1	3337	3247	3304	2374	2413	2419	2086	2117	2104	1787	1728	1.0	0.7	0.6	0.5
KIAA0513	8002	7851	8042	4662	4695	4775	3883	3554	3838	2793	2588	1.0	0.6	0.5	0.3
**ERBB2**	**10146**	**10314**	**10277**	**7876**	**7527**	**7676**	**6678**	**6665**	**6670**	**5913**	**5388**	**1.0**	**0.8**	**0.7**	**0.6**
WNT7B	4592	4557	4638	3474	3219	3283	2841	2894	2904	2399	2296	1.0	0.7	0.6	0.5
NRBP1	10550	10245	10259	8824	8516	8776	8346	7918	8065	7476	7370	1.0	0.8	0.8	0.7
AMOTL2	7570	7930	7997	6809	6547	6514	6174	5970	6054	5208	5359	1.0	0.8	0.8	0.7
GRAMD1A	2997	3023	3007	2412	2359	2323	2283	2109	2100	1808	1796	1.0	0.8	0.7	0.6
MISP	5270	5536	5266	4364	4410	4242	3922	3807	3762	3310	3284	1.0	0.8	0.7	0.6
GPR39	3693	3679	3674	2884	2953	2947	2435	2345	2398	2055	2064	1.0	0.8	0.6	0.6
TUFT1	7336	7260	7267	4790	5005	5130	3639	3448	3687	2631	2831	1.0	0.7	0.5	0.4
SMAD3	20614	19810	20672	13745	14265	14810	10233	9867	10411	8490	8592	1.0	0.7	0.5	0.4
LRP10	20123	19731	20915	16478	16094	16157	13894	14295	13985	12357	12625	1.0	0.8	0.7	0.6
MBNL2	4006	4091	4300	3132	3264	3200	2904	2638	2675	2279	2361	1.0	0.8	0.7	0.6
LYSMD3	1720	1793	1853	1236	1307	1255	1005	919	1058	754	761	1.0	0.7	0.6	0.4
PMEPA1	79402	82529	81906	60283	60301	62170	52284	52685	52895	44169	44165	1.0	0.7	0.6	0.5
THBS1	81468	82227	85346	56289	58377	58956	47258	45772	46944	38161	38351	1.0	0.7	0.6	0.5
MYADM	10206	10293	11180	5315	5534	5945	3890	3792	3854	2751	2840	1.0	0.5	0.4	0.3
CTGF	3058	3530	3441	1464	1462	1617	926	841	935	490	482	1.0	0.5	0.3	0.1
SH3BP4	5927	6210	6186	4220	4115	4329	3580	3310	3493	2655	2726	1.0	0.7	0.6	0.4
OPTN	3769	3796	3758	2890	2990	3019	2662	2478	2629	2241	2265	1.0	0.8	0.7	0.6
RAB6B	1291	1226	1247	990	1078	1019	929	909	943	806	770	1.0	0.8	0.7	0.6
UBL3	5753	5672	5732	4595	4823	4712	4350	4188	4289	3587	3887	1.0	0.8	0.7	0.7
KAT2B	1408	1396	1360	986	1118	1051	975	880	904	710	767	1.0	0.8	0.7	0.5
**ENC1**	**4687**	**4857**	**4838**	**2951**	**3331**	**3463**	**2433**	**2592**	**2449**	**1926**	**1982**	**1.0**	**0.7**	**0.5**	**0.4**
STK38L	5529	5867	5516	3743	4187	4409	3430	3458	3471	2744	2810	1.0	0.7	0.6	0.5
SIM2	1814	1792	1822	981	1071	1126	792	774	821	522	561	1.0	0.6	0.4	0.3
SIK1	5283	5238	5398	4093	4214	4334	3653	3711	3698	3017	3130	1.0	0.8	0.7	0.6
GRAMD3	1602	1739	1679	1179	1366	1354	1135	1221	1094	1004	1004	1.0	0.8	0.7	0.6
HES1	1472	1665	1601	1192	1178	1182	1031	1104	1066	827	910	1.0	0.7	0.7	0.6
EVA1B	463	543	506	362	361	340	290	298	314	231	239	1.0	0.7	0.6	0.5
DAPP1	148	161	143	63	94	67	52	54	62	34	44	1.0	0.5	0.4	0.3
MYL12A	24401	24479	23478	18550	19538	19455	16729	17306	18180	15584	16171	1.0	0.8	0.7	0.7
MFSD6	1595	1511	1515	1276	1176	1267	1171	1084	1161	919	1059	1.0	0.8	0.7	0.6
OCLN	2126	2020	2006	1618	1617	1722	1509	1404	1552	1271	1296	1.0	0.8	0.7	0.6
SLC26A11	1336	1290	1325	1042	985	1099	856	784	822	722	798	1.0	0.8	0.6	0.6
VCL	7296	7032	7551	5869	5933	6186	5492	5106	5320	4848	4954	1.0	0.8	0.7	0.7
KLHL25	1333	1229	1308	1084	1023	1079	963	886	900	813	833	1.0	0.8	0.7	0.6
ARID3B	418	376	482	315	295	339	259	223	242	181	218	1.0	0.7	0.6	0.5
DLL1	333	317	377	248	272	251	198	205	234	128	177	1.0	0.8	0.6	0.4
WNT9A	211	209	276	150	150	192	127	131	136	85	108	1.0	0.7	0.6	0.4
USB1	1516	1280	1431	1194	1208	1181	1054	1065	1090	1048	1073	1.0	0.8	0.8	0.8
CTDSPL	441	409	423	361	356	344	342	302	340	321	308	1.0	0.8	0.8	0.7
DHX15	10677	10261	10451	8918	8676	8764	8609	8002	8396	8256	7955	1.0	0.8	0.8	0.8
KLC3	671	632	607	553	529	498	474	432	510	454	450	1.0	0.8	0.7	0.7
SYDE2	1029	1017	1022	880	908	815	814	734	838	710	711	1.0	0.8	0.8	0.7
TNFAIP1	4372	4268	4285	3480	3563	3391	3360	3009	3263	2875	2863	1.0	0.8	0.7	0.7
INPP4A	2239	2208	2190	1951	1865	1828	1719	1517	1694	1479	1457	1.0	0.9	0.7	0.7
CSF1	265	219	286	151	196	161	107	107	165	111	132	1.0	0.7	0.5	0.5
MN1	148	158	165	125	99	108	46	61	78	49	60	1.0	0.7	0.4	0.3

PPAP2B	412	444	412	367	360	323	230	261	289	271	245	1.0	0.8	0.6	0.6
CMIP	3602	3482	3860	3090	3052	3061	2726	2785	2901	2675	2751	1.0	0.8	0.8	0.7
PNPLA6	1271	1317	1480	1117	1088	1083	912	962	956	959	935	1.0	0.8	0.7	0.7
P2RY6	42	40	69	20	31	25	7	6	15	6	6	1.0	0.5	0.2	0.1
AP5B1	471	448	558	402	395	405	320	301	360	323	296	1.0	0.8	0.7	0.6
FAM228B	262	269	259	231	210	183	147	140	159	135	144	1.0	0.8	0.6	0.5
PPFIA1	5989	6256	6171	5292	5208	5135	4659	4500	4674	4171	4350	1.0	0.8	0.8	0.7
UTRN	1751	1754	1802	1303	1407	1222	992	954	1071	787	938	1.0	0.7	0.6	0.5
MAPK8IP2	269	234	246	182	198	162	104	96	116	98	84	1.0	0.7	0.4	0.4
**KRT7**	**1840**	**1732**	**1603**	**1248**	**1335**	**1213**	**894**	**930**	**1038**	**835**	**839**	**1.0**	**0.7**	**0.6**	**0.5**
F3	475	374	449	249	273	303	163	147	173	154	154	1.0	0.6	0.4	0.4
FHDC1	1465	1331	1443	1207	1165	1186	1015	982	979	973	999	1.0	0.8	0.7	0.7
HES2	215	225	221	140	140	147	68	65	86	76	82	1.0	0.6	0.3	0.4
PYCARD	1866	1876	1866	1586	1494	1557	1072	1176	1202	1141	1155	1.0	0.8	0.6	0.6
MALL	6380	5963	6409	4334	4526	4446	3354	3391	3578	3333	3215	1.0	0.7	0.6	0.5
ZC3H7B	7526	7508	7888	6015	6230	6059	4791	4799	5060	4991	4832	1.0	0.8	0.6	0.6
SHC1	5159	5041	5151	4120	4109	4247	3338	3493	3652	3442	3240	1.0	0.8	0.7	0.7
RAB11FIP1	3653	3502	3515	2829	2920	2818	2312	2454	2486	2316	2472	1.0	0.8	0.7	0.7
STAT3	7127	7119	7283	5886	6100	6052	4909	4829	4786	4689	4611	1.0	0.8	0.7	0.6
IL4R	4693	4618	4816	3545	3611	3650	2676	2596	2673	2529	2505	1.0	0.8	0.6	0.5
SLC4A7	4958	4870	4943	3617	3900	3807	2762	2764	2840	2615	2595	1.0	0.8	0.6	0.5
PTRF	5155	5247	5201	3386	3547	3720	2199	2117	2178	1901	1983	1.0	0.7	0.4	0.4
TSPAN14	5158	5199	5265	4204	4208	4303	3507	3348	3417	3270	3205	1.0	0.8	0.7	0.6
ELMSAN1	5568	5509	5871	4549	4599	4778	3697	3647	3858	3557	3553	1.0	0.8	0.7	0.6
TMEM8A	7443	7388	7611	6057	5848	6120	4457	4657	4690	4480	4293	1.0	0.8	0.6	0.6
FURIN	9184	9009	9647	7163	6812	7082	4993	5249	4820	4713	4766	1.0	0.8	0.5	0.5
BACE2	1280	1188	1329	951	972	979	666	671	675	686	662	1.0	0.8	0.5	0.5
RNF121	3118	2846	2981	2312	2464	2332	1762	1753	1818	1614	1611	1.0	0.8	0.6	0.5
MAP3K9	3585	3368	3556	2865	2948	2803	2223	2262	2304	2052	2082	1.0	0.8	0.6	0.6
LYPD1	4208	4121	4365	3287	3589	3451	2533	2701	2620	2358	2381	1.0	0.8	0.6	0.6
PXN-AS1	8645	8693	8449	6316	6826	6469	4505	4743	4681	4201	4084	1.0	0.8	0.5	0.5
ITGAV	39597	39494	40006	27234	29341	27604	18780	18475	18779	15476	16392	1.0	0.7	0.5	0.4
ITGB1	55979	54535	54674	45934	47797	47011	38561	39152	38061	37737	38505	1.0	0.9	0.7	0.7
VMP1	23733	22666	23141	18851	19674	19780	15696	15237	15352	14183	14906	1.0	0.8	0.7	0.6
DAPK3	4295	4692	4544	3527	3590	3770	2405	2449	2385	2228	2096	1.0	0.8	0.5	0.5
FRMD6	8381	8880	8832	5639	6141	6522	2925	3001	2744	2537	2647	1.0	0.7	0.3	0.3
NFKB2	6810	6931	7308	5106	5332	5380	3439	3090	3283	3065	2991	1.0	0.8	0.5	0.4
**DUSP1**	**1430**	**1475**	**1445**	**932**	**939**	**976**	**467**	**409**	**411**	**381**	**385**	**1.0**	**0.7**	**0.3**	**0.3**
PFDN1	2465	2647	2667	2095	2190	2276	1788	1753	1658	1655	1622	1.0	0.8	0.7	0.6
PLCB1	1401	1550	1482	1202	1214	1224	976	920	943	934	977	1.0	0.8	0.6	0.6
ZNF281	2855	2923	2897	2280	2402	2409	1821	1739	1743	1647	1881	1.0	0.8	0.6	0.6
TMPRSS2	233	232	228	137	134	156	79	87	69	65	84	1.0	0.6	0.3	0.3
F2RL1	1068	1037	1071	590	598	621	362	392	320	283	332	1.0	0.6	0.3	0.3
EFHD2	2983	2899	3083	2406	2502	2529	2037	2084	1926	1958	2060	1.0	0.8	0.7	0.7
B3GNT5	177	185	207	110	114	147	78	65	84	62	83	1.0	0.7	0.4	0.4
NFKBIE	758	817	907	546	623	697	484	431	430	387	434	1.0	0.8	0.5	0.5
S100A3	58	38	61	19	30	36	13	8	5	8	9	1.0	0.5	0.2	0.2
RUFY3	569	571	557	463	411	493	356	407	366	349	405	1.0	0.8	0.7	0.7
CDC42EP1	637	750	735	509	576	630	469	407	416	490	401	1.0	0.8	0.6	0.6
SLC9A5	364	363	312	252	246	312	225	221	215	235	198	1.0	0.8	0.6	0.6
KDM6B	3245	3224	3756	2334	2436	2572	1871	1833	1831	1666	1623	1.0	0.7	0.5	0.5
SESN2	1049	1072	1165	808	823	881	724	709	641	633	626	1.0	0.8	0.6	0.6
PDLIM2	465	483	517	395	397	413	366	373	361	356	343	1.0	0.8	0.8	0.7
NCOA7	975	989	1040	693	740	840	644	630	646	637	560	1.0	0.8	0.6	0.6
PLEKHG2	2784	2745	2841	2052	2130	2191	1886	1778	1703	1795	1679	1.0	0.8	0.6	0.6
MAP2K3	3545	3586	3752	2917	3084	3013	2725	2707	2826	2574	2501	1.0	0.8	0.8	0.7
BCAR1	9246	9469	9789	6993	6973	7126	5934	6387	6212	5618	5478	1.0	0.7	0.7	0.6
KRT86	6029	5875	6129	3895	4032	4089	2903	3267	3467	2765	2674	1.0	0.7	0.5	0.5
SH3GL1	4976	5043	4974	4077	3966	4188	3518	3517	3656	3257	3238	1.0	0.8	0.7	0.6
LITAF	5953	5886	5945	4440	4422	4527	3736	3747	3795	3388	3375	1.0	0.8	0.6	0.6
RHOV	1125	1257	1251	767	779	875	582	597	633	551	542	1.0	0.7	0.5	0.5
IFNGR1	1728	1768	1711	1320	1378	1343	1126	1093	1104	1036	1070	1.0	0.8	0.6	0.6
SDC4	7703	7803	7933	5996	6174	6355	5089	5051	5074	4878	4977	1.0	0.8	0.6	0.6
SEMA7A	2290	2395	2529	1231	1350	1342	931	847	872	744	723	1.0	0.5	0.4	0.3
**BAK1**	**3089**	**3162**	**3172**	**2188**	**2233**	**2297**	**1933**	**1816**	**1763**	**1578**	**1542**	**1.0**	**0.7**	**0.6**	**0.5**
GLIS2	2196	2233	2213	1633	1633	1664	1436	1347	1379	1208	1185	1.0	0.7	0.6	0.5

JUN	4524	5145	5186	4134	3832	4194	3289	3556	3319	3111	3234	1.0	0.8	0.7	0.6
MAFK	1787	2129	2109	1660	1479	1582	1121	1272	1184	1163	1133	1.0	0.8	0.6	0.6
MAFG	1373	1559	1472	1214	1113	1186	893	1024	1024	903	874	1.0	0.8	0.7	0.6
PRKCZ	1651	1711	1680	1453	1390	1421	1200	1329	1309	1195	1146	1.0	0.8	0.8	0.7
NEDD4L	1506	1556	1460	1185	1099	1145	893	937	1032	904	891	1.0	0.8	0.6	0.6
SERTAD1	1365	1555	1465	938	1077	1034	851	932	873	899	862	1.0	0.7	0.6	0.6
TNFRSF12A	9252	10127	9536	7062	7393	7376	6020	6641	6235	6184	5650	1.0	0.8	0.7	0.6
RAB22A	5304	5588	5506	4405	4666	4649	4293	4291	4351	4058	4177	1.0	0.8	0.8	0.8
SOWAHC	1204	1318	1276	767	820	817	643	626	664	588	618	1.0	0.6	0.5	0.5
VEGFC	279	286	289	222	234	213	196	195	175	185	195	1.0	0.8	0.7	0.7
FAM83G	2953	3253	3152	2410	2685	2484	2100	2161	1913	1999	1974	1.0	0.8	0.7	0.6
TUBB3	729	792	806	588	663	651	524	608	509	583	545	1.0	0.8	0.7	0.7
MSX1	348	371	392	248	290	274	156	212	220	226	179	1.0	0.7	0.5	0.5
EGFR-AS1	91	89	106	59	77	60	37	39	41	39	33	1.0	0.7	0.4	0.4
NES	320	312	347	170	212	204	112	105	94	135	92	1.0	0.6	0.3	0.3
MAPRE3	1279	1236	1350	968	1027	1019	774	850	732	822	749	1.0	0.8	0.6	0.6
NFE2L3	1576	1546	1638	1256	1349	1309	1089	1005	1162	1245	1231	1.0	0.8	0.7	0.8
CHST3	306	287	307	236	256	244	188	138	171	204	171	1.0	0.8	0.6	0.6
AFAP1-AS1	326	323	333	227	261	256	226	186	170	223	231	1.0	0.8	0.6	0.7
STRA6	48	40	57	25	33	13	4	6	3	1	6	1.0	0.5	0.1	0.1
ZSWIM3	238	221	250	201	194	151	121	115	91	89	99	1.0	0.8	0.5	0.4
B4GALNT3	61	86	77	39	58	38	22	11	17	12	26	1.0	0.6	0.2	0.3
CCRN4L	531	487	563	479	442	407	340	270	316	279	339	1.0	0.8	0.6	0.6
PRKD1	577	546	567	490	481	434	285	292	261	287	237	1.0	0.8	0.5	0.5
HMGA2	176	155	163	98	119	92	28	24	17	26	15	1.0	0.6	0.1	0.1
SCUBE3	864	773	862	408	460	410	48	52	42	64	32	1.0	0.5	0.1	0.1
COL6A2	589	611	642	481	543	468	150	138	129	125	88	1.0	0.8	0.2	0.2
CAMK1D	2857	2954	2899	2359	2581	2421	1349	1271	1279	1067	977	1.0	0.8	0.4	0.4
TFPI	939	1046	869	628	727	613	231	259	229	219	165	1.0	0.7	0.3	0.2
IFI6	887	1147	1059	682	824	765	217	162	139	192	114	1.0	0.7	0.2	0.1
IL32	476	575	573	341	367	350	144	101	131	107	91	1.0	0.7	0.2	0.2
FMNL2	1117	1277	1272	961	1129	992	651	555	582	613	582	1.0	0.8	0.5	0.5
FSCN2	228	275	267	217	203	172	84	93	51	77	73	1.0	0.8	0.3	0.3
SEMA5B	453	443	480	352	331	317	63	68	35	44	46	1.0	0.7	0.1	0.1
CACNG8	612	586	580	388	457	393	86	86	54	62	102	1.0	0.7	0.1	0.1
NF2	7348	7630	7699	6183	6393	6584	3790	3960	3621	3718	3720	1.0	0.8	0.5	0.5
SPOCD1	542	490	547	307	387	397	80	81	70	84	80	1.0	0.7	0.1	0.2
EFR3B	694	648	735	554	608	523	198	196	177	185	180	1.0	0.8	0.3	0.3
NPNT	11961	11595	11721	9955	9739	9429	3735	3544	3589	3509	3555	1.0	0.8	0.3	0.3
MRC2	9963	10121	10337	8119	7957	7901	2163	2181	2096	2015	1956	1.0	0.8	0.2	0.2
PREX1	22557	21999	22562	19198	18029	18646	7867	7822	7649	7192	7183	1.0	0.8	0.3	0.3
STMN3	2426	2504	2551	2148	2022	2081	1104	1078	1054	1057	1024	1.0	0.8	0.4	0.4
FSTL3	4573	4426	4469	3497	3679	3711	1764	1549	1659	1740	1560	1.0	0.8	0.4	0.4
AQP1	723	676	679	283	289	299	26	17	19	16	18	1.0	0.4	0.0	0.0
Mar-01	2166	2162	2146	1783	1878	1787	1200	1083	1142	1101	1103	1.0	0.8	0.5	0.5
IRX2	1871	2001	1986	1692	1643	1620	1284	1287	1192	1376	1231	1.0	0.8	0.6	0.7
ADAM19	354	385	412	321	251	282	158	159	157	167	134	1.0	0.7	0.4	0.4
PEAR1	46	54	63	27	38	33	4	8	4	5	6	1.0	0.6	0.1	0.1
IL11	104	169	144	81	75	84	12	19	15	20	24	1.0	0.6	0.1	0.2
ESPN	247	317	319	238	199	257	95	123	112	110	95	1.0	0.8	0.4	0.3
GADD45A	828	928	918	690	661	763	362	421	349	371	354	1.0	0.8	0.4	0.4
CCL22	66	61	94	34	32	34	4	2	5	2	7	1.0	0.5	0.0	0.1
ERN1	1462	1379	1483	1152	1243	1281	784	772	820	844	824	1.0	0.8	0.5	0.6
ADARB2	43	41	46	19	26	29	2	2	1	3	3	1.0	0.6	0.0	0.1
IL7R	82	68	69	35	31	37	1	3	3	3	2	1.0	0.5	0.0	0.0
SYT12	19684	19530	19897	14718	15351	14947	8359	8686	8529	8856	8782	1.0	0.8	0.4	0.4
LAMC2	10401	9633	10476	6780	7368	6925	3639	3792	3675	3699	3940	1.0	0.7	0.4	0.4
FLT1	109	103	101	59	56	55	13	14	9	16	15	1.0	0.5	0.1	0.1
AKAP12	573	555	646	267	281	297	49	46	27	41	50	1.0	0.5	0.1	0.1
TANC1	2049	2110	2126	1743	1633	1671	1152	1141	1104	1107	1144	1.0	0.8	0.5	0.5
FHOD1	2131	2095	2242	1802	1812	1897	1417	1431	1342	1432	1439	1.0	0.9	0.6	0.7
**KIAA0922**	**2096**	**2114**	**2185**	**1793**	**1719**	**1905**	**1353**	**1308**	**1263**	**1397**	**1367**	**1.0**	**0.8**	**0.6**	**0.6**
UNC5A	1540	1553	1673	1413	1181	1306	622	589	591	674	567	1.0	0.8	0.4	0.4
MIR24-2	313	349	367	281	269	286	121	117	115	135	104	1.0	0.8	0.3	0.3
KALRN	3036	2940	2950	2355	2389	2279	194	182	200	177	173	1.0	0.8	0.1	0.1
PCDH7	960	1067	1021	770	753	732	3	4	1	4	2	1.0	0.7	0.0	0.0

TMEM40	177	160	179	127	136	126	20	12	14	23	14	1.0	0.8	0.1	0.1
TP63	328	285	291	193	181	185	7	14	10	14	15	1.0	0.6	0.0	0.0
TIMP3	842	842	875	717	674	764	290	294	289	331	345	1.0	0.8	0.3	0.4
VAV1	203	193	192	156	168	128	16	16	26	38	17	1.0	0.8	0.1	0.1
VTN	250	250	259	202	190	181	13	14	21	24	14	1.0	0.8	0.1	0.1
PRKCG	298	303	308	222	204	193	5	13	10	23	8	1.0	0.7	0.0	0.1
LRRC8C	723	788	790	560	694	558	238	226	222	311	294	1.0	0.8	0.3	0.4
NTNG1	197	169	195	118	150	134	27	17	16	36	37	1.0	0.7	0.1	0.2
TGM2	3381	3100	3480	2736	2828	2846	1241	1123	1099	1622	1505	1.0	0.8	0.3	0.5
SH3TC1	50	59	37	26	30	20	4	1	2	2	2	1.0	0.5	0.0	0.0
ANKLE2	6875	7109	7692	5856	5942	6030	3625	3461	3406	3167	3364	1.0	0.8	0.5	0.5
CLDN1	12936	12777	13621	9699	10451	10375	4436	4152	4228	3624	3789	1.0	0.8	0.3	0.3
UNC13D	5897	5736	5890	4539	4474	4345	2208	2118	2122	1892	1968	1.0	0.8	0.4	0.3
EPHB6	2046	1951	2113	1326	1341	1282	478	435	464	380	380	1.0	0.6	0.2	0.2
DIP2B	7157	6865	7349	5549	5808	5928	3190	3168	3327	2818	3157	1.0	0.8	0.5	0.4
CSRNP2	3049	2848	3102	2237	2379	2390	1356	1317	1336	1214	1270	1.0	0.8	0.4	0.4
PALLD	20008	19894	20368	15280	16062	16146	9245	8672	9254	7959	8000	1.0	0.8	0.5	0.4
RIPK4	1514	1455	1626	1022	1102	1181	433	436	488	381	368	1.0	0.7	0.3	0.2
SGMS2	1998	2017	2051	1630	1700	1570	1079	1006	1101	912	1026	1.0	0.8	0.5	0.5
TSHZ3	205	222	213	163	152	180	60	52	68	46	61	1.0	0.8	0.3	0.3
LAMC1	11185	10928	11194	9256	9438	9494	6500	6498	6460	6147	6701	1.0	0.8	0.6	0.6
TLE2	2168	2052	2050	1671	1642	1729	971	1010	970	871	962	1.0	0.8	0.5	0.4
KRT20	1119	1020	1075	826	796	773	244	245	237	210	251	1.0	0.7	0.2	0.2
MAP1B	1821	1813	1804	1263	1456	1381	419	412	393	348	451	1.0	0.8	0.2	0.2
ZNF91	399	445	416	305	316	326	168	153	160	180	146	1.0	0.8	0.4	0.4
TRIM47	1312	1356	1337	872	890	1032	286	294	363	332	253	1.0	0.7	0.2	0.2
ZSWIM6	1208	1307	1318	995	1005	1052	505	502	544	520	521	1.0	0.8	0.4	0.4
TUBB2A	2731	2622	2772	2308	2266	2282	1565	1586	1637	1578	1418	1.0	0.8	0.6	0.6
SEC14L1	8764	8739	8985	6746	6755	6898	4267	4357	4307	4188	4119	1.0	0.8	0.5	0.5
PALM	1789	1781	1690	1376	1388	1395	932	915	930	943	884	1.0	0.8	0.5	0.5
ANXA2	63019	60591	60489	52372	51958	52157	38283	37193	37842	36301	35362	1.0	0.9	0.6	0.6
DIO3	289	280	276	203	200	176	51	38	72	60	46	1.0	0.7	0.2	0.2
SLC12A4	577	590	570	363	383	386	45	25	52	54	42	1.0	0.7	0.1	0.1
PLA2G4C	310	301	286	223	242	228	90	105	136	110	105	1.0	0.8	0.4	0.4
CHGA	78	56	64	36	41	43	2	4	6	4	6	1.0	0.6	0.1	0.1
MET	2047	1914	1985	1577	1769	1694	956	1004	1060	999	977	1.0	0.8	0.5	0.5
STX3	1823	1707	1749	1443	1449	1461	1586	1634	1659	1380	1409	1.0	0.8	0.9	0.8
TMEM45B	1339	1157	1257	834	867	927	1150	1055	1151	802	871	1.0	0.7	0.9	0.7
FAM63B	627	535	651	388	465	409	535	502	504	395	402	1.0	0.7	0.8	0.7
BMPR2	4438	4527	4363	3642	3799	3561	4082	3878	4164	3687	3734	1.0	0.8	0.9	0.8
SNX9	1595	1558	1501	1224	1312	1237	1325	1282	1445	1241	1251	1.0	0.8	0.9	0.8
FEZ2	1678	1627	1562	1344	1424	1366	1449	1535	1493	1250	1430	1.0	0.8	0.9	0.8
SNIP1	728	612	667	536	552	554	583	591	678	537	642	1.0	0.8	0.9	0.9
CAMK1	149	113	120	94	83	96	102	112	138	75	102	1.0	0.7	0.9	0.7
SP6	167	159	119	111	97	115	128	110	136	93	99	1.0	0.7	0.8	0.6
PLEKHO2	345	387	317	278	240	247	303	290	306	229	267	1.0	0.7	0.9	0.7
GRB7	328	408	325	237	248	230	267	247	305	186	254	1.0	0.7	0.8	0.6
LNX1	504	633	493	452	393	457	469	450	508	372	444	1.0	0.8	0.9	0.8
IRAK2	46	63	56	36	36	28	37	24	44	13	29	1.0	0.6	0.6	0.4
TAOK3	1416	1250	1292	1159	1067	1071	1106	1038	1147	899	1028	1.0	0.8	0.8	0.7
**SMPD1**	**594**	**639**	**558**	**538**	**457**	**516**	**455**	**491**	**510**	**372**	**442**	**1.0**	**0.8**	**0.8**	**0.7**
**BIK**	**344**	**391**	**367**	**302**	**251**	**301**	**277**	**255**	**291**	**194**	**258**	**1.0**	**0.8**	**0.7**	**0.6**
TNNT1	815	761	670	650	650	592	591	612	630	720	707	1.0	0.8	0.8	1.0
GBP3	182	215	154	144	140	109	141	147	151	169	146	1.0	0.7	0.8	0.9
TNS4	196	196	226	127	159	143	151	119	146	172	176	1.0	0.7	0.7	0.8
MIR29B1	80	46	78	32	43	23	31	25	30	38	21	1.0	0.5	0.4	0.4
NGEF	353	321	376	235	296	227	271	241	249	235	239	1.0	0.7	0.7	0.7
RNF213	5387	5207	5385	4350	4748	4059	4309	4238	4922	4141	4635	1.0	0.8	0.8	0.8
AHNAK	46111	47992	48154	37046	42048	37807	38472	38588	43390	37877	42408	1.0	0.8	0.8	0.8
FGD6	1050	1013	1050	747	830	816	792	773	857	710	809	1.0	0.8	0.8	0.7
CDYL	1643	1731	1708	1363	1442	1415	1443	1455	1543	1432	1492	1.0	0.8	0.9	0.9

n

n

n
